# [5]-Helistatins: Tubulin-Binding Helicenes with Antimitotic Activity

**DOI:** 10.1021/jacsau.2c00435

**Published:** 2022-10-19

**Authors:** James L. Rushworth, Aditya R. Thawani, Elena Fajardo-Ruiz, Joyce C. M. Meiring, Constanze Heise, Andrew J. P. White, Anna Akhmanova, Jochen R. Brandt, Oliver Thorn-Seshold, Matthew J. Fuchter

**Affiliations:** † Department of Chemistry, Molecular Sciences Research Hub, Imperial College London, White City Campus, 82 Wood Lane, London W12 OBZ, U.K.; ‡ Department of Pharmacy, Ludwig-Maximilians University of Munich, Munich 81377, Germany; § Cell Biology, Neurobiology and Biophysics, Department of Biology, Faculty of Science, Utrecht University, Utrecht 3584 CH, Netherlands

**Keywords:** cell cycle, DNA, helicene, mitosis, tubulin, antiproliferative, cancer

## Abstract

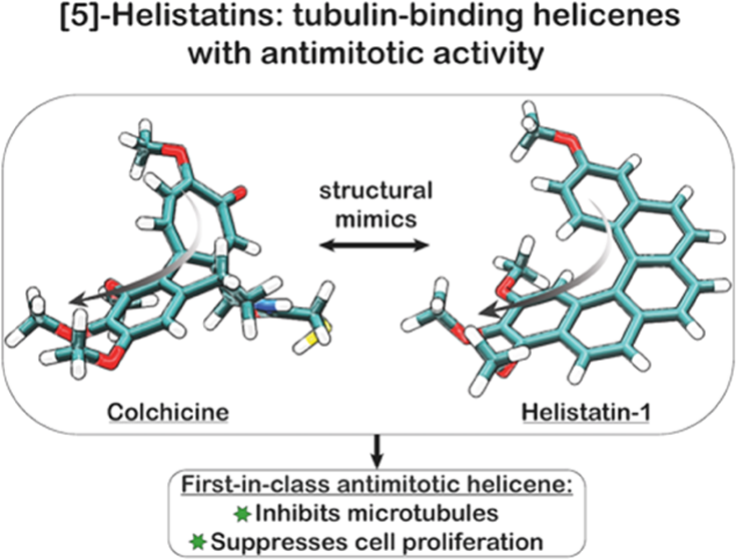

Helicenes are high interest synthetic targets with unique conjugated helical structures that have found important technological applications. Despite this interest, helicenes have had limited impact in chemical biology. Herein, we disclose a first-in-class antimitotic helicene, **helistatin 1 (HA-1)**, where the helicene scaffold acts as a structural mimic of colchicine, a known antimitotic drug. The synthesis proceeds *via* sequential Pd-catalyzed coupling reactions and a π-Lewis acid cycloisomerization mediated by PtCl_2_. **HA-1** was found to block microtubule polymerization in both cell-free and live cell assays. Not only does this demonstrate the feasibility of using helicenes as bioactive scaffolds against protein targets, but also suggests wider potential for the use of helicenes as isosteres of biaryls or *cis*-stilbenes—themselves common drug and natural product scaffolds. Overall, this study further supports future opportunities for helicenes for a range of chemical biological applications.

## Introduction

Helicenes are polycyclic molecules consisting of *ortho*-fused aromatic or heteroaromatic rings that are angularly arranged to give rise to a helically shaped conformation.^1^ The synthetic challenge of assembling such helically chiral aromatics has generated much interest, with new synthesis strategies and methodologies regularly reported.^2−13^ Furthermore, the coupling of a fully conjugated framework to a helical structure has led to a wide variety of applications for helicenes, including asymmetric catalysis,^14,15^ molecular switches,^16−18^ dyes,^19−22^ circularly polarized light emitters,^23,24^ spintronics,^25,26^ and organic electronic devices,^27−37^ to name a few. Despite the increasing number of applications for the use of helicenes in synthesis and materials science, these scaffolds are understudied in the area of chemical biology. Given their extended conjugation, there are several examples where the photophysical properties of helicenes have been exploited to enable them to act as dyes for cell imaging or photodynamic activity.^19,20,38−40^ Alternatively, and in analogy to more conventional polyaromatic hydrocarbons (PAHs), the cellular cytotoxicity of helicenes has been investigated in a few examples.^41−44^ However, there are limited examples of the helicene scaffold interacting specifically and directly with therapeutically relevant biological targets.

The best examples of helicenes designed for medicinal chemistry aim to address DNA topologies, including selective binding of helicenes to B/Z-DNA^45,46^ and G-quadruplex DNA.^40,41,47^ It is worth noting, however, that despite simple analogies between the helical form of both DNA and the helicenes, there is a large mismatch in helix dimensions: The helical pitch of [*n*]-helicenes with *n* = 6–11 is 3.21^48^ vs 33.2 Å for B-DNA.^49^ As such, helicene recognition of DNA is likely based on conventional mechanisms of binding, such as intercalation, rather than more specific shape complementarity. Herein, we show, to the best of our knowledge, the first helicene specifically designed to interact with a non-DNA (protein) target, tubulin.

Microtubules (MTs) are intracellular protein scaffolds used by all eukaryotic cells to support mechanical processes, including cell motility, serving as tracks for cargo transport by motor proteins, and separating chromosomes in cell division.^50^ Structurally, MTs are giant noncovalent polymers of the α/β-tubulin ‘monomer’ protein, and in cells, they are continuously remodeled through regulated cycles of polymerization and depolymerization to fulfill their functions.^51,52^ Tubulin-binding drugs that disrupt MT de/polymerization rates therefore inhibit MT-dependent cellular processes: both MT stabilizers (taxanes, epothilones) and depolymerizers (colchicine, vinca alkaloids) are potent *in vivo*-active drugs.^53^ In cell culture, such compounds characteristically suppress MT polymerization dynamics and block cell proliferation.^54,55^

**Figure 1:**
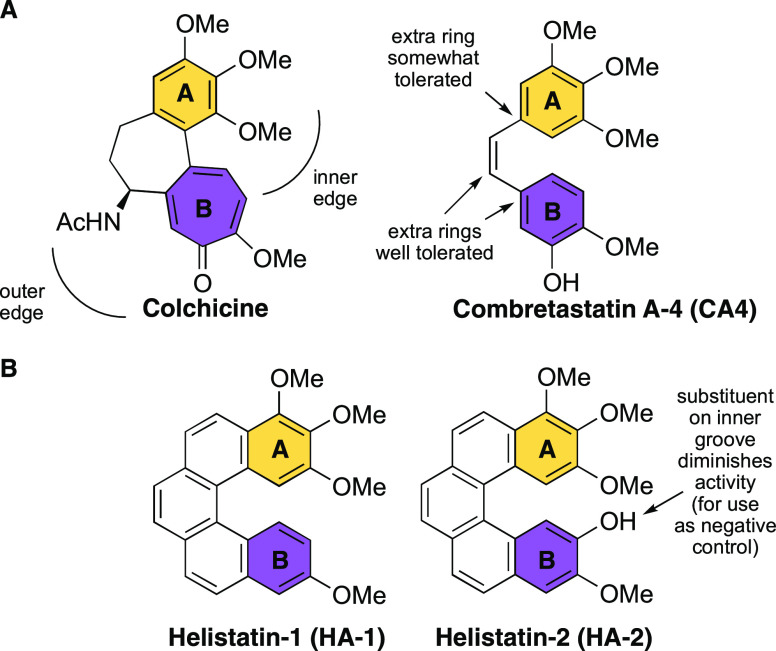
(A) Structures of colchicine and **CA4**. An SAR overview for **CA4** is provided. (B) Syntheses of **HA-1** and **HA-2**, both structural analogues of **CA4**, are reported in this work. **HA-1** was designed as the active tubulin inhibitor and **HA-2**, where the *ortho* electron-donating −OH is located on the inner groove of the helicene, was designed to act as a negative control.

A myriad of MT inhibitors are known to bind at the so-called colchicine-domain of β-tubulin. The prototypical colchicine-domain inhibitor (CDI) pharmacophore, shared by colchicine and similar compounds such as stilbene combretastatins,^56^ is a 3,4,5-trimethoxyaryl “A” ring, held twisted near a mono-methoxyaryl “B” ring (Figure 1A). The B ring can tolerate small polar substituents (OH, F, NH_2_) on the outer edge, whereas substituents on the inner edge entirely block binding.^57,58^ “Bridge” rings and annulations to the B ring are well tolerated, although annulations at the A ring lower potency.^59^ Combretastatin A-4 (**CA4**), isolated from the African willow tree *Combretum caffrum*, has been found to possess particularly promising anti-tubulin activity.^60^**CA4** binds to the colchicine-binding site of β-tubulin and has a higher level of bioactivity in its *cis*-form;^61^ however, **CA4** is prone to configurational instability and can easily isomerize to the inactive *trans* isomer in the presence of light, heat, or protic media.^62−64^ While several conformationally restricted analogues of **CA4** have been developed,^65−70^ we envisaged that the angular disposition of the A and B rings, coupled with the need to lock them in a cisoid configuration, would be well suited to a [5]-helicene.

Herein, we report our first designs of helicene analogues of the combretastatins: the helistatins (Figure 1B). Our design (**HA-1**), partly informed by our synthetic strategy (*vide infra*), is based on an analogue of **CA4**, originally developed by Cushman and co-workers, whereby the nonessential *meta-*hydroxyl group in **CA4** was removed.^71^ We coupled our active design with a suitable negative control (**HA-2**), with a substituent on the inner groove to reduce activity.^72^ Siles and co-workers showed that the presence of two electron-withdrawing groups (*i.e.*, NO_2_) *ortho* to the methoxy diminished tubulin inhibition (IC_50_) from 1.2 μM (**CA4**) to 7.4 μM. This effect was exacerbated when the NO_2_ groups were converted to electron-donating anilines (IC_50_ > 40 μM).^73^ Based on this rationale, we added a closely matching electron-donating phenol to this position. We find that **HA-1** blocks microtubule polymerization in both cell-free and live cell assays and, as such, represents the first example of a specific protein-targeting helicene.

## Results and Discussion

**Figure 2:**
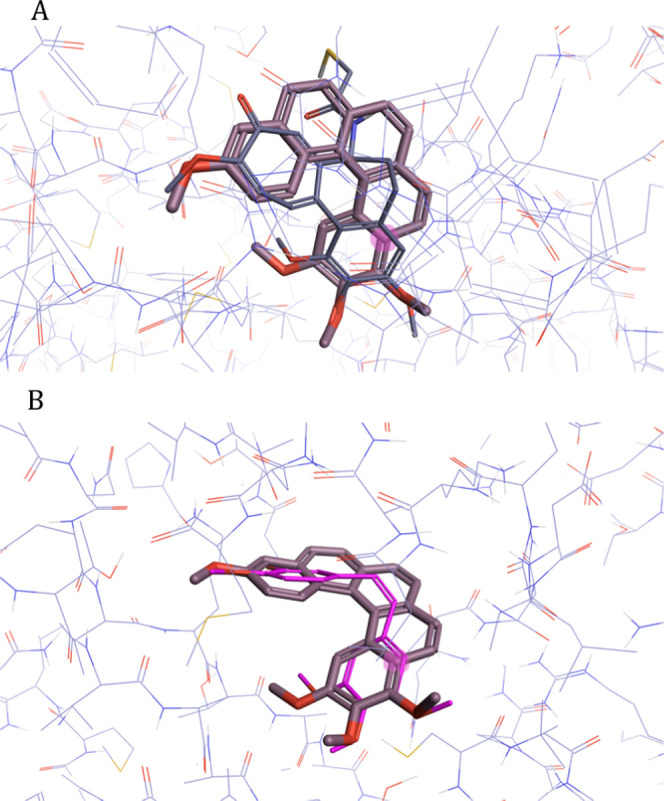
Molecular docking of (*P*)-**HA-1** against (A) DAMA-colchicine^77^ and (B) **CA4** in the tubulin-colchicine stathmin-like domain complex (PDB code: 1SAO). There is excellent overlap in both polar and nonpolar interactions, and the helical pitch provides the correct conformation for binding. See Movies S1–S4 to visualize the full three-dimensional fit.

To gauge the general suitability of [5]-helistatins for interaction with the colchicine-binding site of tubulin, we conducted computational docking experiments.^74−76^ The tubulin-colchicine stathmin-like domain complex (PDB code: 1SAO) was chosen as the model target (Figure 2). There is a good three-dimensional (3D) overlap between the energy-minimized structure of (*P*)-**HA-1** and both a colchicine analogue (DAMA-colchicine, Figure 2A)^77^ and **CA4** (Figure 2B). Overall, the docking studies show excellent complementarity for the colchicine-binding site; both polar and nonpolar interactions were retained. For completeness, we also modeled **HA-1** against colchicine in the tubulin-colchicine complex (PDB code: 4O2B),^78^ and we found the same level of complementarity (Figure S1). The full three-dimensional fit of these molecules can be visualized in Movies S1–S4.

Despite the promise of this design, the [5]-helistatins proved to be extremely challenging to prepare with respect to many of the conventional methods used for helicene synthesis. The highly functionalized, electron-rich, and asymmetric nature of the terminal rings often prevented the formation of the desired product at, or close to, the final step of numerous attempted routes. For completeness, these unsuccessful synthesis strategies are outlined in the Supporting Information. Briefly, initial efforts were focused on formation of the helicene *via* oxidative photocyclization; however, the final photoisomerization step afforded a furan (Scheme S1) instead of the desired helicene.^79^ Various protecting group strategies were employed, although these only resulted in decomposition. Oxidative cyclization was then examined (Scheme S2), although this also led to the formation of decomposition products.^80−82^ We then employed radical methodology^83^ and successfully synthesized the target phenanthrene/stilbene intermediate (Scheme S3); however, attempts at performing the final ring closure resulted in either dehalogenation or decomposition. Finally, we turned our attention to cycloisomerization methodology, first *via* a [2 + 2 + 2] cycloisomerization (Scheme S4),^84^ and finally, a π-Lewis acid cycloisomerization mediated by PtCl_2_, which is the methodology described herein.

**Scheme 1:**
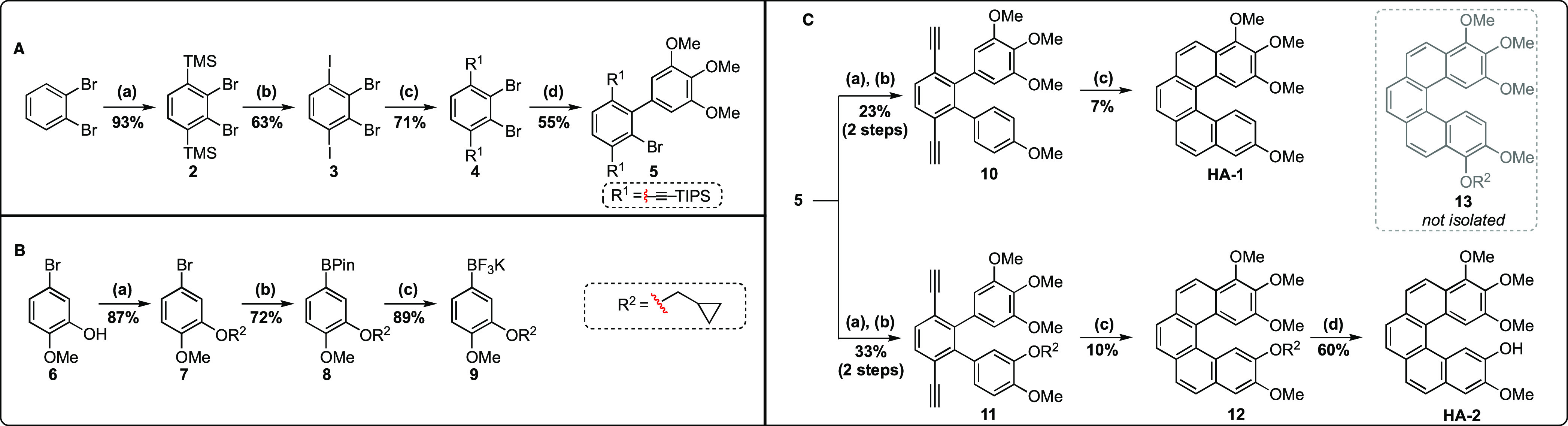
(A) Reaction conditions: (a) LDA, TMSCl, and THF, −78–0 °C, 24 h; (b) **2**, ICl and CH_2_Cl_2_, −10–0 °C, 18 h; (c) **3**, TIPS-Acetylene, CuI (10 mol %), Pd(dppf)Cl_2_ (5 mol %), and PhMe/DIPA (2:1), 70 °C, 6 h; (d) **4**, (3,4,5-trimethoxyphenyl)boronic acid, Pd(dppf)Cl_2_ (12 mol %), K_2_CO_3_ and 1,2-Dimethoxyethane/H_2_O (10:1), 85 °C, 18 h. (B) Reaction conditions: (a) **6**, cyclopropyl methyl bromide, K_2_CO_3_, THF, 70 °C, 18 h; (b) **7**, nBuLi, 2-isopropoxy-4,4,5,5-tetramethyl-1,3,2-dioxaborolane, −78–0 °C, 24 h; (c) KF (10 M aq.) MeCN/MeOH (1:1), 21 °C, 1 min, then l-(+)-tartaric acid in THF, 2–5 min, then filter. (C) Reaction conditions: (a) 4-methoxyphenylboronic acid or **9**, Pd(dppf)Cl_2_ (10 mol %), K_2_CO_3_ or Cs_2_CO_3_, and 1,2-dimethoxyethane/H_2_O (10:1), 85 °C, 18 h; (b) TBAF, THF, r.t., 2 h; (c) PtCl_2_ (40 mol %), PhMe, 80 °C, 18 h; (d) 12 M HCl, MeOH/iPrOH (1:2), 60 °C, 18 h.

Through knowledge gained in our extensive survey of relevant chemistry, we ultimately managed to achieve the successful synthesis of [5]-helistatins **HA-1** and **HA-2** in a modular and semi-two directional fashion *via* common intermediate **5** (Scheme 1A). Initially, 1,2-dibromobenzene was subjected to a regiospecific *ortho*-lithiation protocol with the subsequent aryl lithium being trapped *in situ* with TMSCl to generate the bis-silylated species **2**. The identity of **2** was confirmed by X-ray crystallography. The protocol developed for this reaction, a modification of earlier work by Serwatowski and Lulinski, does not generate any monosilylated product.^85^ Iodo-desilylation of **2** provided **3** in 63% yield. The subsequent Sonogashira coupling, however, proved capricious with significant batch-to-batch variations in product yield. These issues were traced to elemental sulfur contamination, confirmed *via* X-ray crystallography (Figure S2), stemming from the Na_2_S_2_O_4_ quench utilized in the iodo-desilylation step. It was noted that, at room temperature, a PhMe/DIPA solution of **3** would rapidly turn black when brought into contact with the Cu(I) utilized in the Sonogashira coupling. We presume that this is the result of Cu*_x_*S*_y_* species being formed *in situ*, which in turn do not participate in the catalytic cycle. From this key observation, we devised a procedure to purify the precursor material using acid-activated Cu(0) turnings to sequester any elemental sulfur. This operationally simple purification ensured that the Sonogashira coupling was both robust and reproducible. As an aside, all steps up to this point in the synthesis do not require involved chromatographic purification; instead purification can be achieved primarily by re-crystallizations and/or silica plugs. With access to multigram quantities of **4**, derivatization into the two helicene precursors **10** and **11** proceeded *via* sequential Suzuki–Miyaura cross-couplings. To obtain **11**, the trifluoroborate salt **9** was utilized, featuring a cyclopropyl methyl ether (CME) protecting group (Scheme 1B,C, see Figure S5 for crystal structure). Our choice of this rather uncommon protecting group was guided by the need for it to be sufficiently stable to lithiation conditions while not interfering with the final Pt-mediated cyclization step and simultaneously to be easy to remove (*vide infra*). The bis-alkynes **10** and **11** are, thus, primed for a PtCl_2_-mediated cyclization to the desired helicenes (Scheme 1C). Pt(II) and Pt(IV) complexes have been used in the analogous syntheses of substituted phenanthrenes and helicenes through judicious use of similar alkyne handles.^30,86^ Thus, **10** was converted to a complex mixture from which only **HA-1** could be obtained. It is likely that this mixture contained compounds that result from both incomplete ring closure and ring closure at an undesired position, forming structures such as azulenes^87^ and fluorene derivatives,^88^ and hence the relatively low yield. Similarly, PtCl_2_-mediated cyclization of **11** should, theoretically, enable access to **HA-2** and its regioisomer **13**; however, a complex mixture from which only **HA-2** was obtained: regioisomer **13** could not be isolated. We propose that, due to steric interactions with the CME group, cyclization to yield **13** is sterically disfavored. Instead, cyclization at the less-hindered position is preferred and leads to **12**. Deprotection of the CME group on **12** proceeded smoothly using 12 M HCl in MeOH/iPrOH and provided **HA-2**.

**Figure 3:**
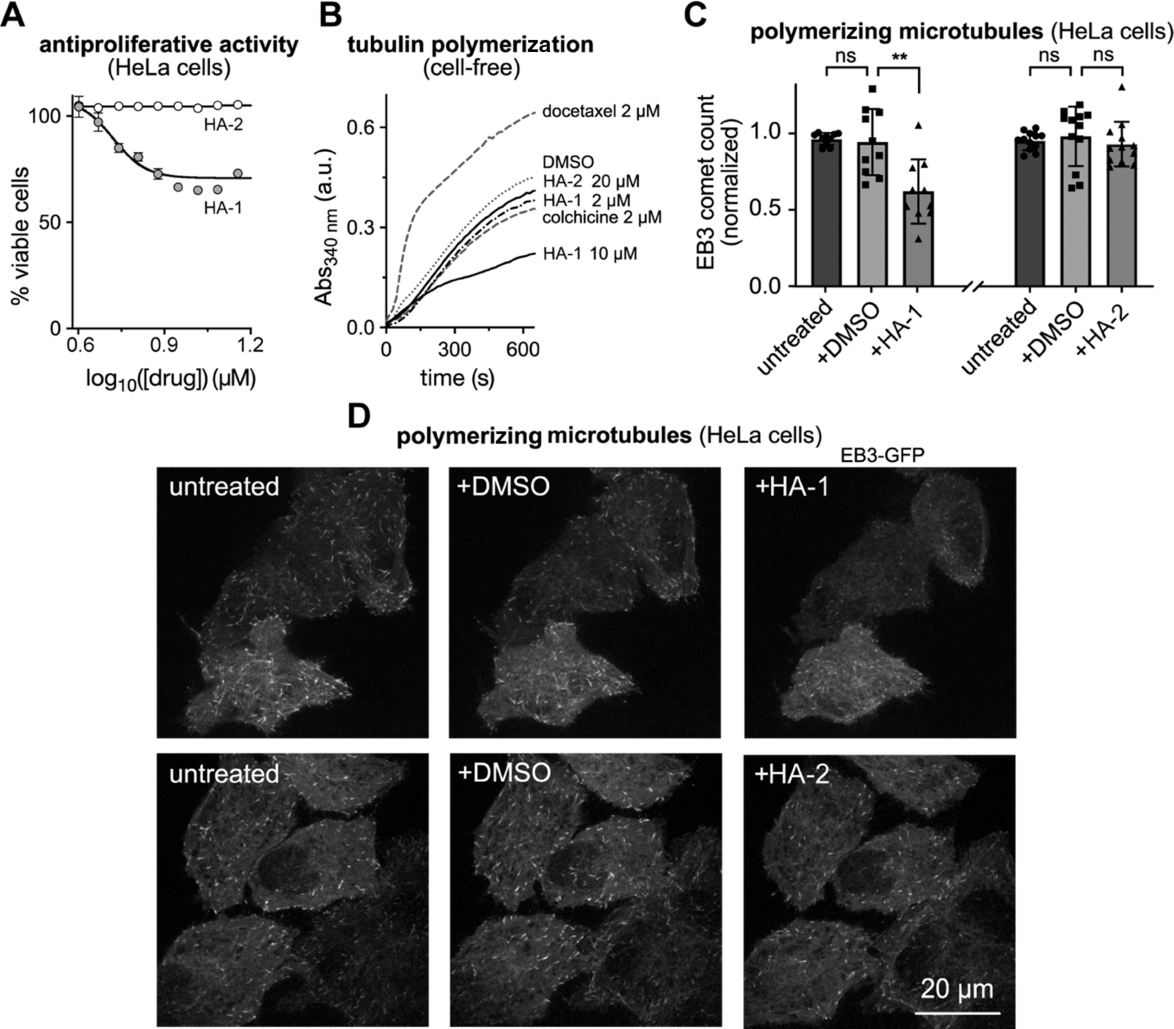
Cellular bioactivity and mechanism of action of **HA-1**. (A) Antiproliferative activity (HeLa cells, 40 h incubation; one experiment representative of three independent experiments is shown). (B) Suppression of tubulin polymerization in cell-free conditions. Docetaxel is a positive control for polymerization enhancement, roughly indicating the magnitude of a meaningful difference from DMSO-only control behavior. **HA-1** at 10 μM shows meaningful polymerization inhibition, while **HA-2** up to 20 μM and colchicine or **HA-1** at the substoichiometric concentration of 2 μM are all insignificantly inhibiting conditions (turbidimetric assay; greater absorbance indicates a greater degree of polymerization). (C, D) Live cell EB3-GFP “comets”, indicating growing MTs during **HA-1/HA-2** treatments (HeLa cells). Cells were first imaged in media for baseline MT dynamics, then treated with 1% DMSO and imaged again, then 20 μM **HA-1/HA-2** was applied, and the same cells were again imaged, which allows longitudinal comparisons over each treatment (10/12 cells acquired for **HA-1/HA-2**). (C) Each cell’s comet count was normalized to that in the first five frames of the no-cosolvent control before statistics (Wilcoxon) calculated for groups; mean ± SD with data points overlaid. (D) Stills from representative movies, Movies S5 and S6, showing that **HA-1** suppresses polymerizing microtubule count, but **HA-2** does not.

MT inhibitors exert antiproliferative activity by blocking cell division (mitosis). We evaluated the antiproliferative effects of **HA-1** in a HeLa human cervical cancer cell line, assessing cell viability after 40 h. **HA-1** was found to suppress ca. 40% of typical cell proliferation at low micromolar concentrations with a well-formed sigmoidal dose–response curve, featuring a Hill slope typical for tubulin-depolymerizing agents (Figure 3A). The plateauing response above 10–20 μM is typical for lipophilic compounds applied near their solubility limits. **HA-2** was entirely inactive up to this concentration (Figure 3A). This observation supports our initial hypothesis that SAR trends for colchicine-derived inhibitors are translatable to [5]-helistatins, including that the addition of the −OH on the inner groove of **HA-2** diminishes its activity, as predicted. To overcome the limitations with solubility, a prodrug approach akin to those used with **CA4** might be appropriate. This could include conversion of the phenol to the phosphate salt,^89^ or functionalization with an aminohydroxy moiety.^90^

To test the mechanism of **HA-1** bioactivity in a cell-free system, we examined its ability to suppress polymerization of purified tubulin protein. **HA-1** was a strong polymerization suppressor at 10 μM (whereas **HA-2** was entirely inactive up to 20 μM). This both matches the cellular activity data and confirms the ability of **HA-1** to inhibit tubulin protein directly (Figure 3B).

Finally, we examined whether **HA-1** maintains its MT-inhibitory activity in cells. We imaged live cells transfected with the fluorescently labeled MT end binding protein EB3-GFP, which marks the polymerizing plus ends of MTs as dynamic “comets”, by confocal microscopy.^91^ EB3 imaging sensitively and quantitatively reveals inhibition of typical MT dynamics by inhibitors.^92^ Cells were first imaged upon treatment with DMSO alone, to establish baseline MT dynamics; then **HA-1** was applied at 20 μM, significantly suppressing the polymerization of MTs (to 60% of control; Figure 3C, D and Movie S5). Controls with **HA-2** under the same conditions showed no inhibition, as expected (Figure 3C and Movie S6). These assays show that helicene **HA-1** is an effective inhibitor of microtubule dynamics in live cells, and that its effect is not a result of the nonspecific properties of the helicene scaffold but is due to its specifically designed pharmacophore.

The metabolic fate of **CA4** and its analogues are known to include quinone species, which retain strong inhibitory activity against tubulin polymerization.^93^ However, the experiments utilizing purified tubulin protein (Figure 3B), where oxidative metabolism is not possible, show that the observed bioactivity of **HA-1** is inherently a feature of the helicene scaffold.

Given the intrinsic helical chirality of **HA-1**, we performed a chiral HPLC separation of (*P*)- and (*M*)-**HA-1**. (*P*)-**HA-1** was found to have an enantiomerization half-life of 1.6 h at 37.5 °C (Figure S6). The judicious addition of methyl groups on the B ring of **HA-1** might offer one way to reduce its conformational flexibility.^94^

## Conclusions

In summary, we report the first helicene designed to bind to a defined and therapeutically relevant protein target, with bioactivity at cellularly relevant concentrations and mechanistically validated using a stringent live cell imaging assay. While there is significant room for improvement in the potency of **HA-1**, this molecule nonetheless acts as a valuable proof of concept example toward helicene-based bioactive agents. We anticipate that our study will motivate further use of helicene-type bioactive reagents for chemical and cell biology, especially where conformationally flexible biaryl/stilbenoid reagents are already known.^95^

## Methods

### Docking Methodology

Docking studies were carried out with Flare, version 5, Cresset, Litlington, Cambridgeshire, U.K..^74−76^ The crystal structure files (PDB: 1SAO and 4O2B) were downloaded directly from the protein data bank. Default protein preparation was carried out to set charge states and tautomers and to define the reference molecules (DAMA-colchicine and colchicine for 1SAO and 4O2B, respectively). The ligands **CA4** and (*P*)-HA-1 were then imported and were subjected to energy minimization to ascertain the most suitable conformers. These energy-minimized structures were then aligned to the reference molecules *via* molecular field and shape-guided substructure alignment. The pdb file for each ligand is provided in the Supporting Information (Files S7–S10). The ligand coordinates (calculated with PyMOL) are as follows: **HA-1** [17.273, 65.702, 42.681]; HA-2 [18.386, 64.438, 42.211]; colchicine [16.981, 65.997, 43.482]; and **CA4** [16.956, 65.867, 42.621].

### General Synthetic Methodology

All reagents and solvents were purchased from commercial sources and used as supplied unless otherwise indicated. All reactions were carried out under an inert atmosphere, using anhydrous solvents. All reactions were monitored by thin-layer chromatography (TLC) using Merck silica gel 60 F254 plates (0.25 mm). TLC plates were visualized using ultraviolet (UV) light (254 nm) and/or an appropriate TLC stain. Silica column chromatography was performed using Merck Silica Gel 60 (230–400 mesh) treated with a solvent system specified in the individual procedures. Solvents were removed by a rotary evaporator, and the compounds were further dried using high-vacuum pumps. Infrared spectra were recorded neat on an Agilent Cary 630 FTIR. Reported absorptions are in wavenumbers (cm^–1^). ^1^H and ^13^C NMR were recorded on a Bruker Advance 400 spectrometer at 400 and 100 MHz, respectively. Chemical shifts (δ) are quoted in ppm (parts per million) downfield from tetramethylsilane, referenced to residual solvent signals. The following abbreviations are used to designate multiplicity within ^1^H NMR analysis: s = singlet, d = doublet, t = triplet, q = quartet, m = multiplet, and br. = broad signal. High-resolution mass spectra (ESI, APCI) were recorded by the Imperial College London Department of Chemistry Mass Spectroscopy Service using a Micromass Autospec Premier and Micromass LCT Premier spectrometer.

Full synthetic details are outlined in the Supporting Information.

### General Procedure for the Final Helicene Forming Step

PtCl_2_ (1 equiv) was added to a solution of alkyne (2.5 equiv) in degassed PhMe (to give an alkyne concentration of 60 mM). The reaction was heated to 80 °C for 18 h. Subsequently, the reaction was concentrated *in vacuo* and purified directly *via* Si column chromatography with various mixtures of Et_2_O in hexane to afford the title compound.

### Resazurin Antiproliferation Assay

HeLa human cervical cancer cells were sourced from ATCC and maintained under standard cell culture conditions in Dulbecco’s modified Eagle’s medium (DMEM; PAN-Biotech: P04-035550) supplemented with 10% fetal calf serum (FCS), 100 U/mL penicillin, and 100 U/mL streptomycin. Cells were grown and incubated at 37 °C in a 5% CO_2_ atmosphere. Cells were seeded in 96-well plates at 10,000 cells/well and left to adhere for 24 h, before treating with test compounds for 48 h (final well volume 100 μL, 1% DMSO; three technical replicates); the cosolvent control was treated with DMSO only. Cells were then treated with resazurin for 3 h to measure metabolic activity (through reduction to fluorescent resorufin) as a readout for live cell count.^96^ Fluorescence was measured at 590 nm (excitation 544 nm) using a FLUOstar Omega microplate reader (BMG Labtech). Absorbance data were normalized to viable cell count from the cosolvent control cells as 100% viability, where 0% viability was set to correspond to fluorescence signal from PBS with no cells, treated with resazurin (this underestimates true values corresponding to “no live cells” by *ca.* 5–15%, but does not affect assay outcomes and interpretation). Three independent experiments were performed. Viability data were plotted against the log of compound concentration (log_10_([drug]) (M)). One representative HeLa experiment out of three is shown as Figure 3A (one data point per technical replicate). HA-1 shows antiproliferative activity, while HA-2 does not.

### Cell-Free Tubulin Polymerization Assay

Tubulin (99% purity) from the porcine brain was obtained from Cytoskeleton Inc. (cat. #T240). Polymerization was performed at 5 mg/mL tubulin, in polymerization buffer BRB80 (80 mM piperazine-*N*,*N*′-bis(2-ethanesulfonic acid) (PIPES) pH = 6.9; 0.5 mM EGTA; 2 mM MgCl_2_), in a cuvette (120 μL final volume, 1 cm path length) in a Agilent CaryScan 60 with a Peltier cell temperature control unit maintained at 37 °C, with glycerol (10 μL). Tubulin was first incubated for 5 min at 37 °C with the test compound in buffer with 3% DMSO, without GTP. Then GTP was added (1 μL spike, with mixing, final GTP concentration 1 mM) to initiate polymerization, and the change in absorbance at 340 nm due to scattering from the turbid medium was monitored (greater turbidity = more polymerization).

### Cellular Microtubule Dynamics Imaging

HeLa cells were transfected with EB3-GFP using FuGENE 6 (Promega) according to the manufacturer’s instructions. Experiments were imaged on a Nikon Eclipse Ti microscope equipped with a perfect focus system (Nikon), a spinning disk-based confocal scanner unit (CSU-X1-A1, Yokogawa), an Evolve 512 EMCCD camera (Photometrics) attached to a 2.0× intermediate lens (Edmund Optics), a Roper Scientific custom-made set with 487 nm (150 mW) laser, ET-GFP filter (Chroma), a motorized stage MS-2000-XYZ, a stage top incubator INUBG2E-ZILCS (Tokai Hit), and lens heating calibrated for incubation at 37 °C with 5% CO_2_. Microscope image acquisition was controlled using MetaMorph 7.7, and images were acquired using a Plan Apo VC 40× NA 1.3 oil objective. Imaging conditions were initially confirmed to minimize GFP bleaching and phototoxicity in untreated cells. For compound-treated acquisitions, a compound diluted in prewarmed cell medium was applied to cells and incubated on cells for at least 1 min before commencing acquisition. Comet count analysis was performed in ImageJ using the ComDet plugin (Katrukha 2020, ComDet plugin for ImageJ, v0.5.3, Zenodo, doi: 10.5281/zenodo.4281064). Blinding was not performed as assay readout is unbiased (Fiji/ImageJ plugins). Data were analyzed using Prism 9 software (GraphPad).

## Supporting Information Available

Computational simulation of DAMA-colchicine in the stathmin-like domain complex (PDB code: 1SAO),^77^ overlayed with **HA-1**, followed by **CA4** (Movie S1) (MP4)
Computational simulation of DAMA-colchicine in the stathmin-like domain complex (PDB code: 1SAO),^77^ overlayed with **HA-1**, followed by **CA4** (Movie S2) (MP4)
Computational simulation of colchicine in the tubulin-colchicine complex (PDB code: 4O2B),^78^ overlayed with **HA-1**, followed by **CA4** (Movie S3) (MP4)
Computational simulation of colchicine in the tubulin-colchicine complex (PDB code: 4O2B),^78^ overlayed with **HA-1**, followed by **CA4** (Movie S4) (MP4)
Inhibition of microtubule dynamics by **HA-1** (data related to Figure 3); no-cosolvent, and 1% DMSO, control phases (baseline dynamics) precede the **HA-2** phase (as indicated by captioning; 20 μM HA-1; EB3-GFP transiently transfected HeLa cells) (Movie S5) (MP4)
No inhibition of microtubule dynamics by **HA-2** (data related to Figure 3); no-cosolvent, and 1% DMSO, control phases (baseline dynamics) the precede **HA-2** phase (as indicated by captioning; 20 μM HA-2; EB3-GFP transiently transfected HeLa cells) (Movie S6) (MP4)
4O2B PDB file with **HA-1** bound as a ligand (File S7) (PDB)
4O2B PDB file with **HA-2** bound as a ligand (File S8) (PDB)
4O2B PDB file with colchicine bound as a ligand (File S9) (PDB)
4O2B PDB file with **CA4** bound as a ligand (File S10) (PDB)
Crystal structure data (CIF)
Molecular docking studies; alternative synthetic routes considered; synthetic methodology and associated analytical data (including NMR and X-ray crystallography data); chiral HPLC purification data of HA-1; and enantiomerization kinetics; biological data including the resazurin antiproliferation assay; cell-free tubulin polymerization assay, and cellular microtubule dynamics imaging (PDF)

## Supplementary Material

au2c00435_si_006.mp4

au2c00435_si_007.pdb

au2c00435_si_008.pdb

au2c00435_si_009.pdb

au2c00435_si_010.pdb

au2c00435_si_011.cif
