# Supplementary material for: [5]-Helistatins: Tubulin-Binding Helicenes with Antimitotic Activity
Source: JACS Au. 2022 Oct 19;2(11):2561–70. doi: 10.1021/jacsau.2c00435 (PMC9709948; doi:10.1021/jacsau.2c00435)
Supplement: Supplementary file 12 — au2c00435_si_012.pdf [file au2c00435_si_012.pdf]

[5]-Helistatins: Tubulin binding helicenes with anti-mitotic activity

Supporting Information

James L. Rushworth,[a]†₸ Aditya R. Thawani,[a]† Elena Fajardo-Ruiz,[b]± Joyce C. M. Meiring,[c] Constanze Heise,[b]Π Andrew J. P. White,[a] Anna Akhmanova,[c] Jochen R. Brandt,[a] Oliver Thorn-Seshold,[b] and Matthew J. Fuchter[a]*.

[a] Dr. J. L. Rushworth, Dr. A. R. Thawani, Dr. A. J. P. White, Dr. J. R. Brandt, Prof. M. J. Fuchter

Department of Chemistry, Molecular Sciences Research Hub, Imperial College London,

White City Campus, 82 Wood Lane, London W12 OBZ, United Kingdom. E-mail: m.fuchter@imperial.ac.uk

[b] E. Fajardo-Ruiz, C. Heise and Dr. O. Thorn-Seshold

Department of Pharmacy, Ludwig-Maximilians University of Munich, Munich 81377, Germany

[c] Dr. J. C. M. Meiring, Prof. Anna Akhmanova

Cell Biology, Neurobiology and Biophysics, Department of Biology, Faculty of Science,

Utrecht University, Utrecht 3584 CH, Netherlands

+These authors contributed equally

₸ J.L.R. is presently employed at Sixfold Bioscience, Translation & Innovation Hub, 84 Wood Lane, London, W12 0BZ, UK.

± E.F-R. is presently employed at the Faculty of Biology, Ludwig-Maximilians University of Munich, Munich 81377, Germany.

Π C.H. is presently employed at Cambrium GmbH Max-Urich-Straße 3, Berlin, 13355, G

Table of Contents

1. Additional studies of the [5]-helistatins *2*

1.1 Molecular docking and predictive properties of [5]-helistatin, HA-1 2

2. Alternative synthetic routes considered *7*

2.1 Oxidative photocyclization 7

2.2 Oxidative cyclization 8

2.3 Radical cyclization 9

2.4 [2+2+2] cycloisomerization 10

3. Synthetic methodology *11*

3.1 General experimental 11

3.2 Synthetic procedures 12

3.1 Purification of 3 with Cu(0) turnings 26

3.2 Chiral HPLC Purification of HA-1 27

3.3 X-Ray Crystallography 29

3.4 Enantiomerisation Kinetics 31

4. Biological assays *33*

4.1 Resazurin antiproliferation assay 33

4.2 Cell-free tubulin polymerisation assay 33

4.3 Cellular microtubule dynamics imaging 33

5. NMR Spectra *35*

6. References *66*

1. Additional studies of the [5]-helistatins

1.1 Molecular docking and predictive properties of [5]-helistatin, HA-1

Docking methodology

Docking studies were carried out with Flare, version 5, Cresset®, Litlington, Cambridgeshire, UK; http://www.cresset-group.com/flare/.[1–3] The crystal structure files (PDB: 1SAO and 4O2B) were downloaded directly from the protein data bank. Default protein preparation was carried out to set charge states and tautomers and to define the reference molecules (DAMA-colchicine and colchicine for 1SAO and 4O2B, respectively). The ligands CA4 and *(P)*-HA-1 were then imported and were subjected to energy minimisation to ascertain the most suitable conformers. These energy-minimised structures were then aligned to the reference molecules *via* molecular field and shape-guided substructure alignment. The pdb file for each ligand is provided as supplementary information (**Files S7 to S10**). The ligand coordinates (calculated with PyMOL) are as follows: HA-1 [17.273, 65.702, 42.681]; HA-2 [18.386, 64.438, 42.211]; colchicine [16.981, 65.997, 43.482]; CA-4 [16.956, 65.867, 42.621].

Figure S1 A Compounds used in the docking studies; **B** Overlap of **HA-1** with DAMA-colchicine and CA4 (**C**) in the stathmin-like domain complex (PDB code: 1SAO).[4] There are few polar interactions, which is in accordance with that observed in the co-crystalized structure of colchicine. The main polar interaction is between the Cys241 SH group and one of the methoxy groups on the A-ring, which is also present on the ligand. Key non-polar interactions between the B-ring and Thr179 and Ser178 are also present. **D** overlap of **HA-1** with colchicine and CA4 (**E**) in the *tubulin-colchicine complex (PDB code: 4O2B).*[5]

A)

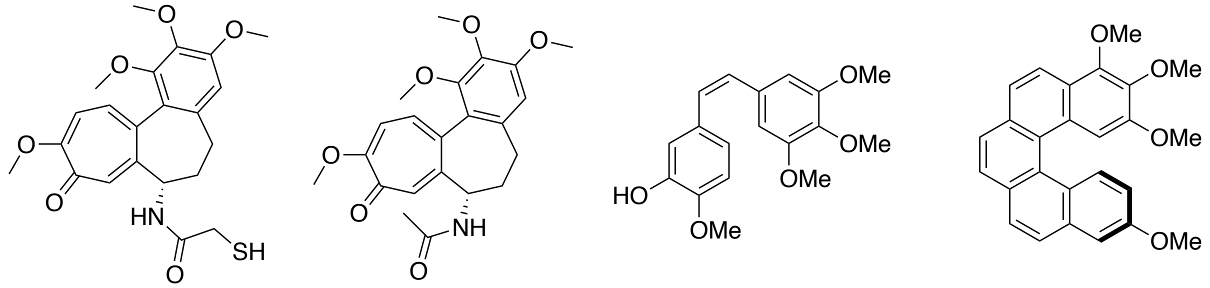

DAMA-colchicine colchicine CA4 (*P*)-HA-1

Stathmin-like domain complex (PDB code: 1SAO)[4]

B) DAMA-colchicine

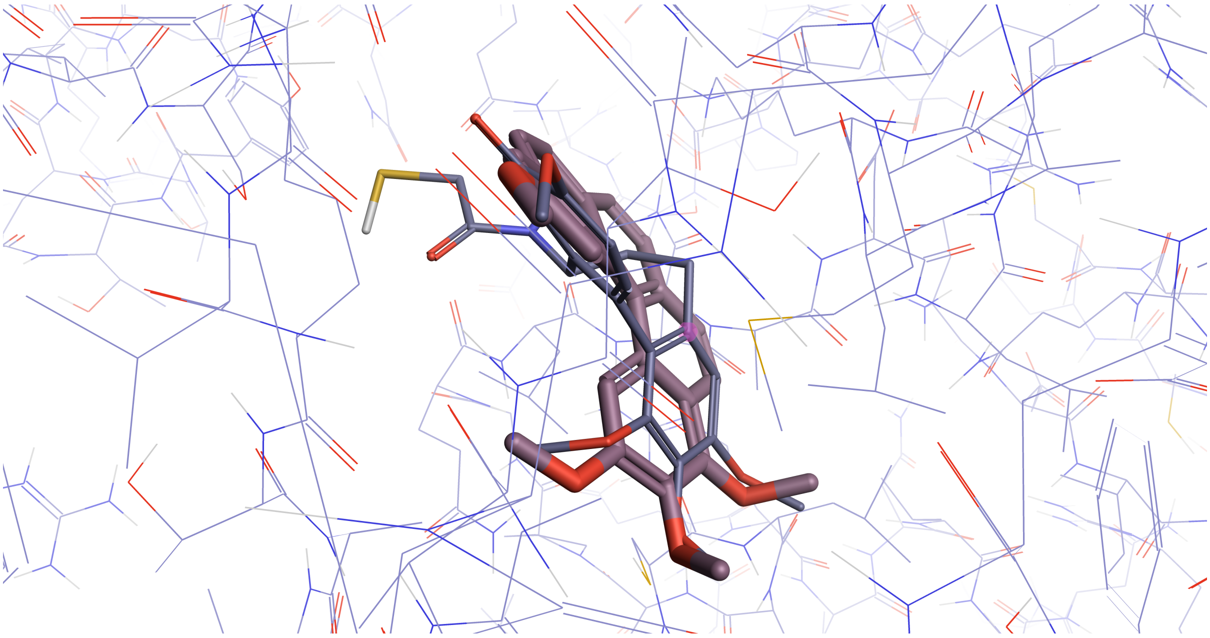

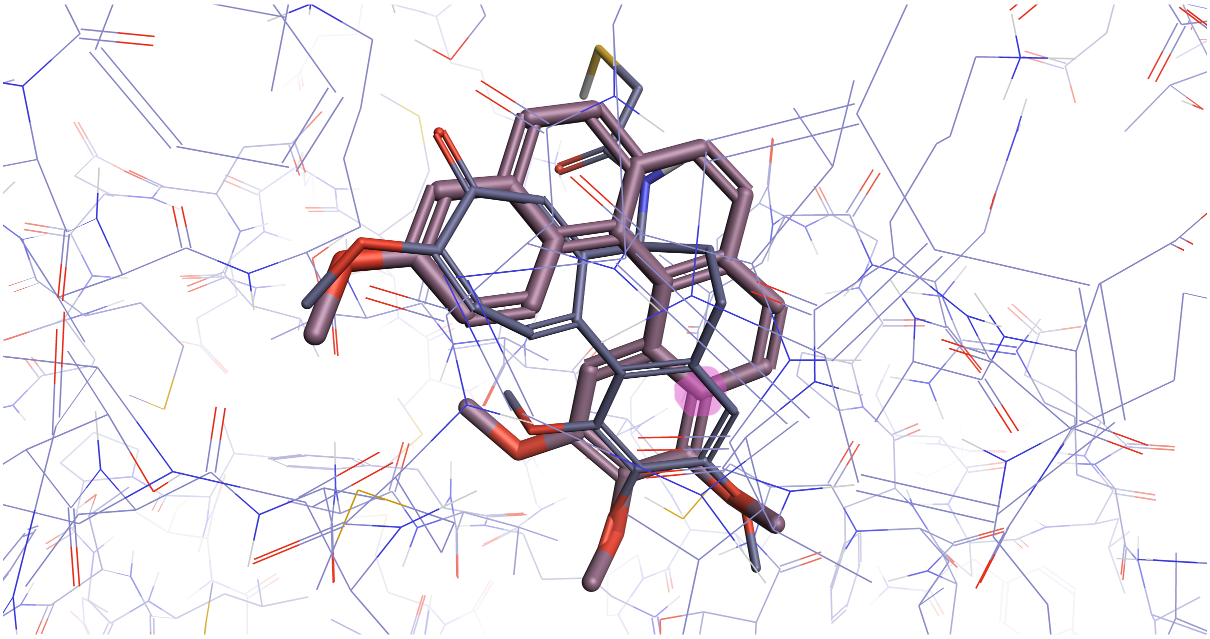

C) CA4

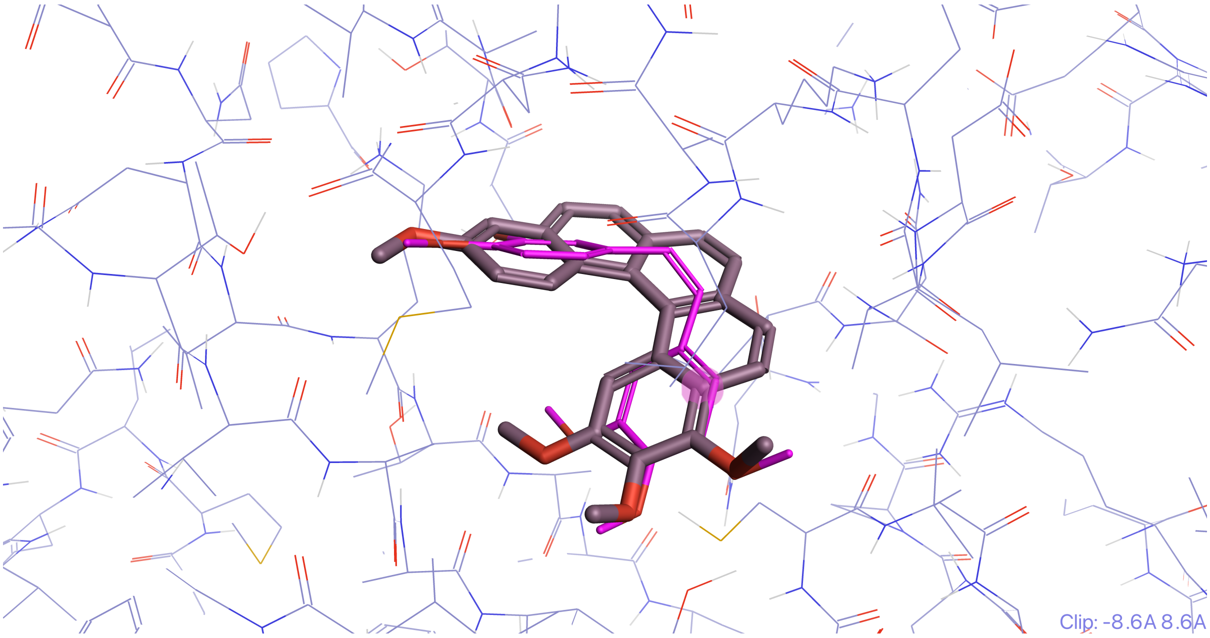

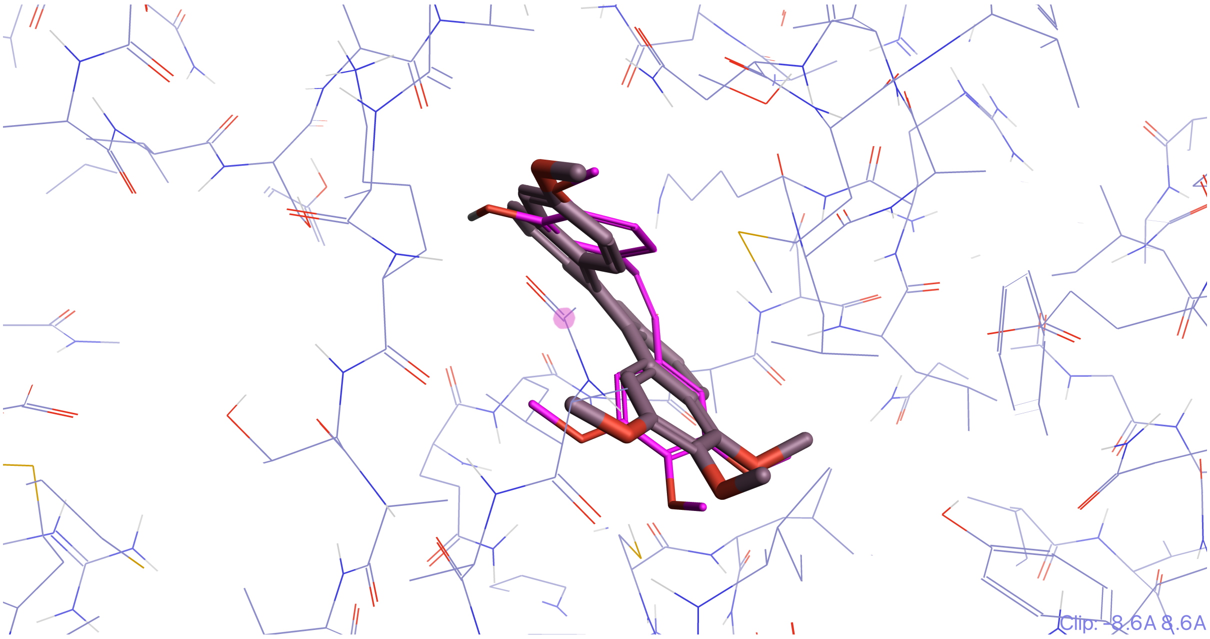

Tubulin-colchicine complex (PDB code: 4O2B)[5]

D) Colchicine

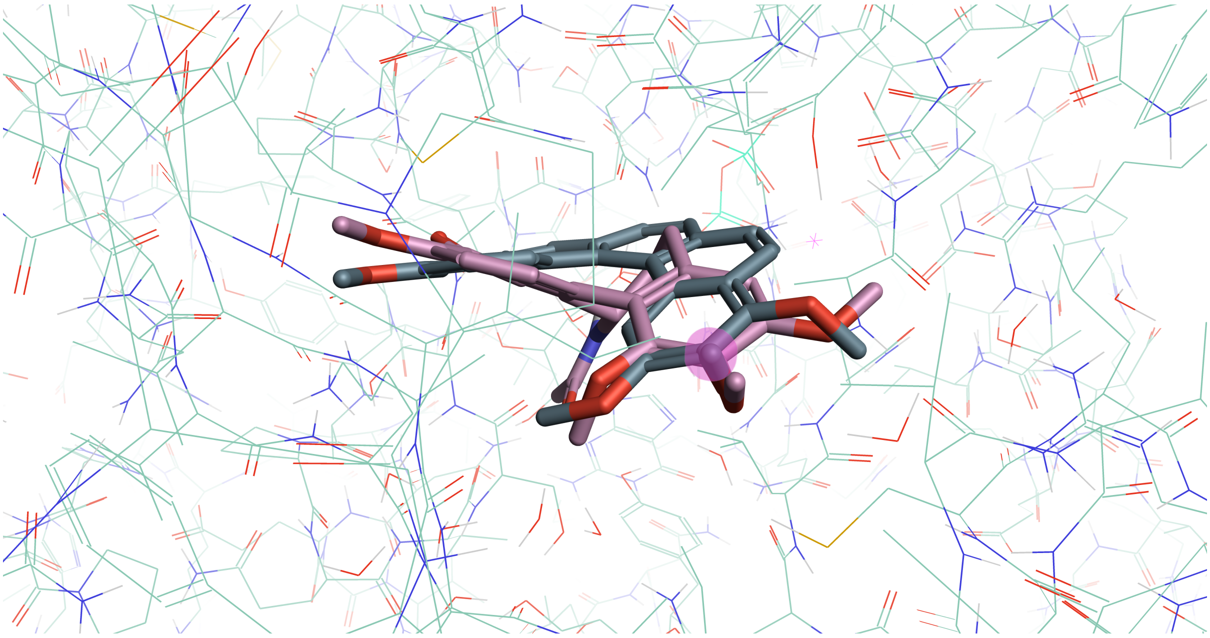

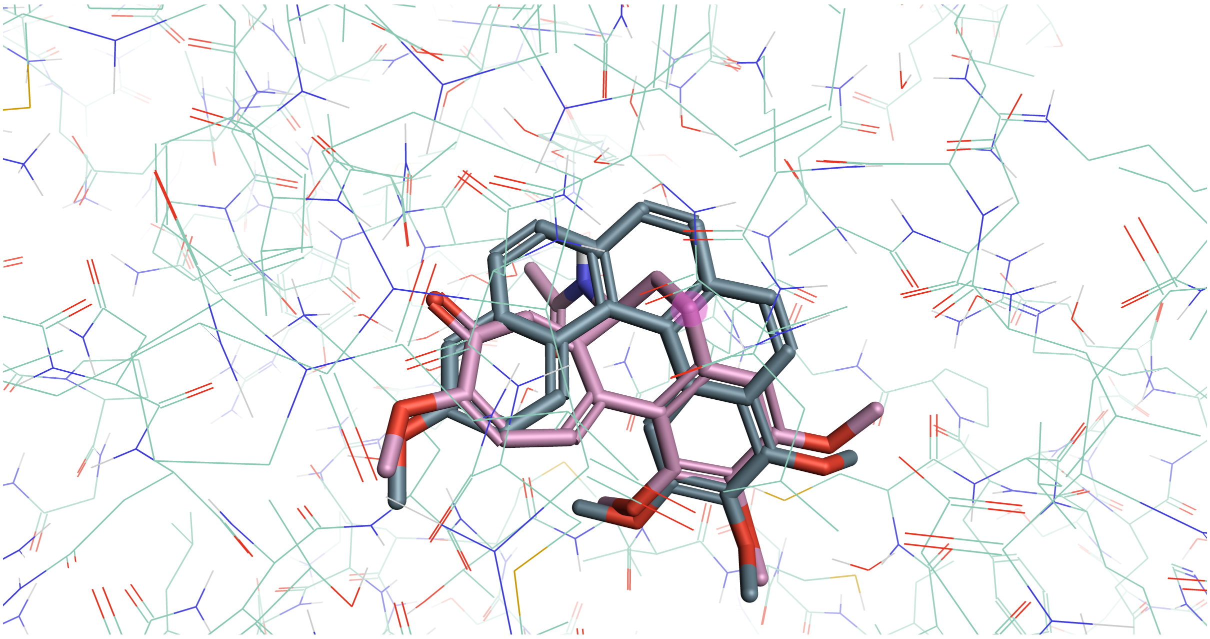

E) CA4

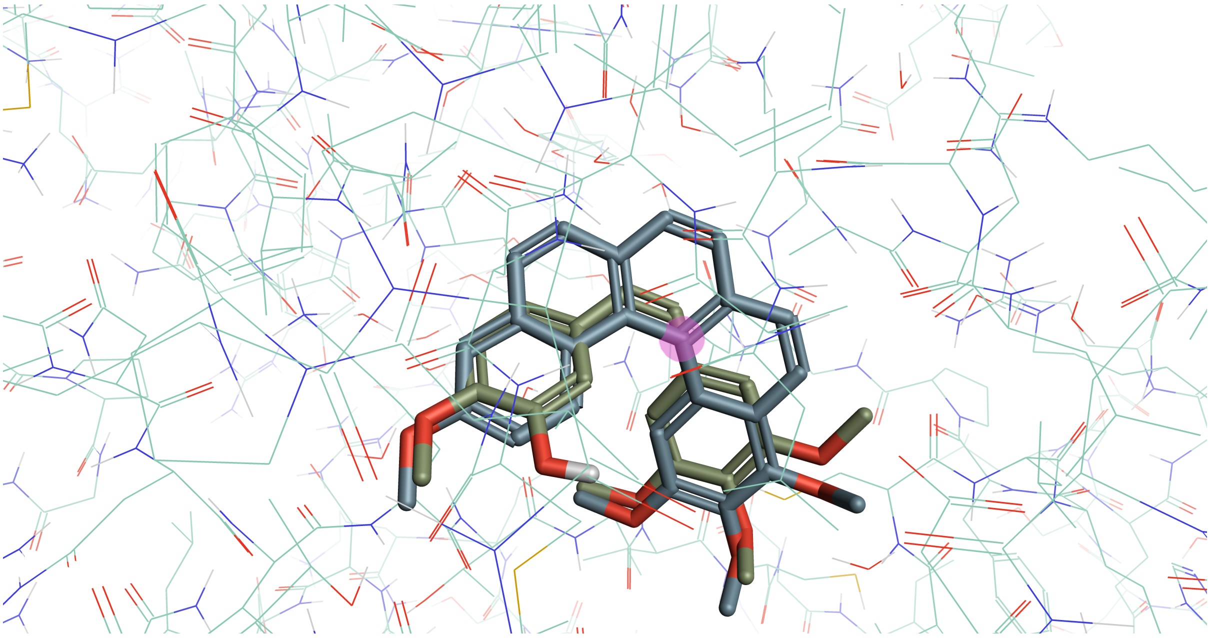

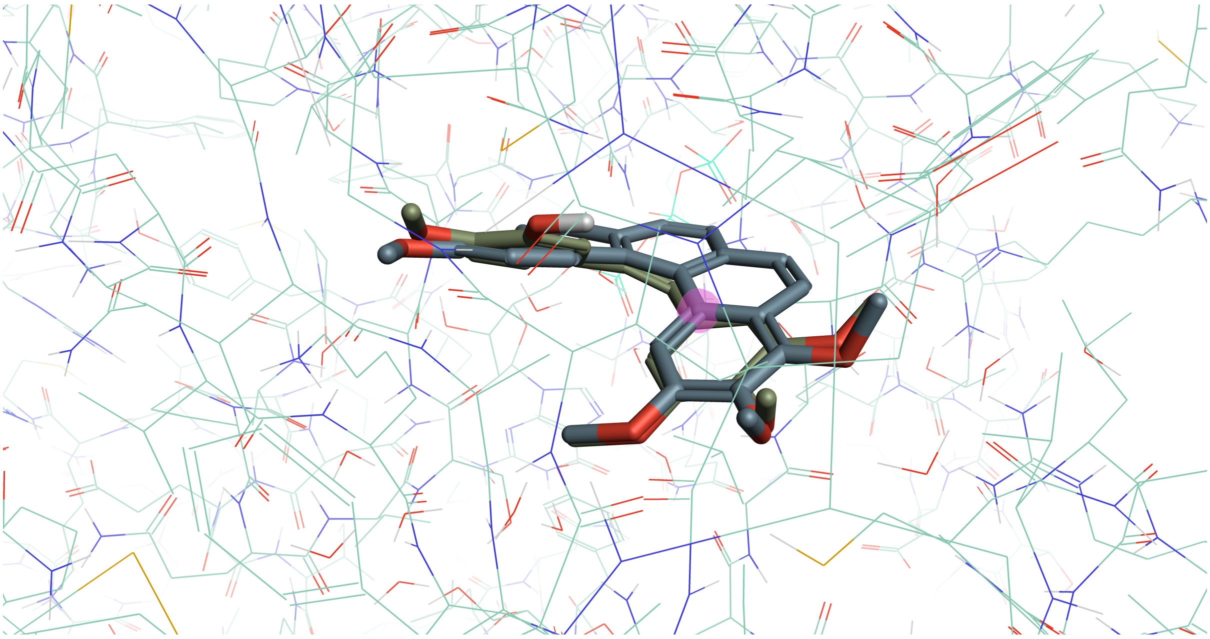

2. Alternative synthetic routes considered

2.1 Oxidative photocyclization

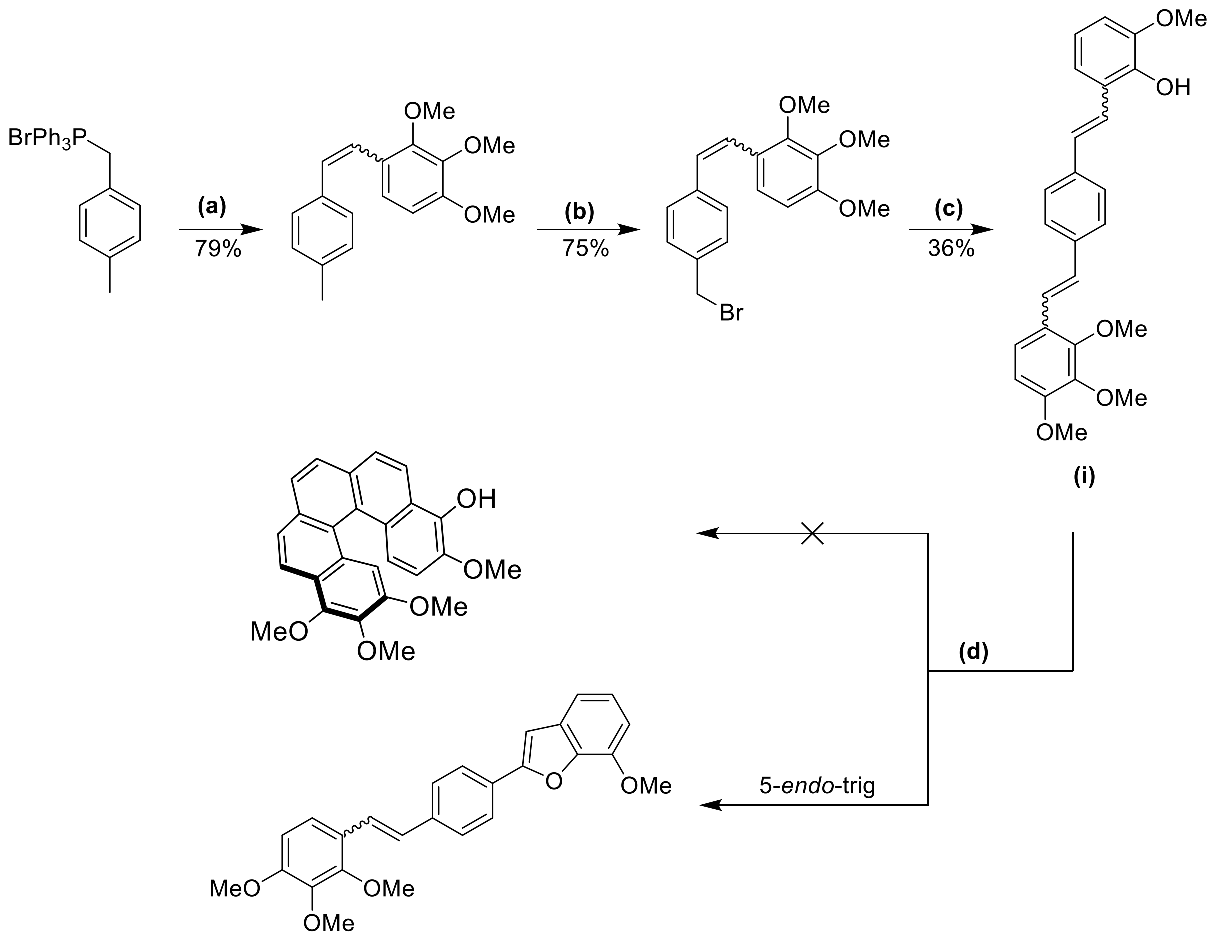

Reaction conditions: **(a)** 2,3,4-trimethoxybenzaldehyde, NaH, THF, 0 °C to rt, 18 h; **(b)** NBS, benzoyl peroxide, benzene, reflux, 4 h; **(c)** **(i)** PPh_3, toluene, reflux, 16 h then, **(ii)** *t*-BuLi, *o*-vanillin, THF, 0 °C, 2 h; **(d)** I_2 (2 equiv.), propylene oxide, benzene, *hv*, rt.

Scheme S1 – Synthesis of phenol-functionalized helistatin was initially attempted *via* oxidative photocyclization methodology, based on conditions first reported by Liu and co-workers.[6] In the final photoisomerization step, MS and NMR analysis showed the formation of the furan, presumably *via* a 5-*endo*-trig cyclisation mediated by radical abstraction of the phenol. The bis-stilbene **(i)** was MOM-protected, however, only decomposition products were observed. The cause of this was believed to be a result of direct competition between the photoisomerization process and fluorescent relaxation of the molecule back to the ground state. Further attempts at forming this linkage in flow were also unsuccessful.

2.2 Oxidative cyclization

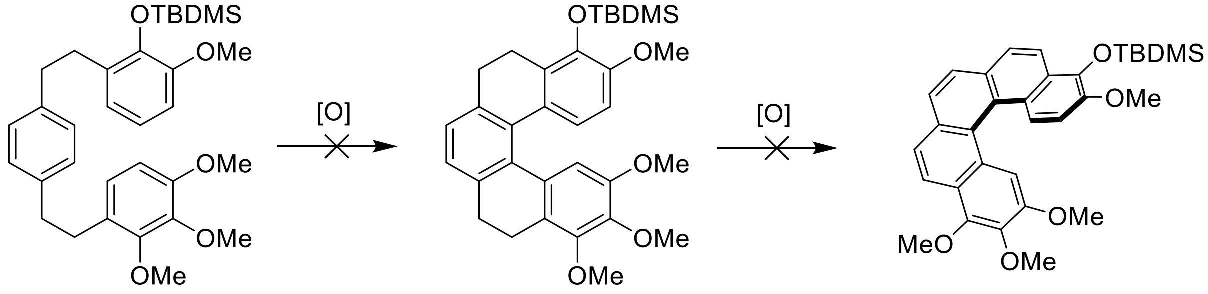

Entry	Reagent	Solvent	Temperature (°C)	Result
**1**	PIDA, BF_3	DCM	-40	Decomposition
**2**	PIDA, BF_3	DCM	-40	Decomposition
**3**	PIDA, BF_3	DCM	-78	Decomposition
**4**	PIDA, BF_3	DCM	-78	Decomposition
**5**	$FeCl_3$	MeCN	-40 to -20	No product, multiple side products
**6**	$Fe(ClO_4)_3$	MeCN	-40 to -20	No product, multiple side products
**7**	PIDA	$(CF_3)_2CHOH$	0	No reaction
**8**	$VOCl_3$	DCM	-78	No product, multiple side products
**9**	PIFA	$(CF_3)CHOH$	0	Decomposition

Scheme S2 – Attempts at forming phenol-functionalized helistatin *via* oxidative cyclization, based on work reported by Kita and co-workers.[7] This methodology was chosen to circumvent the issues arising from the fluorescent nature of bis-stilbene **(i)** synthesized in **Scheme S1**. We envisaged that the electron-neutral rings would be nucleophilic enough to attack the radical intermediate. However, for bis-stilbene **(i)**, the hypervalent reagents preferentially coordinate to the most electron-rich terminal rings, which leaves the electron neutral central ring to carry out an unfavourable nucleophilic attack. This process leads to multiple side products and decomposition of the starting material. Iron complexes and vanadium oxytrichloride were also tested, although these yielded similar results.[8,9]

2.3 Radical cyclization

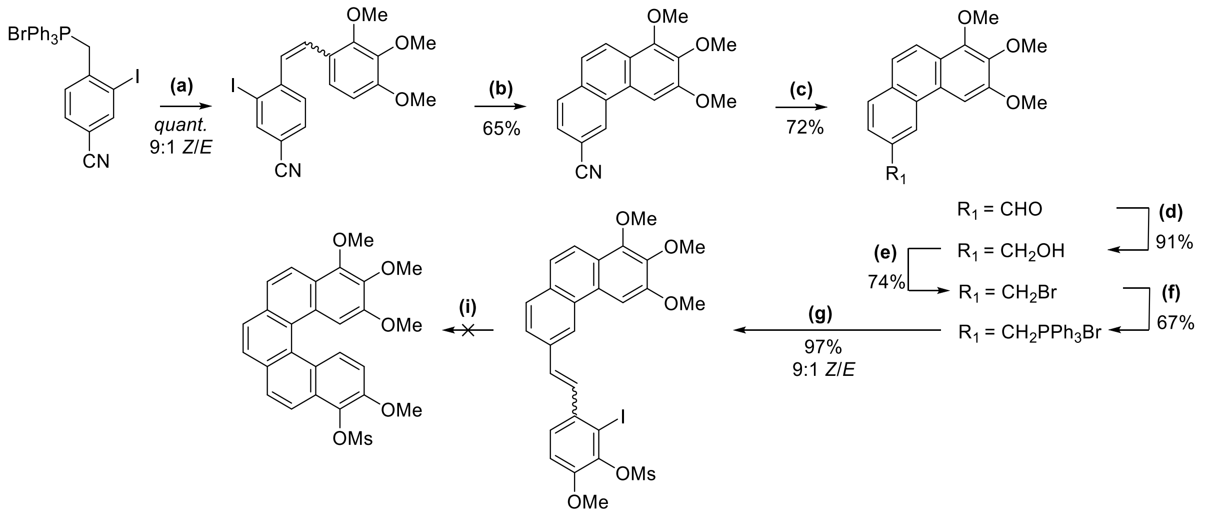

Reaction conditions: **(a)** 2,3,4-trimethoxybenzaldehyde, KO*t*Bu, THF, 0 °C to rt, 16 h; **(b)** Bu_3SnH, AIBN, toluene, 90 °C, 24 h; **(c)** DIBAL-H, THF, 0 °C to rt, 3 h; **(d)** $NaBH_4$, EtOH/1 M NaOH (9:1 v/v), 0 °C, 3 h; **(e)** PBr_3, pyridine, DCM, 0 °C, 3 h; **(f)** PPh_3, toluene, 100 °C, 16 h; **(g)** 3-formyl-2-iodo-6-methoxyphenyl methanesulfonate, KO*t*Bu, THF, 0 °C to rt, 16 h; **(i)** range of conditions tested (see table below).

Entry	Conditions	Solvent	Temperature (°C)	Result
**1**	Bu_3SnH, AIBN	Toluene	90	Clean formation of dehalogenated product
**2**	$Pd(OAc)_2$ (10 mol%), $PCy_3.HBF_4$, (20 mol%) K_2CO_3	DMAc	130	Mixture of dehalogenated product and traces of oxidatively cleaved product
**3**	NHMDS, then standard radical conditions	THF/Toluene	rt/0	Clean formation of dehalogenated product
**4**	$(TMS)_3SiH$	Toluene	90	Clean formation of dehalogenated product
**5**	UV-B	Benzene	rt	Mixture of at least five inseparable products; predominant masses 361 & 297 (indicative of oxidatively cleaved product)
**6**	*fac*-Ir(ppy$_3$), K_3PO_4, DMF, 10 W blue LEDs	DMF	rt	Mixture of starting material and dehalogenated product

Scheme S3 – Synthetic route to phenol-functionalized helistatin *via* radical cyclization, based on methodology reported by Harrowven and co-workers.[10] Unfortunately, the final cyclisation was not possible under a range of conditions and dehalogenation was observed in most cases. This was likely due to the electronic mismatch between the aryl iodide and the electron neutral part of the phenanthrene. Several phenol protecting groups were examined (Bz, Bn, Tos), however, none of these afforded any cyclized product.

2.4 [2+2+2] cycloisomerization

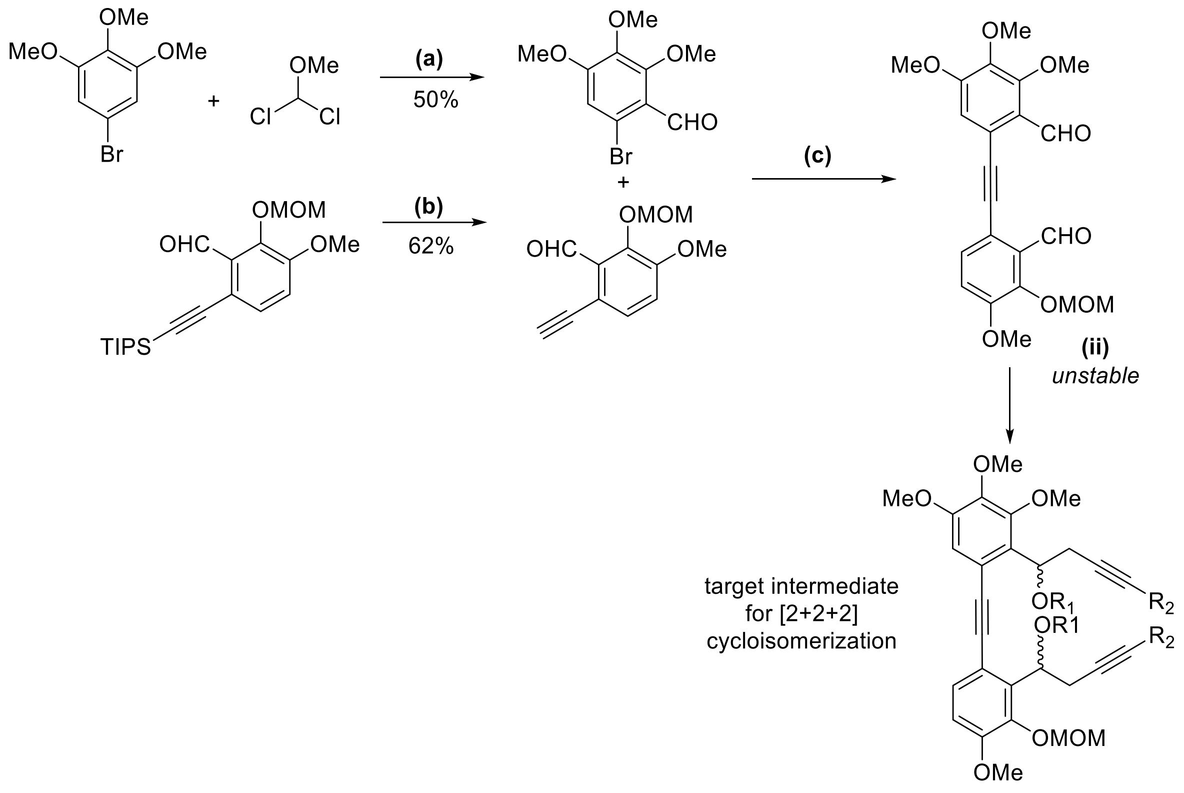

Reaction conditions: **(a)** $TiCl_4$, DCM, 0 °C to rt, 4 h; **(b)** TBAF, THF, rt, 1 h; **(c)** $PdCl_2(PPh_3)_2$, Et_3N, CuI, THF, 50 °C, 16 h.

Scheme S4 – Synthesis of intermediates for the formation of phenol-functionalized helistatin *via* a [2+2+2] cycloisomerization, based on work previously carried out by Songis and co-workers.[11] The coupled product **(ii)** was formed as a minor component in the reaction mixture, however, decomposition occurred during work-up and purification, suggesting that these aldehydes are particularly unstable. π Lewis-acid mediated cyclization of alkynes was tested in parallel and was found to be far more effective, therefore this route was not pursued any further.

3. Synthetic methodology

3.1 General experimental

All reagents and solvents were purchased from commercial sources and used as supplied unless otherwise indicated. All reactions were carried out under an inert atmosphere, using anhydrous solvents. All reactions were monitored by thin-layer chromatography (TLC) using Merck silica gel 60 F254 plates (0.25 mm). TLC plates were visualized using UV light (254 nm) and/or by using an appropriate TLC stain. Silica column chromatography was performed using Merck Silica Gel 60 (230-400 mesh) treated with a solvent system specified in the individual procedures. Solvents were removed by rotary evaporator and the compounds further dried using high vacuum pumps. Infrared spectra were recorded neat on an Agilent Cary 630 FTIR. Reported absorptions are in wavenumbers (cm^{-1}). ^{1}H and ^{13}C NMR were recorded on a Bruker Advance 400 spectrometer at 400 MHz and 100 MHz respectively. Chemical shifts (δ) are quoted in ppm (parts per million) downfield from tetramethylsilane, referenced to residual solvent signals. The following abbreviations are used to designate multiplicity within ^{1}H NMR analysis; s = singlet, d = doublet, t = triplet, q = quartet, m = multiplet, br. = broad signal. High-resolution mass spectra (ESI, APCI) were recorded by the Imperial College London Department of Chemistry Mass Spectroscopy Service using a Micromass Autospec Premier and Micromass LCT Premier spectrometer.

3.2 Synthetic procedures

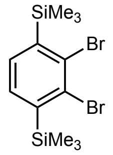

2

To a stirred solution of diisopropylamine (143 mL, 1.02 mol) in THF (1 L) at -78 ºC was added 2.5 M *n*-BuLi in hexanes (390 mL, 0.98 mol) via cannula transfer over the course of 1.5 h. After stirring for an additional 0.5 h, the reaction flask was immersed in an ice-brine bath at -10 ºC and allowed to stir for a further 1 h.* In a separate reaction flask, TMSCl (118 mL, 0.93 mol) is added over 10 min to a stirred solution of 1,2-dibromobenzene (100 g, 0.42 mol) in THF (0.7 L) at -78 ºC. LDA, prepared previously, was introduced via cannula transfer over the course of 1.5 h. The resultant mixture was maintained at -78 ºC for 6 h and allowed to slowly warm to room temperature overnight. Subsequently, the reaction mixture was cooled to 0 ºC using an ice-bath and quenched with NH_4Cl (sat. aq.) and a separate portion of 1 M HCl. The organic phase was separated, and the aqueous phase extracted with Et_2O. The combined organic phases were washed with $NaHCO_3$ (sat. aq.) and brine before being dried over anhydrous $MgSO_4$. Following concentration *in vacuo*, an off-white crystalline solid was obtained.** This material was found to be sufficiently pure for use in the next step without the need for further purification.*** However, a Si plug (100% hexane) can be utilized to remove the small amount of coloured impurities. The title compound was obtained as a crystalline solid (148 g, 93% yield).

TLC R_f = 0.7 (100% hexane)

^{1}H NMR (400 MHz, $CDCl_3$) δ 7.32 (s, 2H), 0.39 (s, 18H).

All other experimental data was found to be in accordance with literature.[12]

*In one instance a white precipitate was seen to form that soon dissolved upon warming the reaction flask to room temperature. **No evidence of the mono-silylated product was detected.

***On a smaller scale, during the course of reaction development, we noted a mixture of mono- and bis-silylated product. Recrystallisation, as reported by Serwatowski and co-workers,[12] afforded pure bis-silylated product with significant loss of yield. However, the mono-silylated product can be efficiently distilled off (1 mbar vacuum pressure) leaving the bis-silylated product behind in the distillation flask. A Si plug (100% hexane) was then utilized to remove the majority of the coloured impurities.

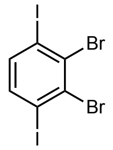

3

A stirred solution of **2** (148 g, 0.39 mol) in CH_2Cl_2 (1 L) was cooled to -10 ºC using an ice-brine bath. ICl (1M in CH_2Cl_2, 800 mL, 0.80 mol) was added over the course of 1 h and the resultant mixture was allowed to slowly warm to room temperature over the course of 18 h. Subsequently, the reaction mixture was cooled to -10 ºC and quenched using a 1:1 solution of $NaHCO_3$ (sat. aq.) and 2 M $Na_2S_2O_4$. Any precipitate that formed was filtered-off through cotton wool and washed with CH_2Cl_2. The organic phase was separated and the aqueous phase extracted with CH_2Cl_2. The combined organic phases were washed with 1 M $Na_2S_2O_4$, $NaHCO_3$ (sat. aq.) and brine before being dried over anhydrous $MgSO_4$. Following concentration *in vacuo*, an oil was obtained that crystalized upon standing. An amount of Cu turnings (~20% by weight of the crude) were activated by stirring in 6 M HCl for 5 minutes, filtered and washed with copious H_2O, MeOH and finally CH_2Cl_2. The resultant activated Cu was added to the previously obtained crude product dissolved in CH_2Cl_2 (~500 mL) with rapid stirring. Both the solution and Cu turnings turned black. After 18 h, the solution was filtered through celite and an aliquot of the filtrate (c.a. 10 mL) was exposed to 2-3 pieces of clean and freshly activated Cu. If the aliquot remained colourless after ~0.5 h, the bulk solution was concentrated *in vacuo* and re-crystalized from iPrOH to yield white needles (63% yield, 119 g, 0.25 mol).

TLC R_f = 0.6 (100% hexane)

^{1}H NMR (400 MHz, $CDCl_3$) δ 7.47 (s, 2H).

All other experimental data was found to be in accordance with literature.[13]

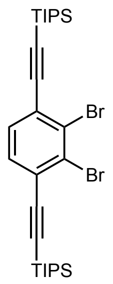

4

To a mixture of **3** (37.5 g, 77.2 mmol), CuI (1.46 g, 7.7 mmol) and $PdCl_2(PPh3)_2$ (2.70 g, 3.8 mmol) was added PhMe (100 mL) followed by (triisopropylsilyl)acetylene (36 mL, 160 mmol) and diisopropylamine (50 mL). The reaction mixture was heated to 70 ºC for 6 h. Subsequently, the reaction was cooled to 0 ºC, diluted with Et_2O and H_2O before the phases were separated. The aqueous layer was extracted with Et_2O and the combined organic layers washed with 1 M HCl, $NaHCO_3$ (sat. aq.), brine and dried over anhydrous $MgSO_4$ before being concentrated *in vacuo*. The crude product was purified using a Si plug (100% hexane) or, alternatively, through re-crystallisation from iPrOH. In both cases, the title compound was obtained as an off-white powder (71% yield, 32.6 g).

TLC R_f = 0.7 (100% hexane)

^{1}H NMR (400 MHz, $CDCl_3$) δ 7.36 (s, 2H), 1.16 – 1.12 (m, 42H).

^{13}C NMR (101 MHz, $CDCl_3$) δ 131.6, 128.9, 127.3, 105.0, 99.2, 18.8, 11.4.

HRMS FTMS+ (*m/z*): [M]+ calculated for $C_{28}H_{45}{}^{79}Br^{81}BrSi_2$: 597.1401; found: 597.1383.

IR (v_{max} / cm^{-1}) 2939, 2863, 2148, 1580.

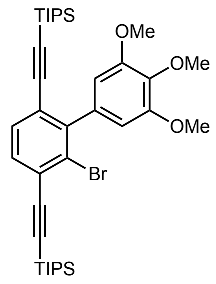

5

To a mixture of **4** (26.4 g, 44.4 mmol), (3,4,5-trimethoxyphenyl)boronic acid (11.3 g, 53.3 mmol), K_2CO_3 (18.4 g, 133 mmol) and Pd(dppf)Cl_2 (3.9 g, 5.33 mmol) was added a degassed mixture of H_2O and 1,2-dimethoxyethane (1:10, 250 mL). The reaction was heated to 85 ºC for 18 h.* Subsequently, the reaction was cooled to room temperature, filtered, diluted with water and the phases separated. The aqueous layer was extracted with Et_2O and the combined organic layers washed with brine and dried over anhydrous $MgSO_4$ before being concentrated *in vacuo*. The crude material was purified by Si column chromatography using a gradient of 5-10% Et_2O in hexane. The title compound was obtained as an orange oil (55% yield, 16.6 g)

TLC R_f = 0.4 (10% Et_2O in hexane)

^{1}H NMR (400 MHz, $CDCl_3$) δ 7.42 (s, 2H), 6.43 (s, 2H), 3.87 (s, 3H), 3.82 (s, 6H), 1.16 – 1.11 (m, 21H), 0.93 (m, 21H).

^{13}C NMR (101 MHz, $CDCl_3$) 153.1, 146.1, 137.5, 136.3, 132.3, 131.0, 127.2, 126.3, 124.7, 106.4, 105.3, 104.9, 98.3, 97.4, 60.9, 56.1, 18.8, 18.6, 11.5, 11.2.

HRMS TOF MS ES+ (*m/z*): [M]+ calculated for $C_{37}H_{56}O_3BrSi_2$: 683.2962; found: 683.2951.

IR (ν_{max} / cm^{-1}) 2939, 2862, 2155, 1582.

*The reaction was observed to stall after 18 h with 25% of starting material left unconverted (NMR analysis of a reaction mixture aliquot). Heating for 24 h did not improve the degree of conversion.

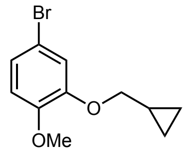

7

Cyclopropymethyl bromide (44 mL, 0.45 mol) was added to a mixture of 4-bromo-2-methoxyphenol (62 g, 0.31 mol) and K_2CO_3 (137 g, 0.99 mol) in THF (500 mL) at 0 ºC. Subsequently, the reaction was warmed to room temperature and heated at 70 ºC for 18 h. Subsequently, the reaction was cooled to room temperature and filtered. 1 M NaOH was added and the phases were separated. The aqueous layer was extracted with Et_2O and the combined organic layers washed with $NaHCO_3$ (sat. aq.), brine and dried over anhydrous $MgSO_4$ before being concentrated *in vacuo*. The title compound was obtained as a light brown crystalline solid (87% yield, 69 g).

TLC R_f = 0.3 (10% Et_2O in hexane)

^{1}H NMR (400 MHz, $CDCl_3$) δ 7.01 (dd, *J* = 8.5, 2.4 Hz, 1H), 6.97 (d, *J* = 2.3 Hz, 1H), 6.73 (d, *J* = 8.5 Hz, 1H), 3.85 (s, 3H), 3.83 (s, 1H), 3.81 (s, 1H), 1.39 – 1.26 (m, 1H), 0.69 – 0.61 (m, 2H), 0.39 – 0.32 (m, 2H).

^{13}C NMR (101 MHz, $CDCl_3$) δ 149.4, 148.9, 123.6, 116.7, 113.0, 112.7, 74.3, 56.2, 10.3, 3.6.

IR (v_{max} / cm^{-1}) 2861, 2995, 2906, 1584, 1497, 1395.

Data in accordance with literature.[14]

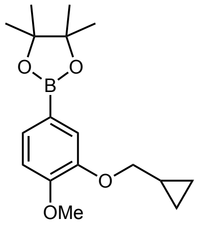

8

To a stirred solution of **7** (25.7 g, 0.10 mol) in THF (500 mL) at -78 ºC was added 2.5 M *n*-BuLi in hexanes (45 mL, 0.11 mol) over the course of 0.5 h. The reaction was stirred for 45 minutes at this temperature before 2-isopropoxy 4,4,5,5-tetramethyl-1,3,2-dioxaborolane (25 mL, 0.12 mol) was added over the course of 20 min. The reaction was allowed to slowly warm to room temperature over the course of 18 h. Subsequently, the reaction was cooled to 0 ºC and quenched with NH_4Cl (sat. aq.) before the phases were separated. The aqueous layer was extracted with Et_2O and the combined organic layers were washed with brine, dried over anhydrous $MgSO_4$ and concentrated *in vacuo*. The crude material was purified by Si column chromatography using 20% Et_2O in hexane. The title compound was obtained as an off-white solid (72% yield, 22.1 g).

TLC R_f = 0.2 (10% Et_2O in hexane)

^{1}H NMR (400 MHz, CD_2Cl_2) δ 7.34 (dd, *J* = 8.0, 1.5 Hz, 1H), 7.19 (d, *J* = 1.4 Hz, 1H), 6.88 (d, *J* = 8.0 Hz, 1H), 3.86 (s, 3H), 3.83 (s, 1H), 3.81 (s, 1H), 1.31 (s, 13H),* 0.68 – 0.59 (m, 2H), 0.34 (dt, *J* = 6.1, 4.6 Hz, 2H).

^{13}C NMR (101 MHz, CD_2Cl_2) δ 152.6, 148.5, 128.9, 118.8, 111.4, 84.1, 74.2, 56.2, 25.2, 10.8, 3.5.

^{11}B NMR (128 MHz, CD_2Cl_2) δ 30.8.

IR (ν_{max} / cm^{-1}) 3077, 2977, 2929, 1597, 1351, 1134.

HRMS APCI ES+ (*m/z*): [M]+ calculated for $C_{17}H_{26}BO_4$: 304.1840; found: 304.1837.

**One proton belonging to the cyclopropyl moiety is obscured/overlaps with the singlet representing the four methyl groups of the Bpin moiety.*

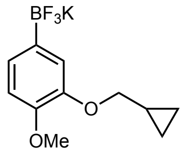

9

The following procedure has been adapted from the literature.[15] To **8** (4.9 g, 16.0 mmol) was added MeOH (32 mL) and MeCN (32 mL) followed by a solution of KF (3.7 g, 63.8 mmol) in H_2O (7 mL). After 5 minutes, a solution of L-(+)-tartaric acid (4.9 g, 32.7 mmol) in warm THF (30 mL) was added drop-wise over the course of 10 minutes. A white precipitate was seen to form. The reaction was stirred for a further 15 minutes and then diluted with MeCN (64 mL). The mixture was filtered and the filtrate concentrated *in vacuo*. Traces of pinacol were removed by heating the crude solid with a heat gun at a vacuum pressure of 10 mbar. The title compound was obtained as a free-flowing white powder (89% yield, 4.0 g).

^{1}H NMR (400 MHz, DMSO-d_6) δ 6.84 – 6.77 (m, 2H), 6.68 (d, J = 7.7 Hz, 1H), 3.70 (s, 1H), 3.68 (d, J = 4.2 Hz, 4H), 1.18 (dddd, J = 13.6, 6.8, 5.1, 2.7 Hz, 1H), 0.57 – 0.50 (m, 2H), 0.34 – 0.24 (m, 2H).

^{13}C NMR (101 MHz, DMSO-d_6) δ 146.9, 123.6, 116.8, 111.1, 72.6, 55.4, 10.6, 3.1.

^{19}F NMR (377 MHz, DMSO-d_6) δ -138.30.

^{11}B NMR (128 MHz, DMSO-d_6) δ 2.5.

HRMS Molecular ion not detected.

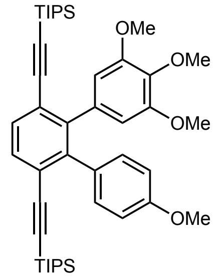

5a

To a mixture of **5** (3.5 g, 7.8 mmol), 4-methoxyphenylboronic acid (1.0 g, 6.7 mmol), K_2CO_3 (2.1 g, 15.4 mmol) and Pd(dppf)Cl_2 (376 mg, 0.51 mmol) was added a degassed mixture of H_2O and 1,2-dimethoxyethane (1:10, 80 mL). The reaction was heated to 85 ºC for 18 h. Subsequently, the reaction was cooled to room temperature, filtered, diluted with water and the phases separated. The aqueous layer was extracted with Et_2O and the combined organic layers washed with brine and dried over anhydrous $MgSO_4$ before being concentrated *in vacuo*. The crude material was purified by Si column chromatography using a gradient of 10-50% Et_2O in hexane. The title compound was obtained as a colourless oil (43% yield, 2.4 g).

TLC R_f = 0.7 (50% Et_2O in hexane)

^{1}H NMR (400 MHz, $CDCl_3$) δ 7.48 (s, 2H), 6.96 (d, *J* = 8.7 Hz, 2H), 6.65 (d, *J* = 8.7 Hz, 2H), 6.27 (s, 2H), 3.75 (s, 3H), 3.71 (s, 3H), 3.64 (s, 6H), 0.95 (s, 21H), 0.93 (s, 21H).

^{13}C NMR (101 MHz, $CDCl_3$) δ 158.4, 152.3, 144.3, 144.1, 136.6, 135.0, 131.7, 131.6, 131.5, 131.3, 124.0, 123.4, 113.0, 107.9, 106.3, 106.3, 96.0, 95.8, 60.9, 56.0, 55.3, 18.7, 18.6, 11.3, 11.3.

HRMS TOF MS ES+ (*m/z*): $[M]^+$ calculated for $C_{44}H_{63}O_4Si_2$: 711.4265; found: 711.4275.

IR (v_{max} / cm^{-1}) 2939, 2862, 2149.

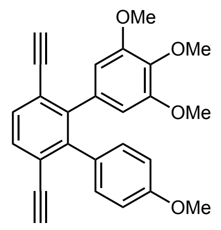

10

To a solution of **5c** (2.4 g, 3.4 mmol) in THF (40 mL) was added 1 M TBAF in THF (7.1 mL, 7.1 mmol). The reaction was stirred at room temperature for 2 h. Subsequently, the reaction was diluted with Et_2O and H_2O before the phases were separated. The aqueous layer was extracted with Et_2O and the combined organic layers washed with brine and dried over anhydrous $MgSO_4$ before being concentrated *in vacuo*. The crude material was purified by Si column chromatography using a gradient of 30-50% Et_2O in hexane. The title compound was obtained as an off-white solid (53% yield, 716 mg).

TLC R_f = 0.4 (50% Et_2O in hexane)

^{1}H NMR (400 MHz, $CDCl_3$) δ 7.54 (s, 2H), 7.00 (d, *J* = 8.7 Hz, 2H), 6.72 (d, *J* = 8.8 Hz, 2H), 6.31 (s, 2H), 3.81 (s, 3H), 3.75 (s, 3H), 3.64 (s, 6H), 3.08 (s, 1H), 3.04 (s, 1H).

^{13}C NMR (101 MHz, $CDCl_3$) δ 158.6, 152.3, 144.3, 144.1, 137.0, 133.9, 132.1, 132.1, 131.5, 131.3, 123.2, 122.6, 113.0, 108.5, 83.1, 83.0, 82.1, 61.0, 56.1, 55.3.

HRMS TOF MS ES+ (*m/z*): [M]+ calculated for $C_{26}H_{23}O_4$: 399.1596; found: 399.1593.

IR (ν_{max} / cm^{-1}) 3276, 2831, 2099.

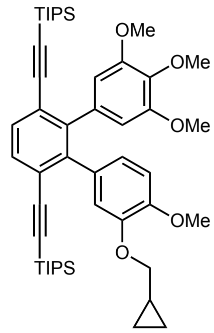

5b

To a mixture of **5** (5.2 g, 7.8 mmol), **9** (2.9 g, 9.9 mmol), Cs_2CO_3 (7.5 g, 22.9 mmol) and Pd(dppf)Cl_2 (0.56 g, 0.76 mmol) was added a degassed mixture of H_2O and 1,2-dimethoxyethane (1:10, 100 mL). The reaction was heated to 85 ºC for 18 h. Subsequently, the reaction was cooled to room temperature, filtered, diluted with water and the phases separated. The aqueous layer was extracted with Et_2O and the combined organic layers washed with brine and dried over anhydrous $MgSO_4$ before being concentrated *in vacuo*. The crude material was purified by Si column chromatography using a gradient of 10-50% Et_2O in hexane. The title compound was obtained as a light yellow oil (65% yield, 4.0 g).

TLC R_f = 0.4 (50% Et_2O in hexane)

^{1}H NMR (400 MHz, $CDCl_3$) δ 7.48 (d, *J* = 1.2 Hz, 2H), 6.73 (dd, *J* = 8.3, 1.9 Hz, 1H), 6.66 (d, *J* = 8.3 Hz, 1H), 6.50 (d, *J* = 1.9 Hz, 1H), 3.78 (s, 3H), 3.74 (s, 3H), 3.63 (s, 6H), 3.55 (s, 1H), 3.53 (s, 1H), 1.19 – 1.10 (m, 1H), 0.93 (d, *J* = 3.0 Hz, 42H), 0.59 – 0.50 (m, 2H), 0.27 – 0.17 (m, 2H).

^{13}C NMR (101 MHz, $CDCl_3$) δ 152.5, 148.5, 147.7, 144.3, 144.0, 136.8, 135.1, 131.9, 131.7, 131.5, 123.9, 123.4, 123.2, 115.8, 111.0, 107.9, 106.3, 106.2, 96.0 95.9, 74.1, 60.8, 56.1, 56.0, 18.6, 11.3, 10.3, 3.4.

HRMS APCI ES+ (*m/z*): [M]+ calculated for $C_{48}H_{69}O_5Si^{2+}$: 781.4678; found: 781.4664.

IR (v_{max} / cm^{-1}) 2939, 2862, 2148, 1582, 1457.

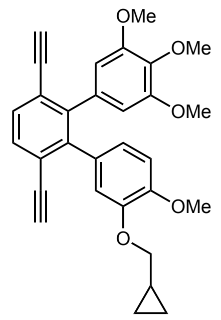

11

To a solution of **5a** (4.0 g, 5.1 mmol) in THF (70 mL) was added 1 M TBAF in THF (10.8 mL, 10.8 mmol). The reaction was stirred at room temperature for 2 h. Subsequently, the reaction was diluted with Et_2O and H_2O before the phases were separated. The aqueous layer was extracted with Et_2O and the combined organic layers washed with brine and dried over anhydrous $MgSO_4$ before being concentrated *in vacuo*. The crude material was purified by Si column chromatography using a gradient of 30-50% Et_2O in hexane. The title compound was obtained as an off-white solid (51% yield, 1.2 g).

TLC R_f = 0.3 (50% Et_2O in hexane)

^{1}H NMR (400 MHz, $CDCl_3$) δ 7.54 (s, 2H), 6.74 (d, *J* = 2.0 Hz, 2H), 6.57 (d, *J* = 1.6 Hz, 1H), 6.33 (s, 2H), 3.83 (s, 3H), 3.80 (s, 3H), 3.64 (s, 6H), 3.58 (d, *J* = 7.0 Hz, 2H), 3.08 (s, 1H), 3.05 (s, 1H), 1.21 – 1.10 (m, 1H), 0.60 – 0.51 (m, 2H), 0.30 – 0.20 (m, 2H).

^{13}C NMR (101 MHz, $CDCl_3$) δ 152.4, 148.7, 147.6, 144.2, 144.1, 137.1, 134.1, 132.2, 132.1, 131.2, 123.3, 123.1, 122.7, 116.2, 110.6, 108.3, 83.0, 83.0, 82.2, 82.1, 74.2, 61.0, 56.1, 56.0, 10.3, 3.5, 1.2.

HRMS Molecular ion not detected.

IR (ν_{max} / cm^{-1}) 3278, 3246, 2957, 2933, 1581

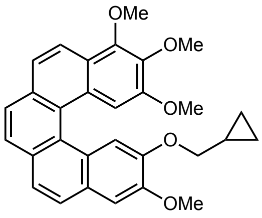

12

$PtCl_2$ (128 mg, 0.48 mmol) was added to a solution of alkyne **11** (562 mg, 1.2 mmol) in degassed PhMe (20 mL). The reaction was heated to 80 ºC for 18 h. Subsequently, the reaction was concentrated *in vacuo* and purified directly via Si column chromatography using a gradient of 30-50% Et_2O in hexane. The title compound was obtained as an orange solid (10% yield, 56 mg).

TLC R_f = 0.3 (40% Et_2O in hexane)

^{1}H NMR (400 MHz, $CDCl_3$) δ 8.22 (d, *J* = 8.7 Hz, 1H), 7.87 – 7.76 (m, 5H), 7.74 (s, 1H), 7.61 (s, 1H), 7.28 (s, 1H), 4.13 (s, 3H), 4.07 (s, 3H), 4.02 (s, 3H), 3.60 (dd, *J* = 10.2, 6.8 Hz, 1H), 3.55 (s, 3H), 3.46 (dd, *J* = 10.2, 7.2 Hz, 1H), 1.19 (d, *J* = 7.1 Hz, 1H), 0.63 – 0.50 (m, 2H), 0.26 – 0.11 (m, 2H).

^{1}H NMR (400 MHz, CD_2Cl_2) δ 8.20 (dd, *J* = 8.7, 0.7 Hz, 1H), 7.87 – 7.79 (m, 5H), 7.66 (s, 1H), 7.55 (s, 1H), 7.31 (s, 1H), 4.11 (s, 3H), 4.04 (s, 3H), 3.98 (s, 3H), 3.52 (s, 3H), 3.51 – 3.39 (m, 2H), 1.20 – 1.13 (m, 1H), 0.57 (dd, *J* = 8.0, 1.7 Hz, 2H), 0.22 – 0.12 (m, 2H).

^{13}C NMR (201 MHz, $CDCl_3$) δ 161.9, 151.1, 149.3, 148.2, 147.2, 140.6, 132.5, 131.6, 128.4, 127.4, 127.3, 126.5, 126.3, 126.2, 125.8, 125.2, 124.9, 124.8, 123.3, 120.8, 111.5, 107.4, 106.6, 73.7, 61.9, 61.3, 56.1, 55.6, 10.2, 3.7, 3.6.

HRMS APCI ES+ (*m/z*): [M]+ calculated for $C_{30}H_{29}O_5^+$: 469.2010; found: 469.2010.

IR (ν_{max} / cm^{-1}) 2953, 2923, 1601.

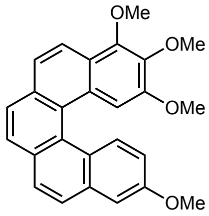

HA-1

Synthesized in an analogous manner to **12**. (7% yield, 30 mg)

TLC R_f = 0.5 (50% Et_2O in hexane)

^{1}H NMR (800 MHz, $CDCl_3$) δ 8.36 (d, *J* = 9.2 Hz, 1H), 8.21 (d, *J* = 8.6 Hz, 1H), 7.90 – 7.83 (m, 2H), 7.83 – 7.76 (m, 3H), 7.67 (s, 1H), 7.29 (s, 1H), 7.00 (dd, *J* = 9.2, 2.6 Hz, 1H), 4.13 (s, 3H), 4.04 (s, 3H), 3.98 (s, 4H), 3.54 (s, 4H).

^{1}H NMR (400 MHz, CD_2Cl_2) δ 8.33 (d, *J* = 9.3 Hz, 1H), 8.20 (d, *J* = 8.7 Hz, 1H), 7.92 – 7.74 (m, 5H), 7.65 (s, 1H), 7.32 (d, *J* = 2.7 Hz, 1H), 7.00 (dd, *J* = 9.2, 2.8 Hz, 1H), 4.11 (s, 3H), 4.00 (s, 3H), 3.97 (s, 3H), 3.52 (s, 3H).

^{13}C NMR (101 MHz, CD_2Cl_2) δ 158.4, 151.9, 148.5, 141.3, 134.9, 132.9, 131.6, 128.2, 127.6, 127.5, 127.4, 127.3, 126.9, 126.2, 125.4, 124.8, 123.6, 121.4, 115.7, 107.6, 106.31, 62.19, 61.5, 55.9, 55.8.

HRMS FTMS+ (*m/z*): [M]+ calculated for $C_{26}H_{23}O_4$: 399.1591; found: 399.1596.

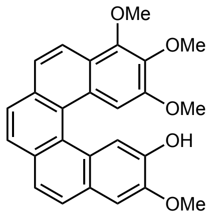

HA-2

A solution of helicene **12** (10 mg, 21.4 μmol) was dissolved in 1:2 MeOH:iPrOH (2 mL) before 12M aqueous HCl (1 mL) was added. The reaction was heated to 60 ºC for 18 h. Subsequently, the reaction mixture was concentrated in concentrated *in vacuo* and subjected to purification by preparative TLC using 4% MeOH in CH_2Cl_2. The title compound was obtained as a purple solid (60% yield, 5.3 mg).

TLC R_f = 0.5 (4% MeOH in CH_2Cl_2)

^{1}H NMR (400 MHz, CD_2Cl_2) δ 8.20 (d, *J* = 8.7 Hz, 1H), 7.94 – 7.73 (m, 6H), 7.68 (s, 1H), 7.32 (s, 1H), 5.83 (s, 1H), 4.12 (s, 3H), 4.08 (s, 3H), 4.00 (s, 3H), 3.58 (s, 3H).

^{13}C NMR (101 MHz, CD_2Cl_2) δ 151.9, 148.6, 147.4, 144.7, 141.4, 132.8, 132.2, 128.8, 127.77, 127.5, 127.1, 127.0, 126.7, 126.5, 126.3, 125.0, 124.9, 123.8, 121.3, 113.5, 107.2, 107.0, 62.2, 61.5, 56.5, 56.0.

HRMS APCI ES- (*m/z*): [M]- calculated for $C_{26}H_{21}O_5^-$: 413.1394; found: 413.1399.

IR (ν_{max} / cm^{-1}) 3425, 3353, 2926, 2852, 1662, 1605.

3.1 Purification of 3 with Cu(0) turnings

As previously noted, samples of **3** were found to be contaminated with elemental sulphur that impacted the subsequent Sonogashira coupling. Visual examination of contaminated batches of **3** revealed the presence of yellow crystals which were assumed to elemental sulphur. Upon treatment with Cu(0) turnings, as detailed above, the resultant material was found to be devoid of such yellow crystals and the Sonogashira coupling step proceeded smoothly. Furthermore, we extracted the yellow crystals from contaminated batches and submitted them for analysis by X-Ray crystallography (see Section 3.3; **3-contaminant**), thus, confirming the presence of sulphur.

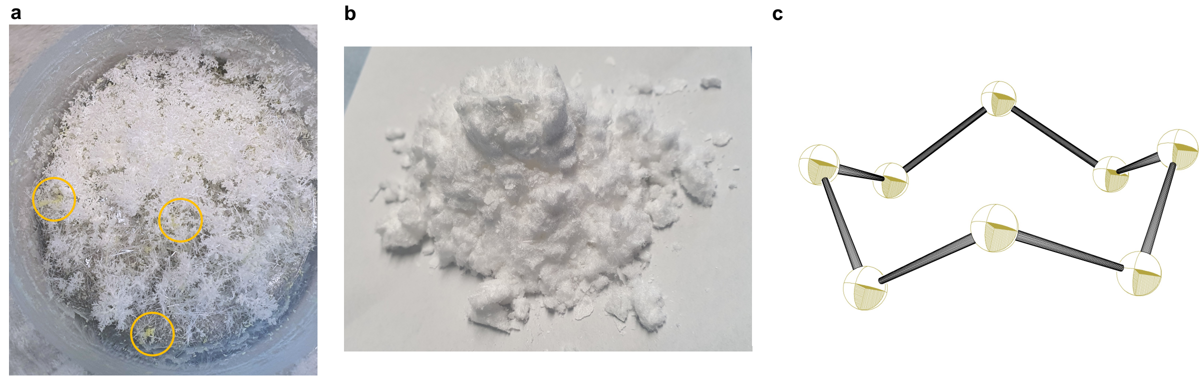

Figure S2 a) A batch of **3** contaminated with visible yellow crystals of S_8 that persist even after re-crystallisation. **b)** The same batch after treatment with Cu(0) turnings. **c)** The crystal structure of the yellow crystals (**3-contaminant**) confirms the presence of S_8.

3.2 Chiral HPLC Purification of HA-1

A racemic sample of **HA-1** was dissolved in neat iPrOH to yield a final sample concentration of ~2.5 mg/mL. Analytical HPLC runs were conducted using an Agilent 1260 LC equipped with a CHIRALPAK-IA column (5 um particle size, column size: 0.46 cm I.D. x 25 cm L; column no. IA00CE-RA035; column temperature: 30 ºC; flow rate: 1 mL/min; injection volume: 1 µL; solvent: n-Hexane/Isopropanol = 95:5; pressure: 42 bar). The chromatogram seen below was thus obtained. The peaks at 8.0 min and 24.6 min represent *(P)*-**HA-1** and *(M)*-**HA-1**, respectively. The absorption spectra of these two peaks is shown overleaf.

Preparative chiral separation of **HA-1** enantiomers was achieved using an Agilent 1260 LC equipped with an auto-collector and a CHIRALPAK-IA column (5 um particle size, column size: 0.46 cm I.D. x 25 cm L; column no. IA00CE-ME018; column temperature: 30 ºC; flow rate: 1 mL/min; injection volume: 50-100 µL; solvent: n-Hexane/Isopropanol = 95:5; pressure: 35 bar). Fractions from multiple runs were collected and submitted for immediate analysis by CD spectroscopy.

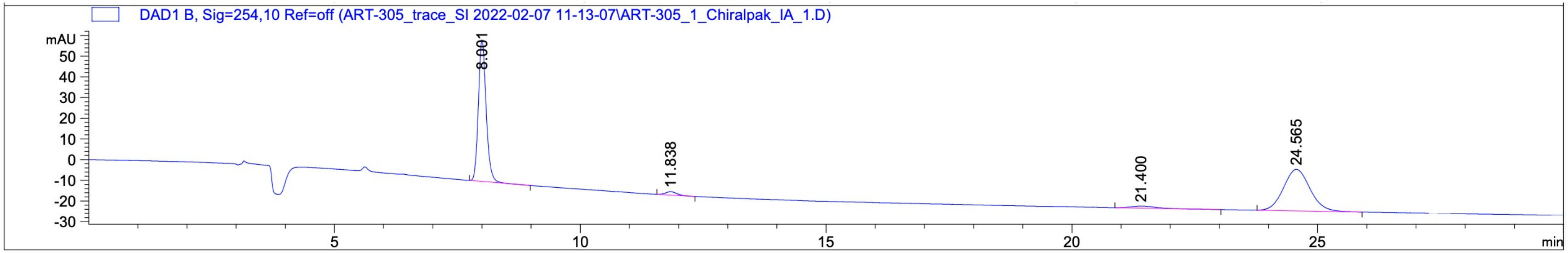

Signal 2: DAD1 B, Sig=254,10 Ref=off

Peak #	RetTime [min]	Type	Width [min]	Area [mAU*s]	Height [mAU]	Area %
1	8.001	BB	0.1683	752.84344	68.44830	47.9669
2	11.838	BB	0.2496	26.78764	1.68360	1.7068
3	21.400	BB	0.4474	37.54837	1.03467	2.3924
4	24.565	BBA	0.5851	752.32782	20.07933	47.9340
Totals :				1569.50728	91.24590	

Figure S3 A chiral HPLC trace of racemic **HA-1**.

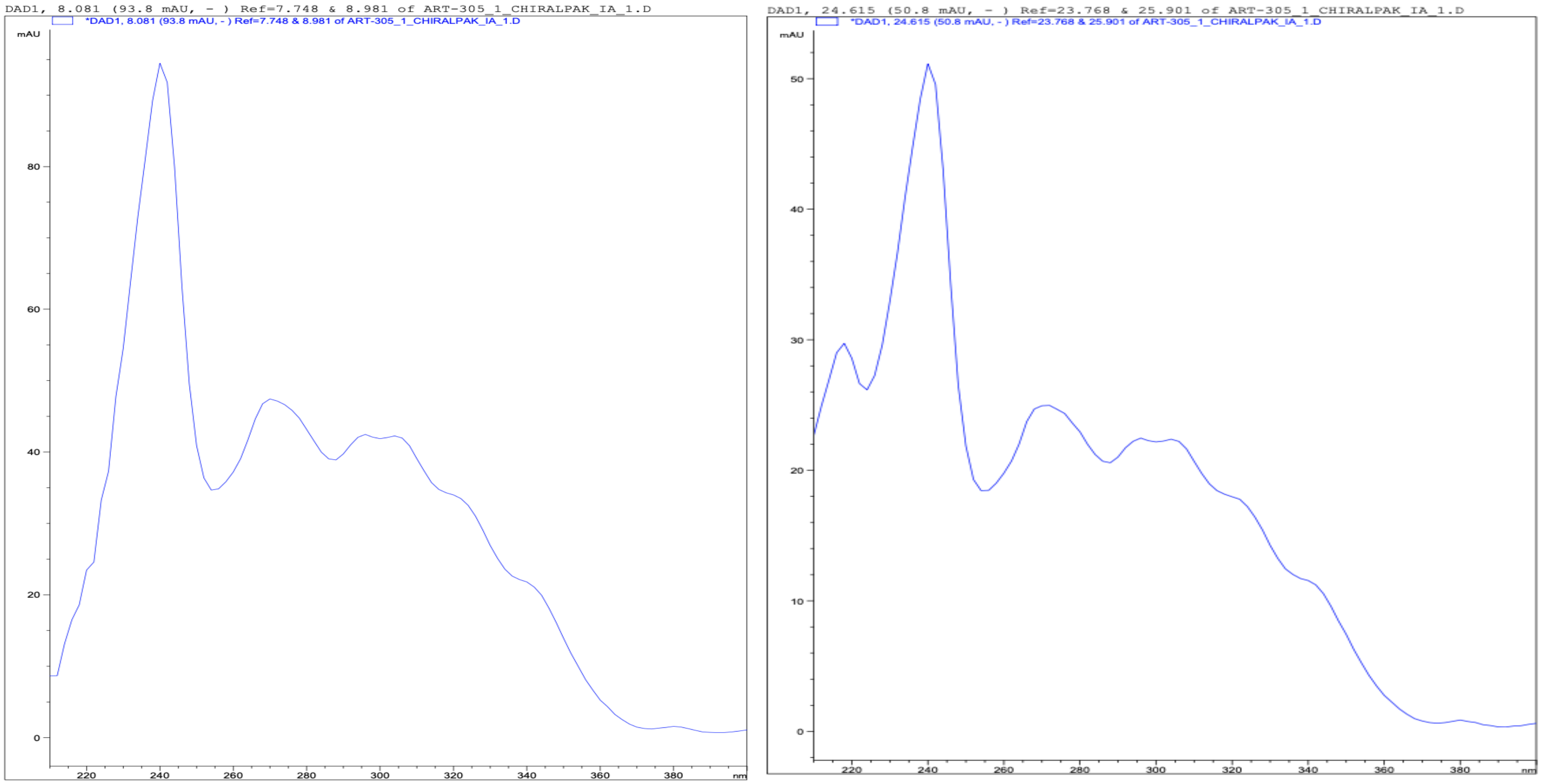

Figure S4 Extracted absorption spectra of the peaks at 8.0 min and 24.6 min, from the above HPLC trace, are shown on the left and right, respectively. The λ_{max} is 240 nm.

3.3 X-Ray Crystallography

The X-ray crystal structure of 2

Crystal data for **2**: $C_{12}H_{20}Br_2Si_2$, *M* = 380.28, monoclinic, $P2_1/n$ (no. 14), *a* = 16.5998(5), *b* = 11.4802(4), *c* = 17.7576(6) Å, β = 95.962(2)°, *V* = 3365.72(18) $Å^3$, *Z* = 8 [2 independent molecules], D_c = 1.501 g cm^{-3}, μ(Mo-Kα) = 4.937 mm^{-1}, *T* = 173 K, colourless tablets, Agilent Xcalibur 3 E diffractometer; 7004 independent measured reflections (R_{int} = 0.0340), F^2 refinement, [16,17] R_1(obs) = 0.0383, wR_2(all) = 0.0732, 4783 independent observed absorption-corrected reflections [$|F_o| > 4\sigma(|F_o|)$, completeness to θ_{full}(25.2°) = 99.7%], 302 parameters. CCDC 2150894. The structure of **2** was found to contain two crystallographically independent molecules (**2-A** and **2-B**) in the asymmetric unit.

The X-ray crystal structure of 3-contaminant

Crystal data for **3-contaminant**: S_8, *M* = 256.48, orthorhombic, *Fddd* (no. 70), *a* = 10.3788(3), *b* = 12.7430(4), *c* = 24.3928(7) Å, *V* = 3226.10(17) $Å^3$, *Z* = 16 [C_2 symmetry], D_c = 2.112 g cm^{-3}, μ(Mo-Kα) = 2.111 mm^{-1}, *T* = 173 K, colourless blocks, Agilent Xcalibur 3 E diffractometer; 824 independent measured reflections (R_{int} = 0.0106), F^2 refinement,[16,17] R_1(obs) = 0.0138, wR_2(all) = 0.0331, 767 independent observed absorption-corrected reflections [$|F_o| > 4\sigma(|F_o|)$, completeness to θ_{full}(25.2°) = 98.9%], 38 parameters. CCDC 2150895. The structure of **3-contaminant**, which is the α polymorph of cyclo-octasulphur, was found to sit across a C_2 axis that bisects the S1–S1A and S4–S4A bonds.

The X-ray crystal structure of 8

Crystal data for **8**: $C_{17}H_{25}BO_4$, *M* = 304.18, monoclinic, $P2_1/c$ (no. 14), *a* = 9.8134(3), *b* = 14.9068(4), *c* = 12.2108(3) Å, β = 94.761(2)°, *V* = 1780.11(8) $Å^3$, *Z* = 4, D_c = 1.135 g cm^{-3}, μ(Mo-Kα) = 0.078 mm^{-1}, *T* = 173 K, colourless blocks, Agilent Xcalibur 3 E diffractometer; 4047 independent measured reflections (R_{int} = 0.0401), F^2 refinement,[16,17] R_1(obs) = 0.0511, wR_2(all) = 0.1246, 2986 independent observed absorption-corrected reflections [$|F_o| > 4\sigma(|F_o|)$, completeness to θ_{full}(25.2°) = 99.9%], 234 parameters. CCDC 2150896. The B14-based 4,4,5,5-tetramethyl-1,3,2-dioxaborolane moiety in the structure of **8** was found to be disordered. Two orientations were identified of *ca*. 67 and 33% occupancy (with a common boron position), their geometries were optimized, the thermal parameters of adjacent atoms were restrained to be similar, and only the non-hydrogen atoms of the major occupancy orientation were refined anisotropically (those of the minor occupancy orientation were refined isotropically).

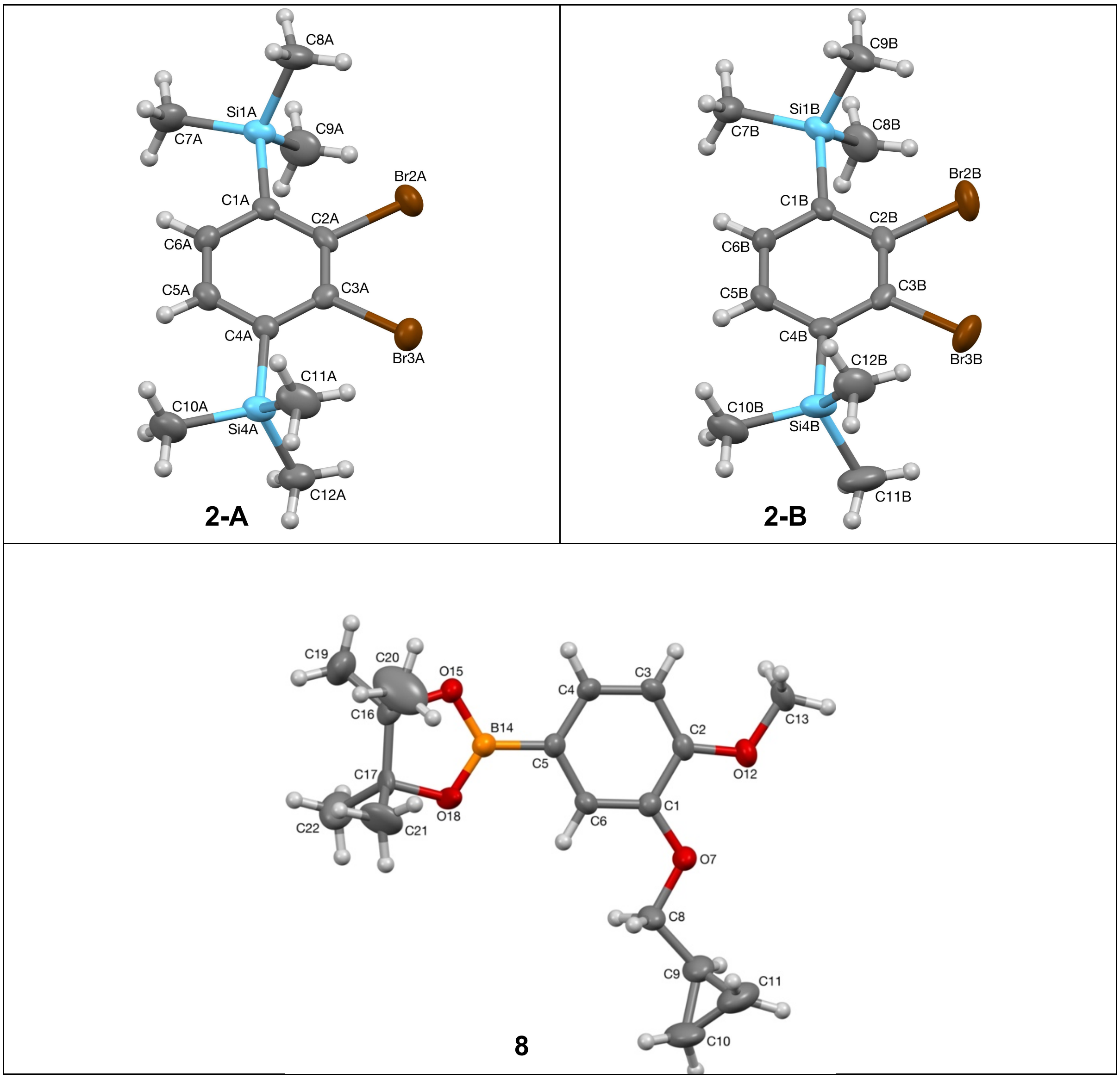

Figure S5 Crystal structures of **2-A, 2-B** and **8** (50% probability ellipsoids).

3.4 Enantiomerisation Kinetics

We examined the enantiomerisation process of **HA-1** at a biologically relevant temperature of 37.5 ºC. CD spectra of (*P*)-**HA-1** and (*M*)-**HA-1** are shown in **Figure S6A**. The enantiomerisation kinetics of (*P*)-**HA-1** was also studied by CD spectroscopy (**Figure S6B**) to obtain a half-life of 1.6 h at 37.5 ºC. For comparison, **pentahelicene** (**Figure S6C**) has an enantiomerisation half-life of 29 h at 25 ºC.[18] Utilising an Eyring plot (see **Table S1** and **Figure S7**), we extrapolated this literature data to obtain a half-life of 6 h at 37.5 ºC for the enantiomerisation of **pentahelicene**.

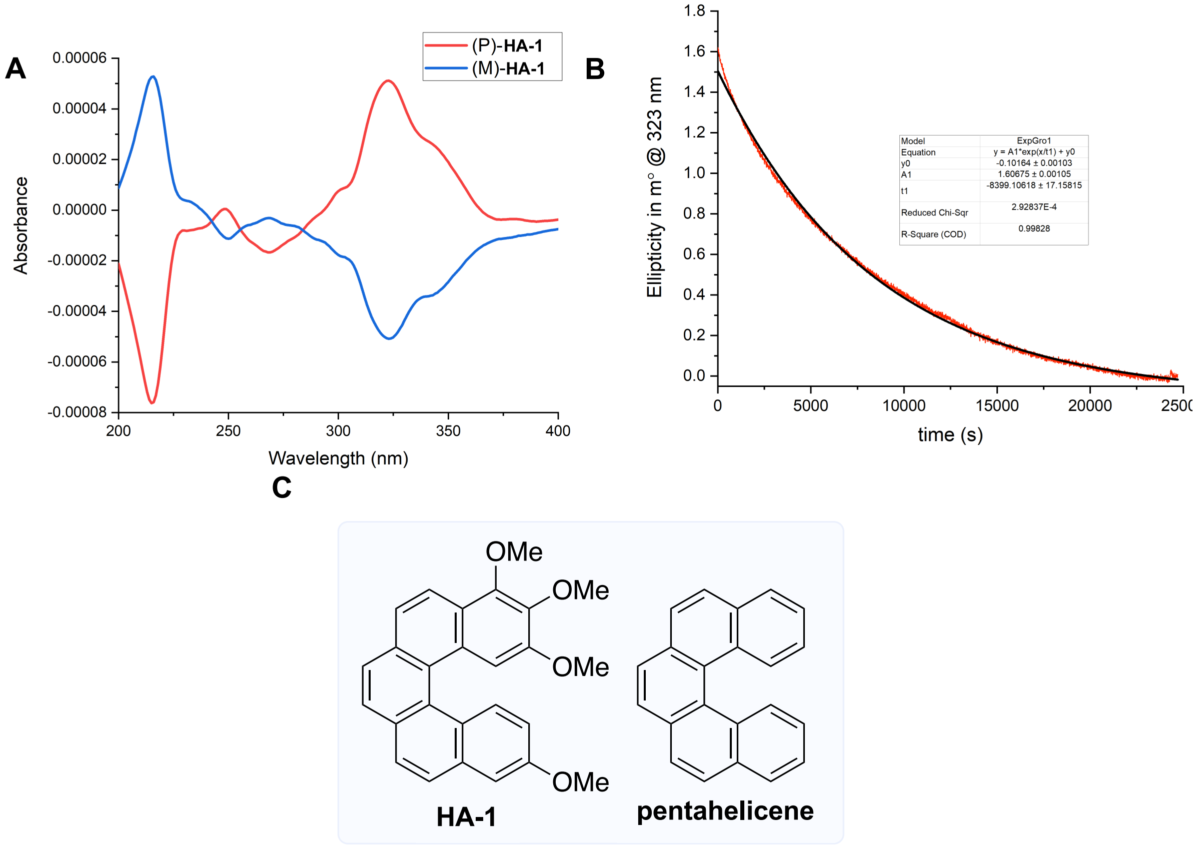

Figure S6 (A) CD spectra of enantioenriched samples of **HA-1**. **(B)** Enantiomerisation kinetics of *(P)*-**HA-1** at 37.5 ºC in 5% iPrOH/Hexane. **(C)** Structures of **HA-1** and pentahelicene.

The activation parameters for **pentahelicene** have been previously reported and are duplicated in **Table S1**.[19] Assuming that enantiomerisation is a first-order single-step process, the thermodynamic parameters can be determined using the Eyring Equation below:

$$\ln\frac{k_e}{T} = \ln\frac{\kappa k_b}{h} + \frac{\Delta S^{\ddagger}}{R} - \frac{\Delta H^{\ddagger}}{R} \times \frac{1}{T}$$

Where k_e is the rate constant of enantiomerisation, T is the temperature, κ is the transmission coefficient which is set to 0.5,[20] k_b is the Boltzmann constant, h is Planck's constant, $\Delta S^{\ddagger}$ is the activation entropy, R is the ideal gas constant and $\Delta H^{\ddagger}$ is the enthalpy of activation. Utilising the parameters in **Table S1**, the Eyring plot in **Figure S7** is obtained.

$\Delta H^{\ddagger}(\mathrm{J \cdot mol^{-1}})$	$\Delta S^{\ddagger}(\mathrm{J \cdot K^{-1}})$	$\Delta G^{\ddagger}(\mathrm{kJ \cdot mol^{-1}})$ at 298K	Enantiomerisation half-life (min) at 298K
190.4	-34.1	100.8	1.74×10^3

Table S1 Activation parameters and enantiomerisation half-life of **pentahelicene**.

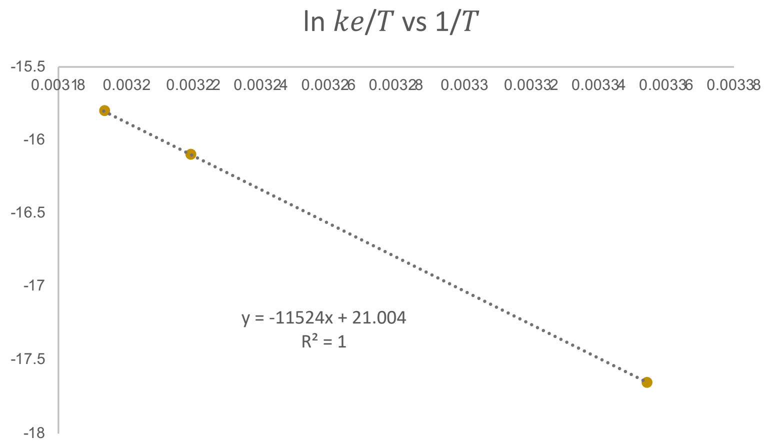

Figure S7 Eyring plot for **pentahelicene**. Highlighted points are at 25 ºC, 37.5 ºC and 40 ºC.

We can then determine the enantiomerisation rate constant (k_e) at 37.5ºC, or any other temperature, and use the following equation to calculate the enantiomerisation half-life:

$$\frac{\ln(2)}{k_e} = \text{enantiomerisation half-life}$$

4. Biological assays

4.1 Resazurin antiproliferation assay

HeLa human cervical cancer cells were sourced from ATCC and maintained under standard cell culture conditions in Dulbecco's modified Eagle's medium (DMEM; PAN-Biotech: P04-035550) supplemented with 10% fetal calf serum (FCS), 100 U/mL penicillin and 100 U/mL streptomycin. Cells were grown and incubated at 37°C in a 5% CO_2 atmosphere. Cells were seeded in 96-well plates at 10,000 cells/well and left to adhere for 24 h, before treating with test compounds for 48 h (final well volume 100 µL, 1% DMSO; three technical replicates); the cosolvent control was treated with DMSO only. Cells were then treated with resazurin for 3 h to measure metabolic activity (through reduction to fluorescent resorufin) as a readout for live cell count.[21] Fluorescence was measured at 590 nm (excitation 544 nm) using a FLUOstar Omega microplate reader (BMG Labtech). Absorbance data was normalized to viable cell count from the cosolvent control cells as 100% viability, where 0% viability was set to correspond to fluorescence signal from PBS with no cells, treated with resazurin (this underestimates true values corresponding to "no live cells" by ca. 5-15%, but does not affect assay outcomes and interpretation). Three independent experiments were performed. Viability data were plotted against the log of compound concentration ($\log_{10}$([drug]) (M)). One representative HeLa experiment out of three is shown as **Figure 3A** (one datapoint per technical replicate). HA-1 shows antiproliferative activity while HA-2 does not.

4.2 Cell-free tubulin polymerisation assay

99% tubulin from porcine brain was obtained from Cytoskeleton Inc. (cat. #T240). Polymerisation was performed at 5 mg/mL tubulin, in polymerisation buffer BRB80 (80 mM piperazine-N,N′-bis(2-ethanesulfonic acid) (PIPES) pH = 6.9; 0.5 mM EGTA; 2 mM $MgCl_2$), in a cuvette (120 µL final volume, 1 cm path length) in a Agilent CaryScan 60 with Peltier cell temperature control unit maintained at 37°C; with glycerol (10 µL). Tubulin was first incubated for 5 min at 37°C with the test compound in buffer with 3% DMSO, without GTP. Then GTP was added (1 µL spike, with mixing, final GTP concentration 1 mM) to initiate polymerisation, and the change in absorbance at 340 nm due to scattering from the turbid medium was monitored (greater turbidity = more polymerisation).

4.3 Cellular microtubule dynamics imaging

HeLa cells were transfected with EB3-GFP using FuGENE 6 (Promega) according to manufacturer's instructions. Experiments were imaged on a Nikon Eclipse Ti microscope

equipped with a perfect focus system (Nikon), a spinning disk-based confocal scanner unit (CSU-X1-A1, Yokogawa), an Evolve 512 EMCCD camera (Photometrics) attached to a 2.0× intermediate lens (Edmund Optics), a Roper Scientific custom-made set with 487 nm (150 mW) laser, ET-GFP filter (Chroma), a motorized stage MS-2000-XYZ, a stage top incubator INUBG2E-ZILCS (Tokai Hit) and lens heating calibrated for incubation at 37°C with 5% CO_2. Microscope image acquisition was controlled using MetaMorph 7.7 and images were acquired using a Plan Apo VC 40× NA 1.3 oil objective. Imaging conditions were initially confirmed to minimize GFP bleaching and phototoxicity in untreated cells. For compound treated acquisitions, compound diluted in prewarmed cell medium was applied to cells and incubated on cells for at least 1 min before commencing acquisition. Comet count analysis was performed in ImageJ using the ComDet plugin (Katrukha E. 2020, ComDet plugin for ImageJ, v0.5.3, Zenodo, doi:10.5281/zenodo.4281064). Blinding was not performed as assay readout is unbiased (Fiji/ImageJ plugins). Data were analysed using Prism 9 software (GraphPad).

5. NMR Spectra

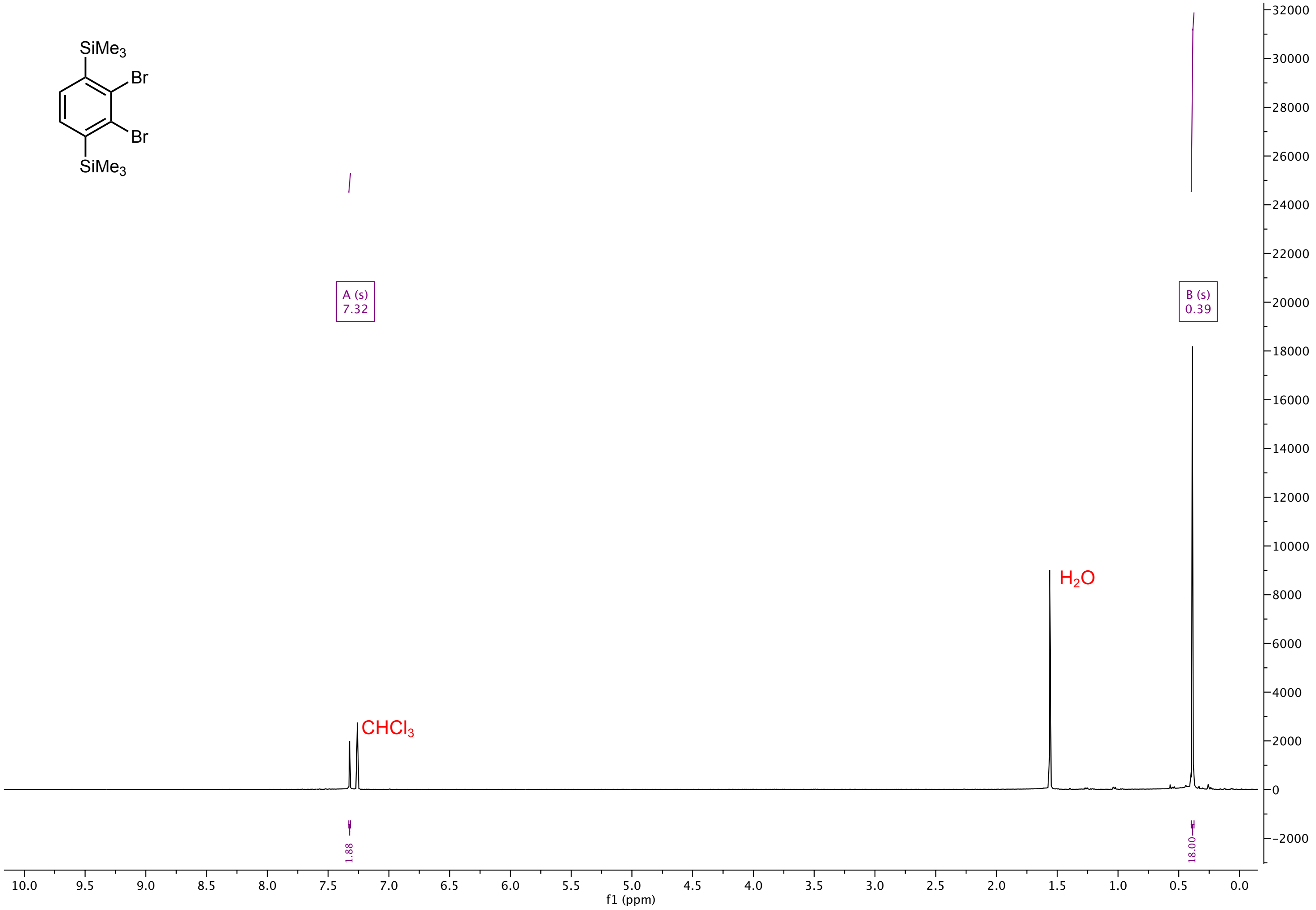

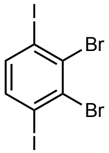

I
Br
Br
I

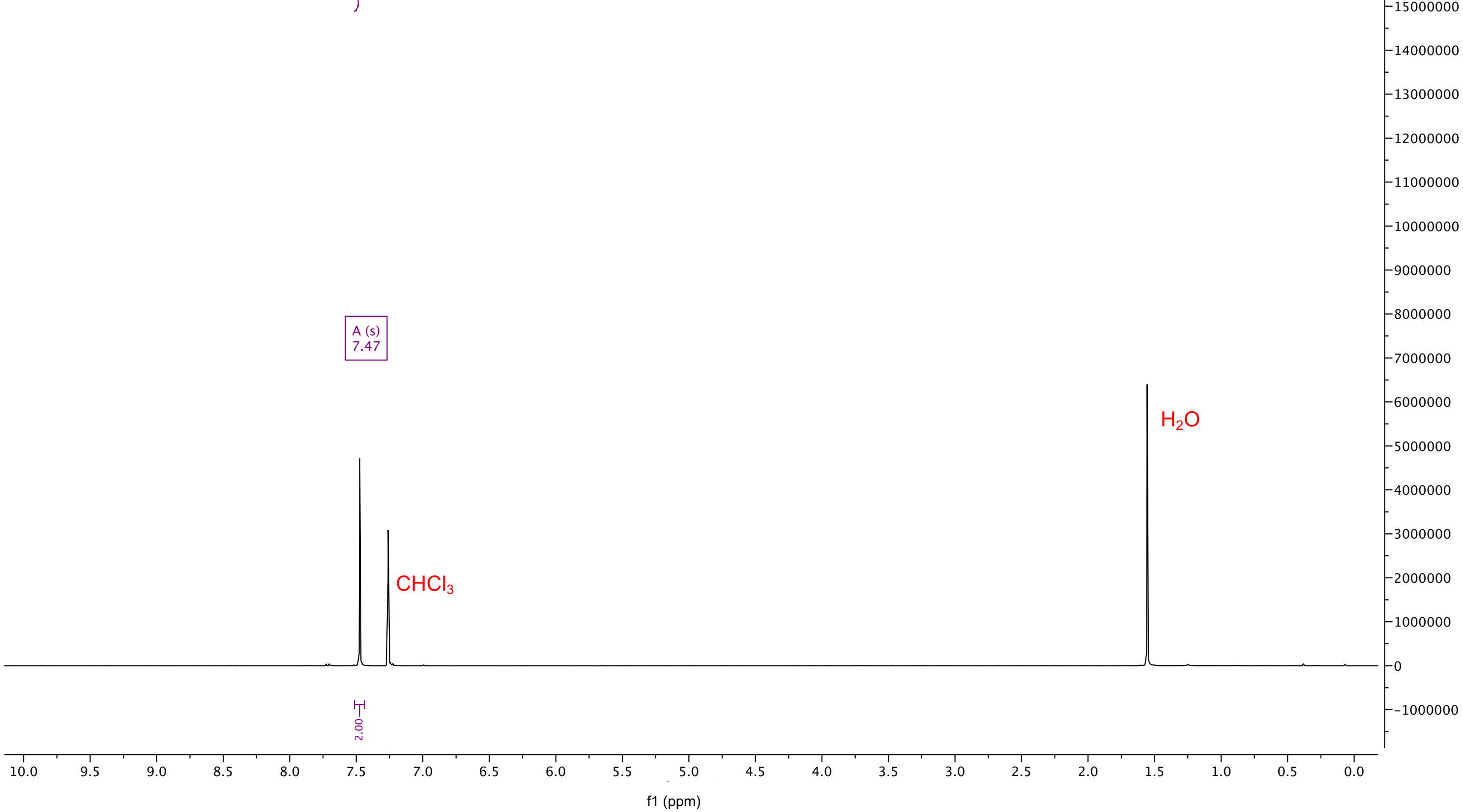

A (s)
7.47
CHCl3
H2O
2.00
20000000
19000000
18000000
17000000
16000000
15000000
14000000
13000000
12000000
11000000
10000000
9000000
8000000
7000000
6000000
5000000
4000000
3000000
2000000
1000000
0
-1000000
10.0
9.5
9.0
8.5
8.0
7.5
7.0
6.5
6.0
5.5
5.0
4.5
4.0
3.5
3.0
2.5
2.0
1.5
1.0
0.5
0.0
f1 (ppm)

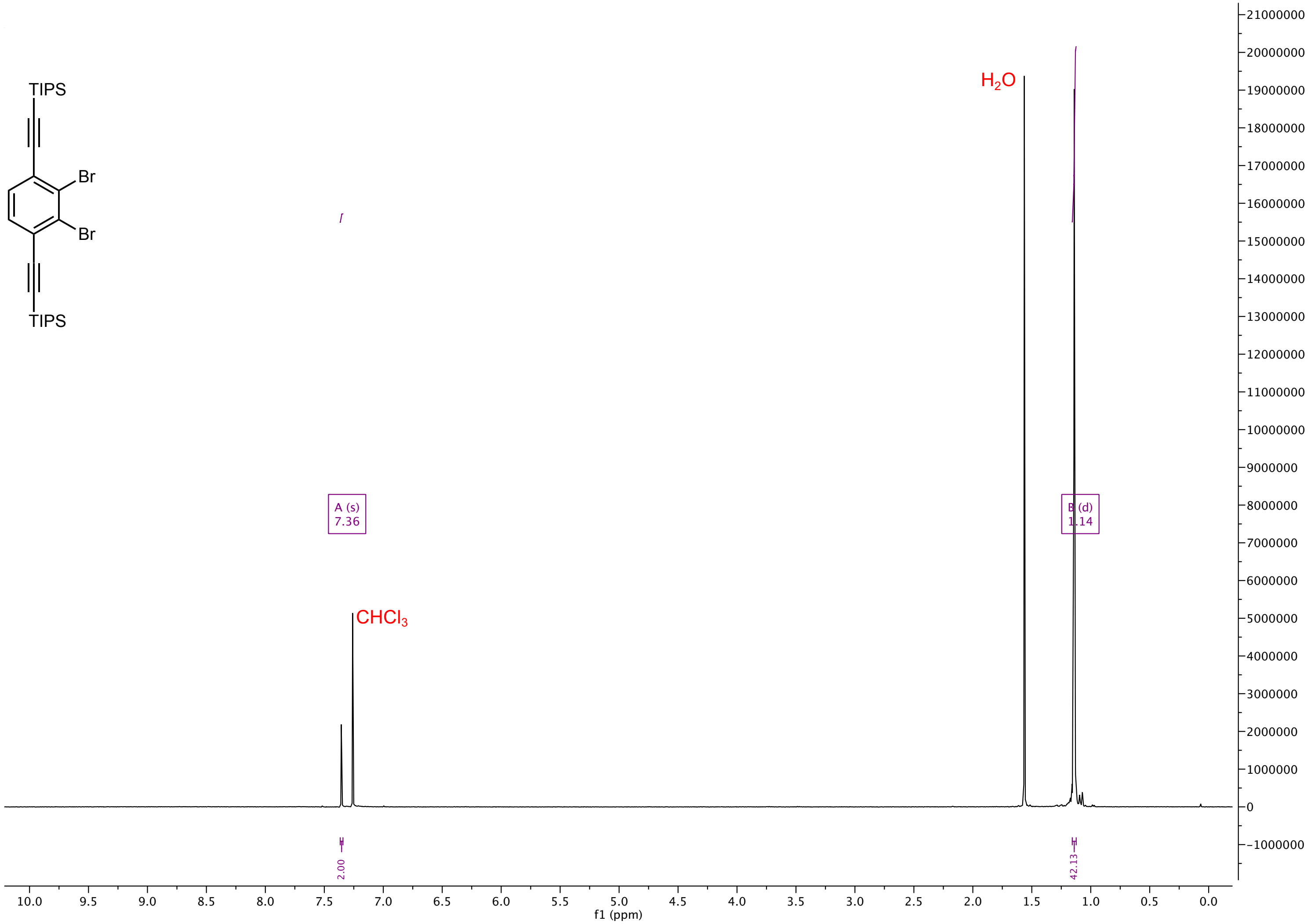

TIPS
Br
Br
TIPS
H2O
CHCl3
A (s)
7.36
B (d)
1.14
2.00
42.13
f1 (ppm)

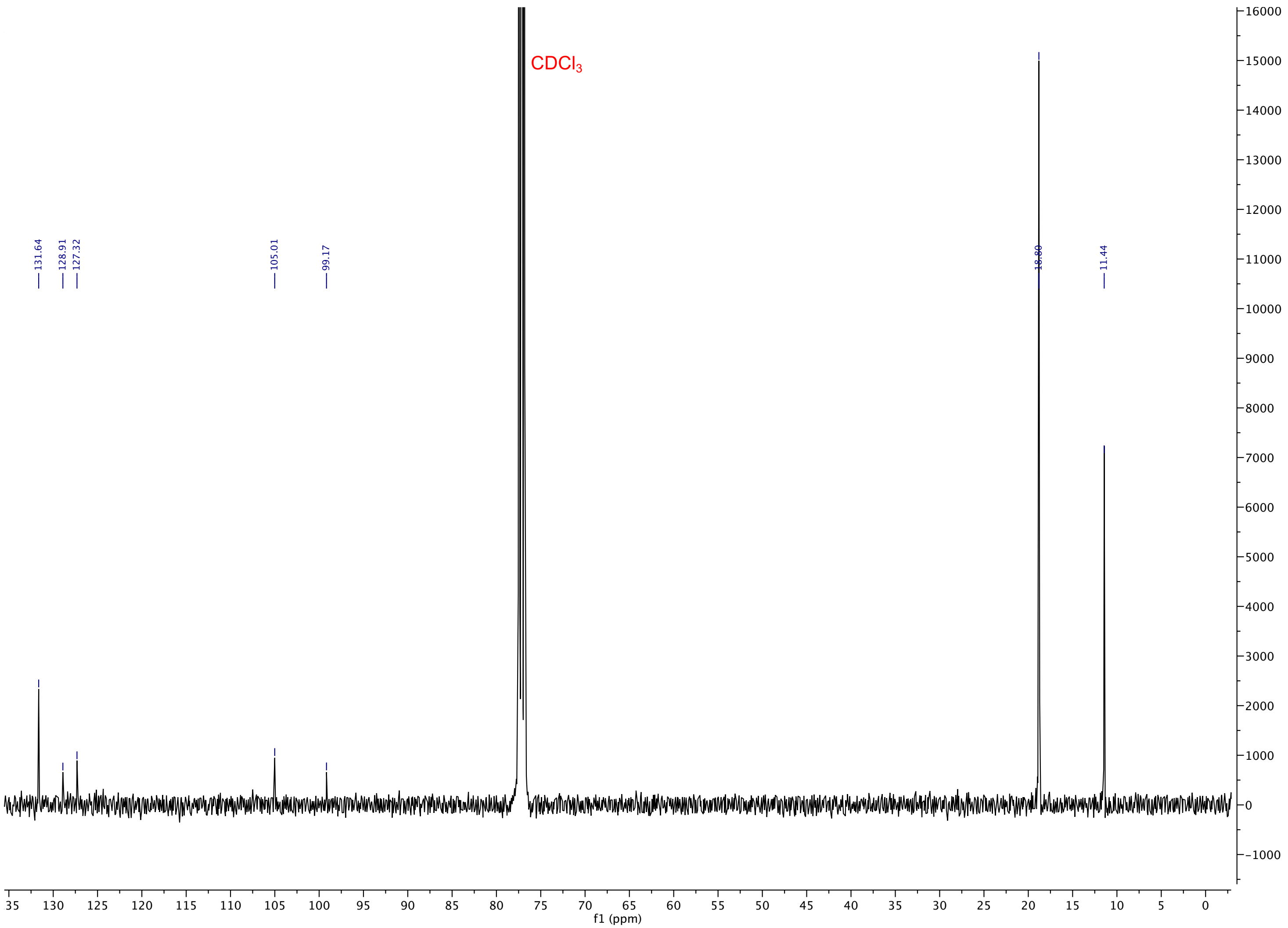
131.64
128.91
127.32
105.01
99.17
18.89
11.44
CDCl$_3$
f1 (ppm)

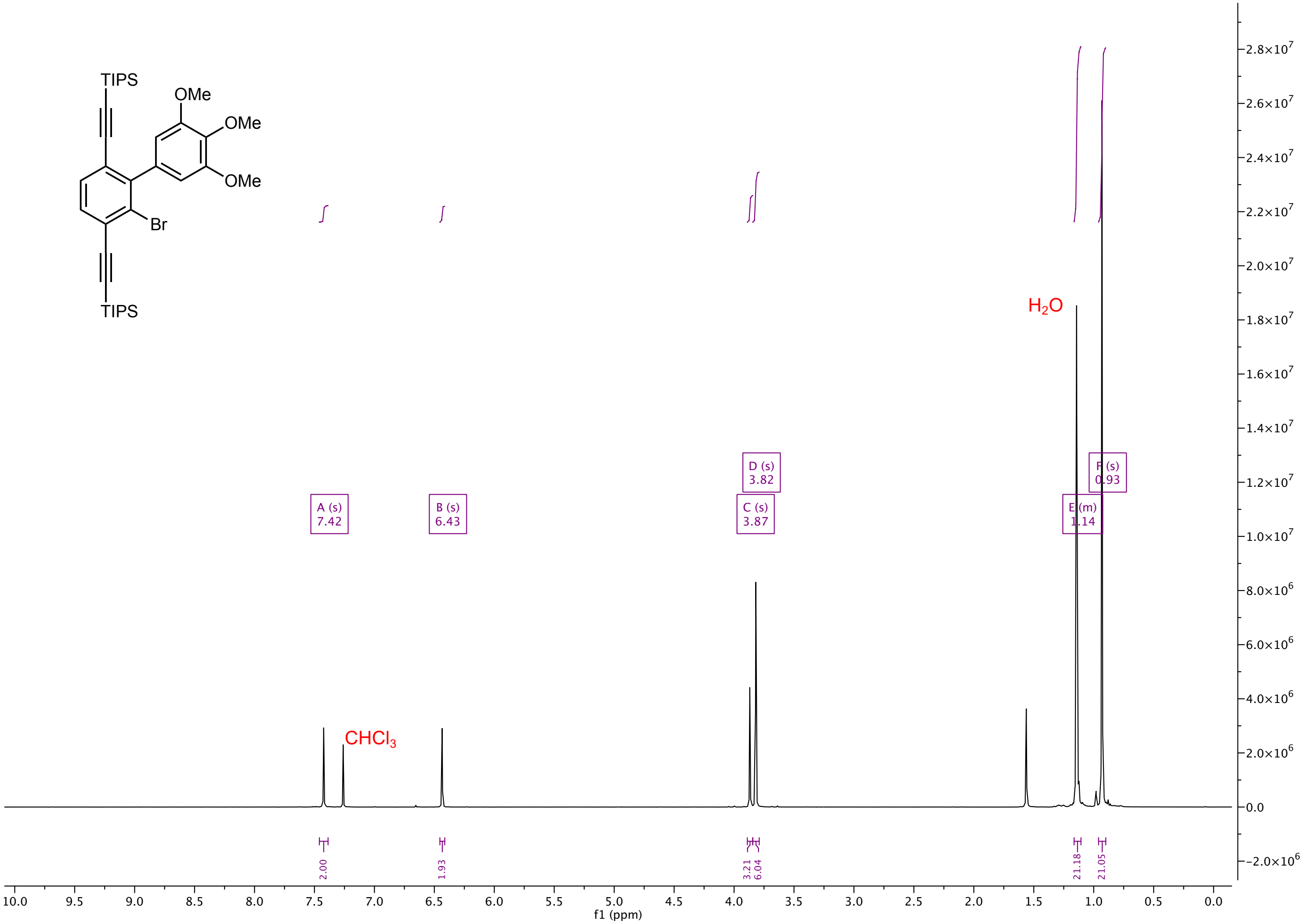
TIPS
OMe
OMe
OMe
Br
TIPS
H2O
CHCl3
A (s)
7.42
B (s)
6.43
C (s)
3.87
D (s)
3.82
E (m)
1.14
F (s)
0.93
2.00
1.93
3.21
6.04
21.18
21.05
f1 (ppm)

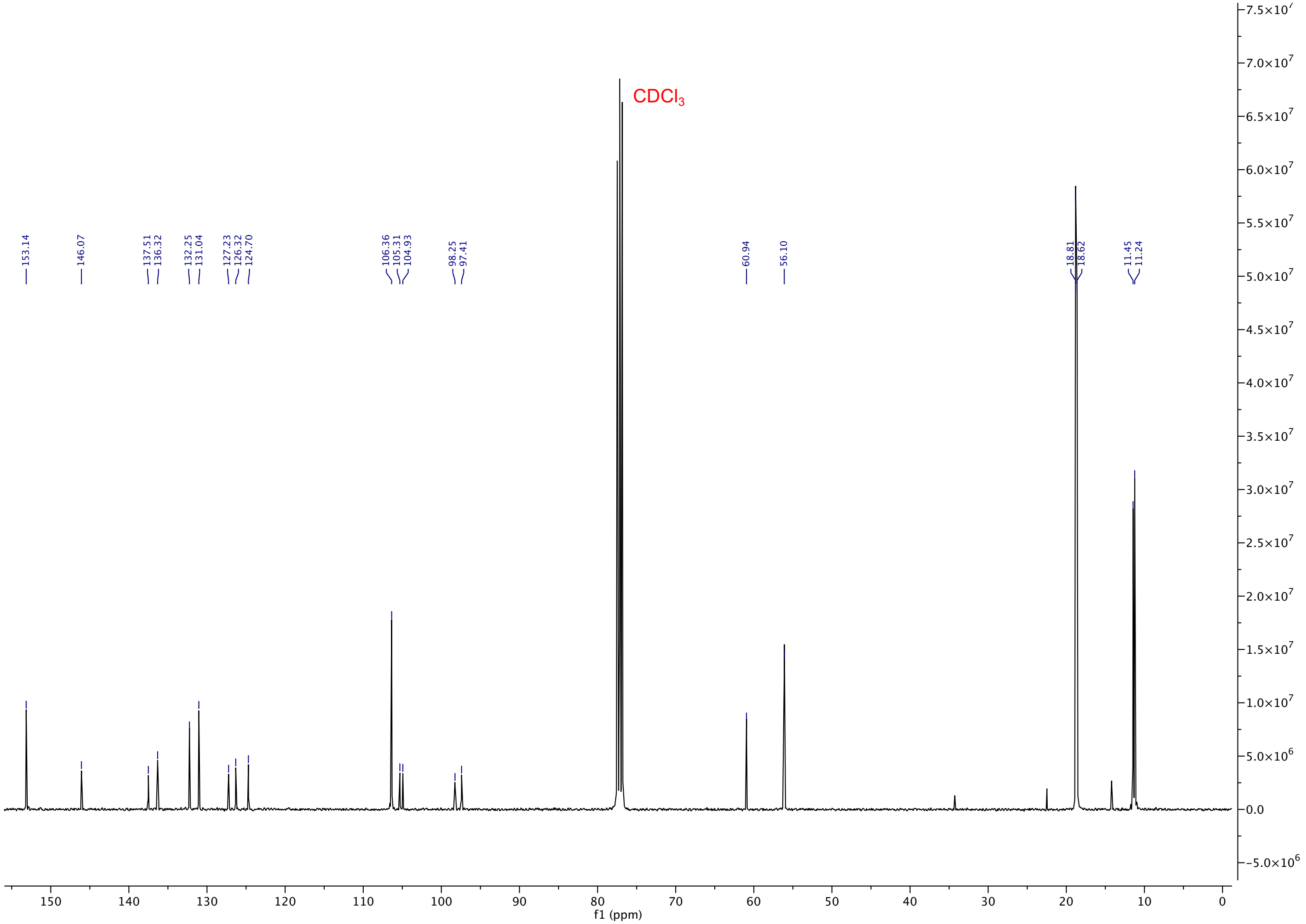
153.14
146.07
137.51
136.32
132.25
131.04
127.23
126.32
124.70
106.36
105.31
104.93
98.25
97.41
60.94
56.10
18.81
18.62
11.45
11.24
CDCl3
f1 (ppm)

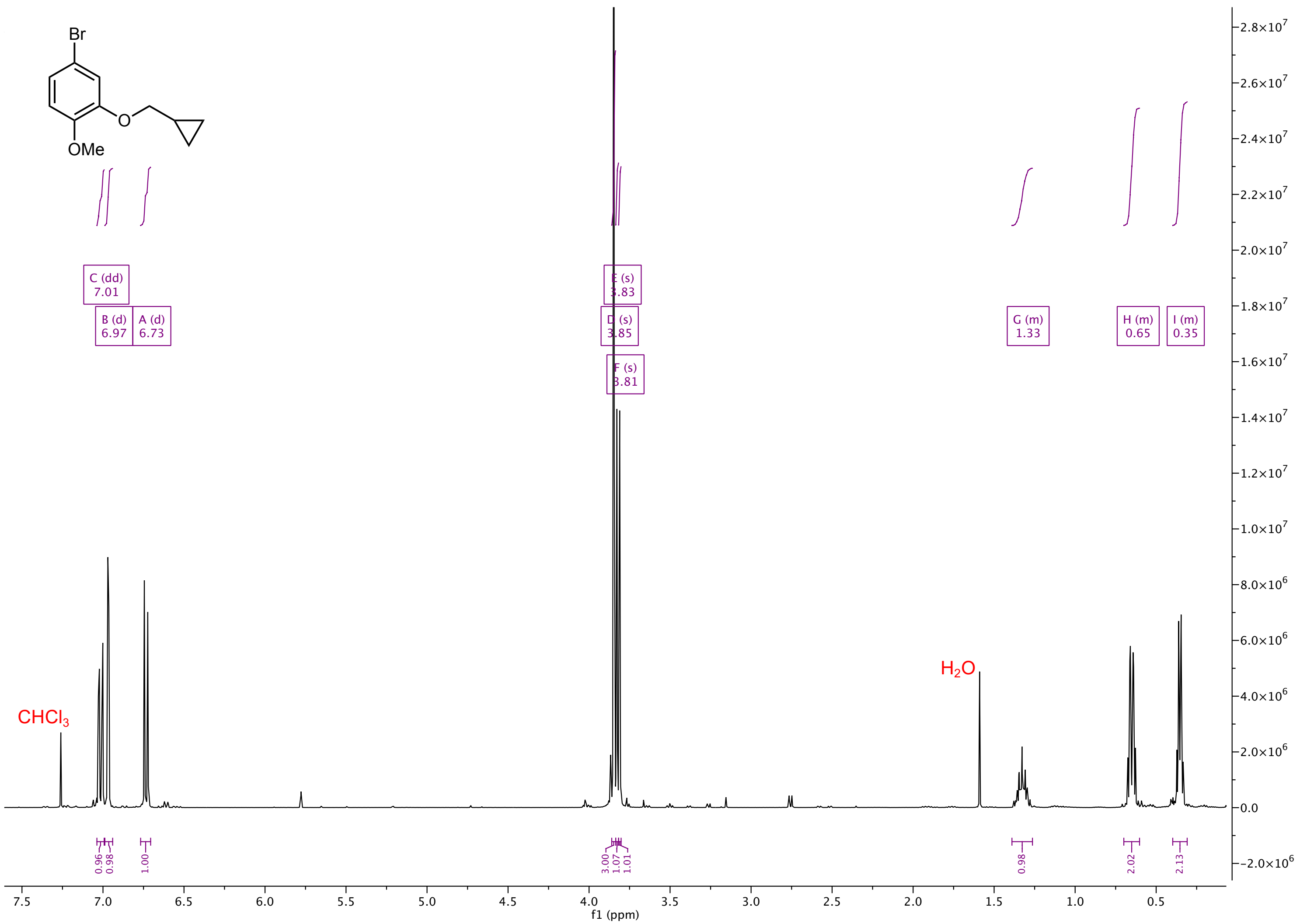

Br
O
OMe
C (dd) 7.01
B (d) 6.97
A (d) 6.73
E (s) 3.83
D (s) 3.85
F (s) 3.81
G (m) 1.33
H (m) 0.65
I (m) 0.35
CHCl3
H2O
0.96
0.98
1.00
3.00
1.07
1.01
0.98
2.02
2.13
f1 (ppm)

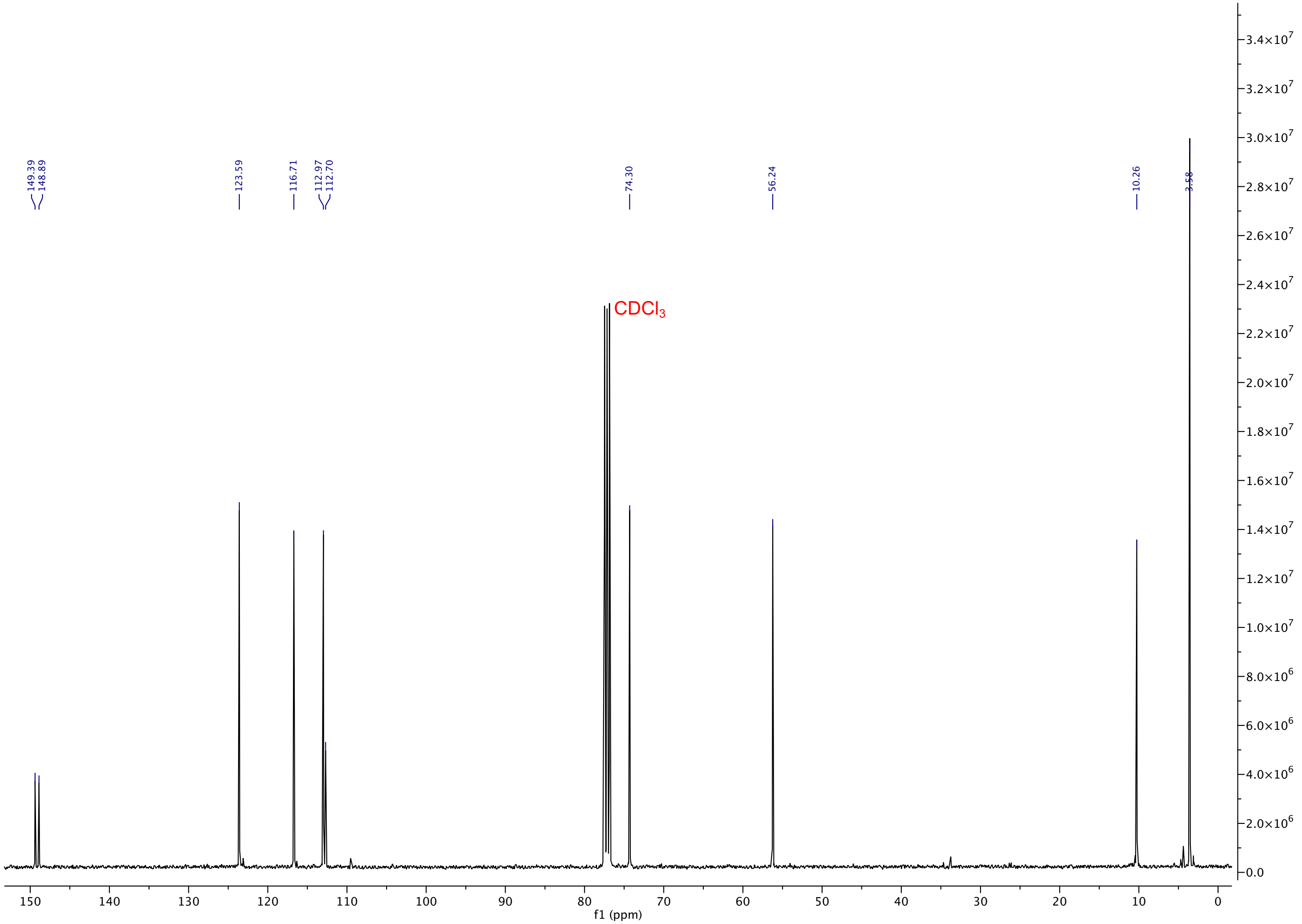

149.39
148.89
123.59
116.71
112.97
112.70
74.30
56.24
10.26
3.58
CDCl3
f1 (ppm)

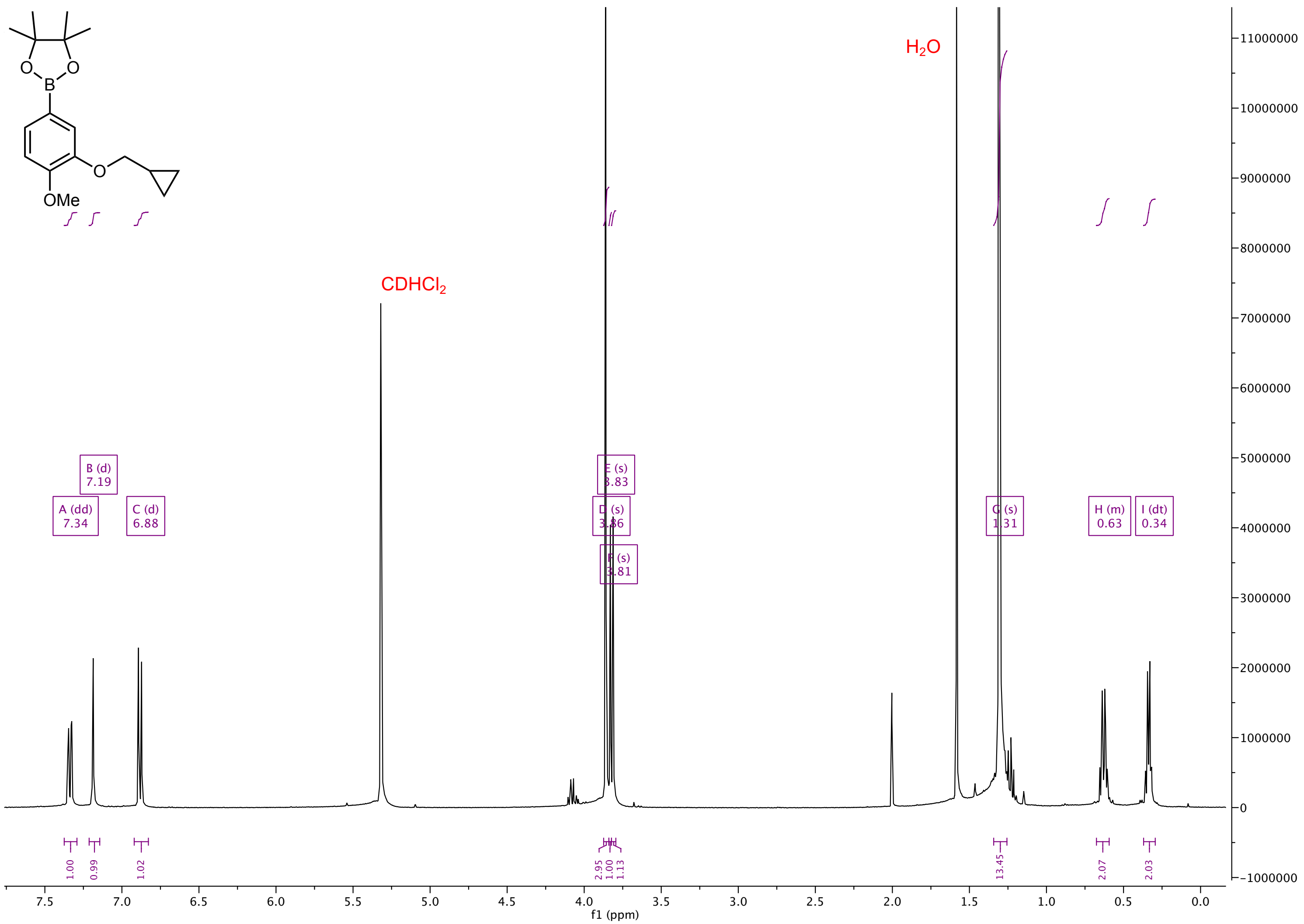

O
B
O
O
OMe
CDHCl2
H2O
B (d)
7.19
A (dd)
7.34
C (d)
6.88
E (s)
3.83
D (s)
3.86
F (s)
3.81
G (s)
1.31
H (m)
0.63
I (dt)
0.34
1.00
0.99
1.02
2.95
1.00
1.13
13.45
2.07
2.03
f1 (ppm)

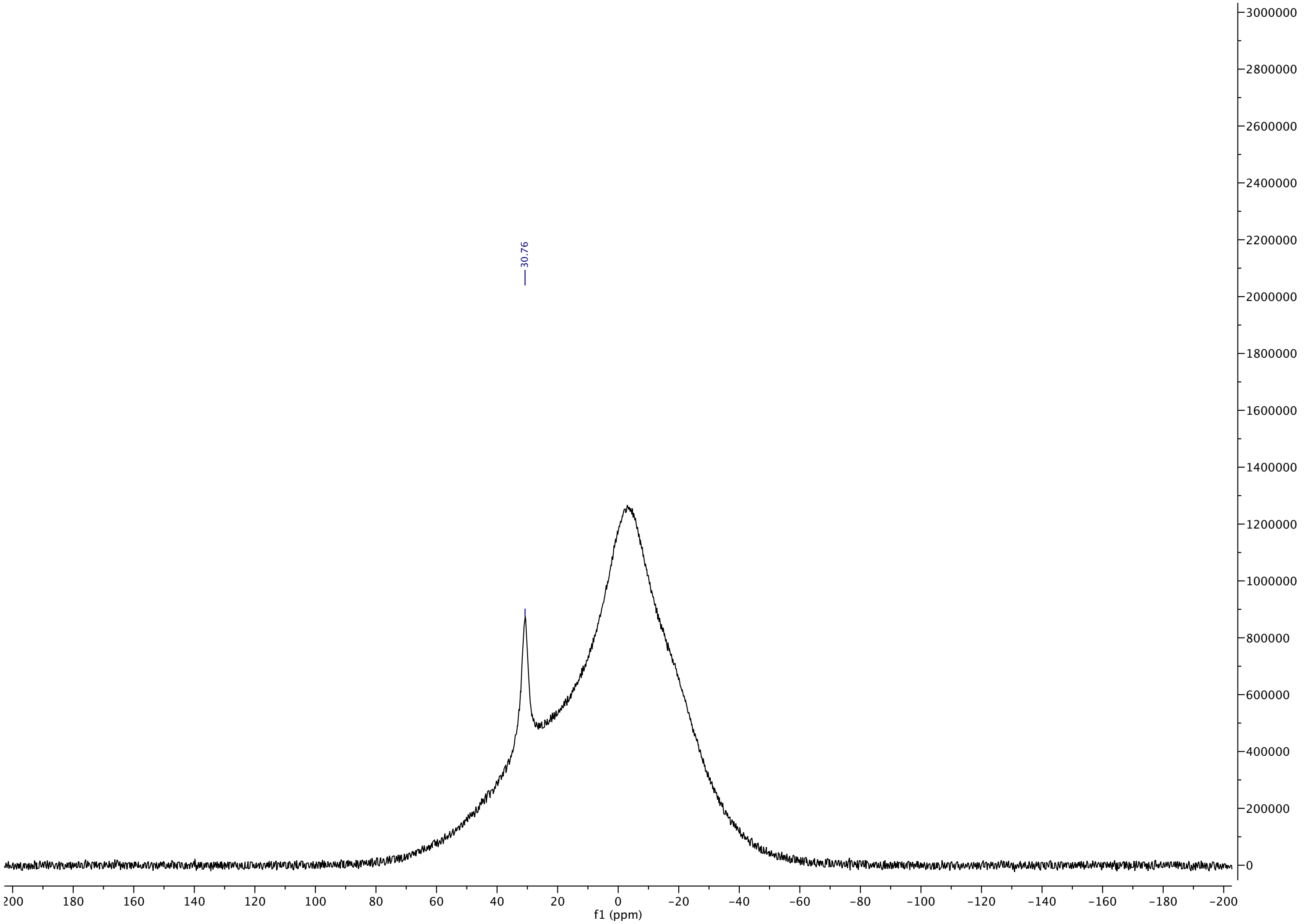
30.76
f1 (ppm)

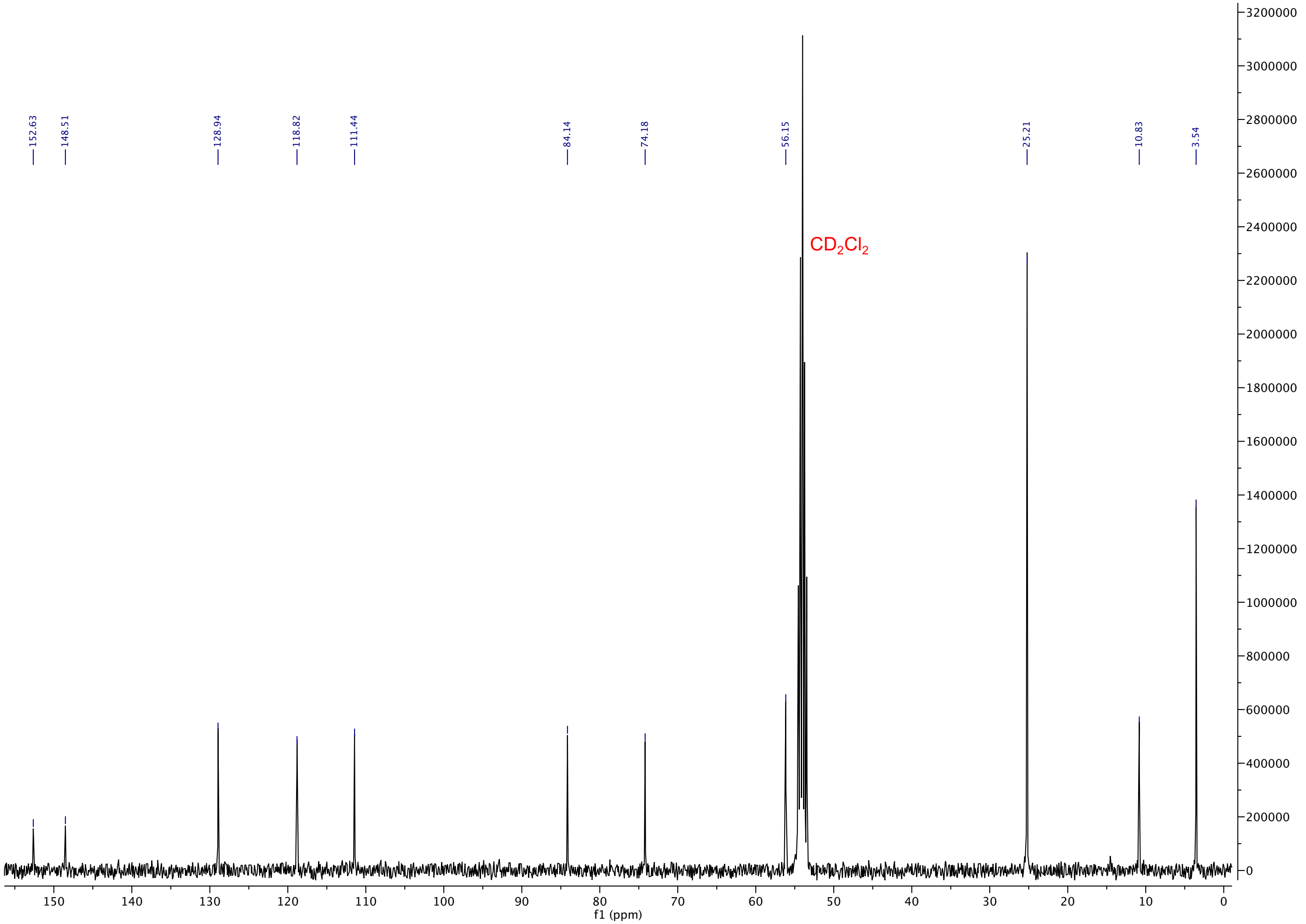
152.63
148.51
128.94
118.82
111.44
84.14
74.18
56.15
25.21
10.83
3.54
CD_2Cl_2
f1 (ppm)

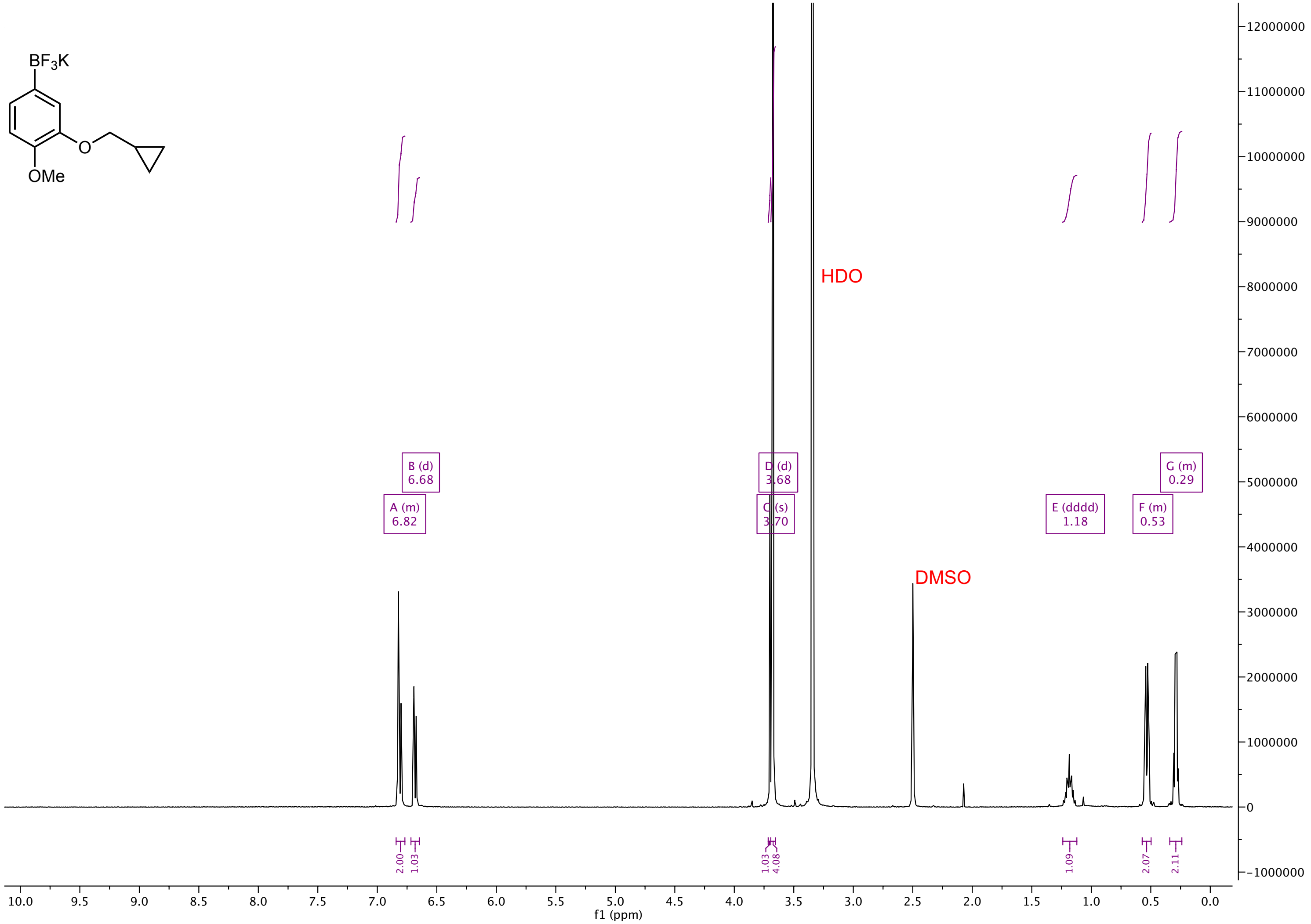
BF3K
O
OMe
HDO
DMSO
B (d)
6.68
A (m)
6.82
D (d)
3.68
C (s)
3.70
E (dddd)
1.18
F (m)
0.53
G (m)
0.29
2.00
1.03
1.03
4.08
1.09
2.07
2.11
f1 (ppm)

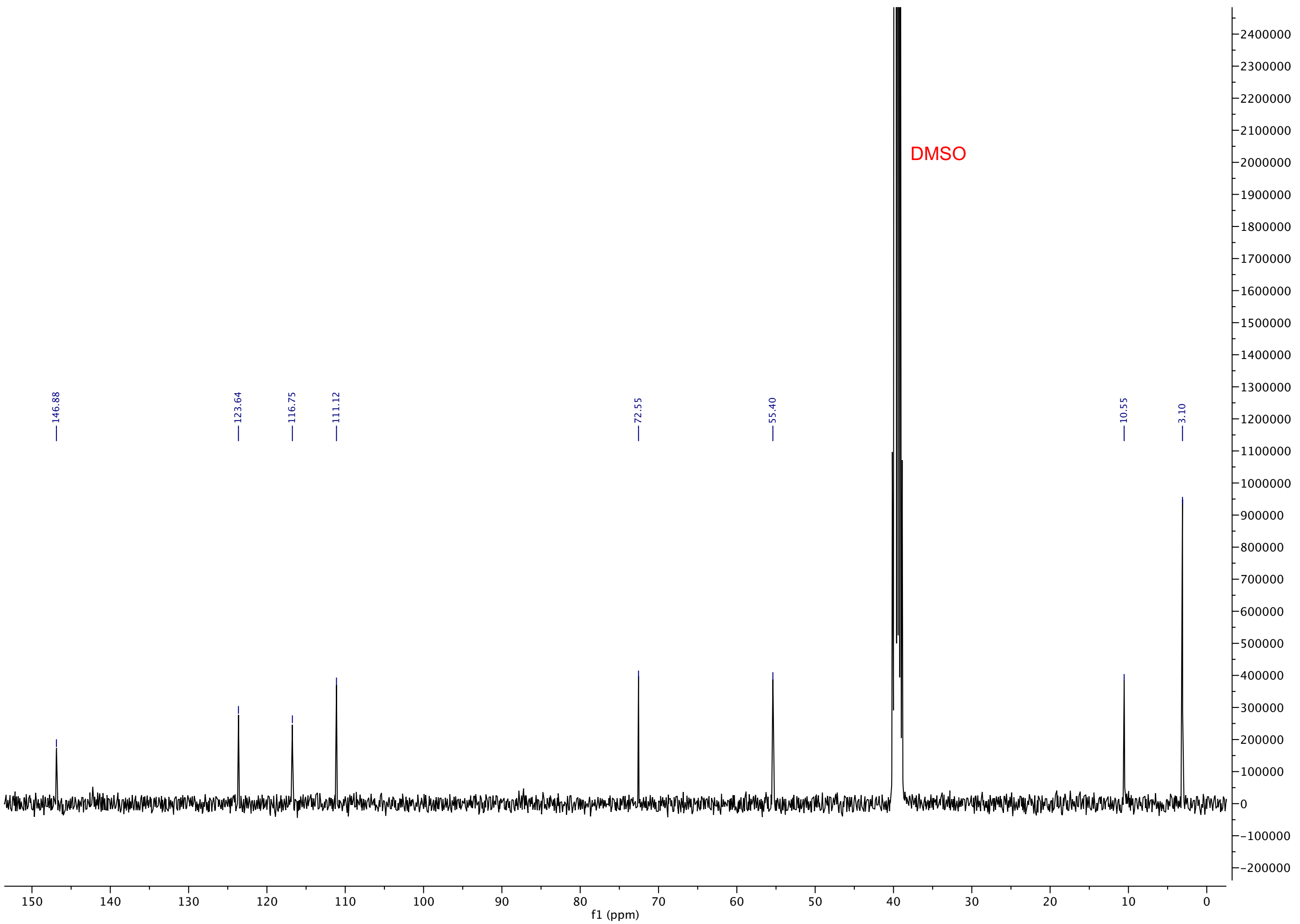

146.88
123.64
116.75
111.12
72.55
55.40
10.55
3.10
DMSO
f1 (ppm)

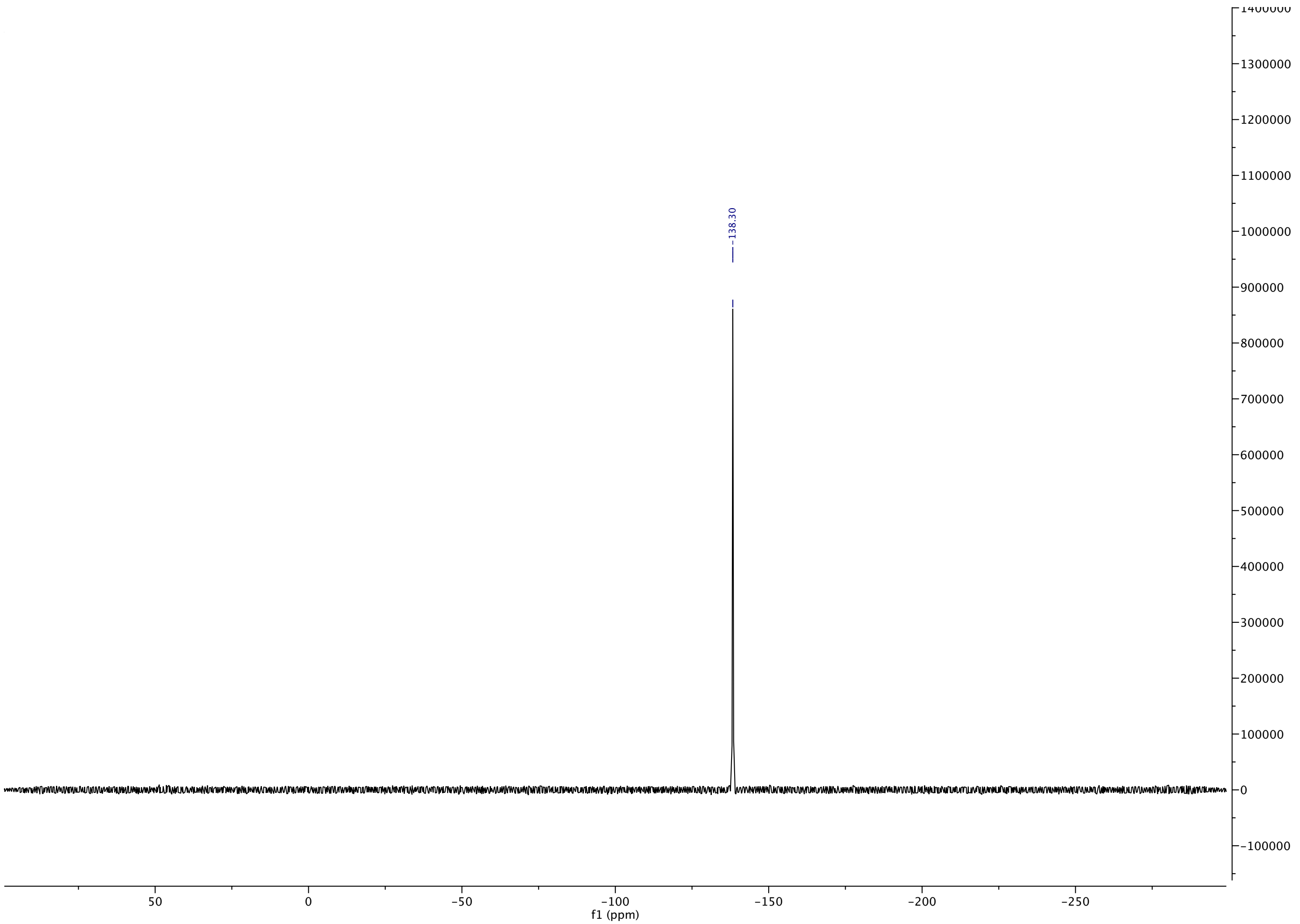

-138.30
f1 (ppm)

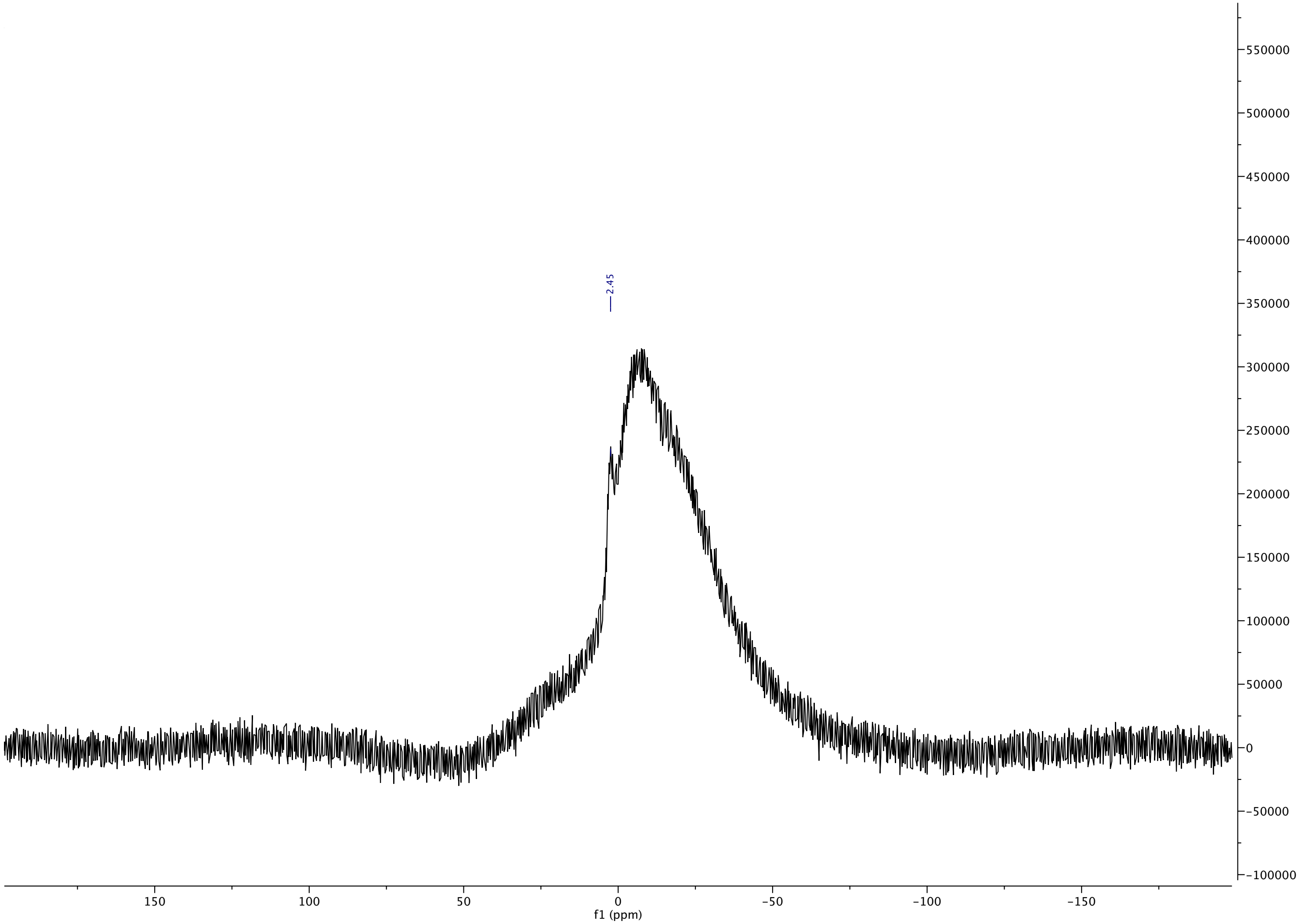

2.45
550000
500000
450000
400000
350000
300000
250000
200000
150000
100000
50000
0
−50000
−100000
150
100
50
0
−50
−100
−150
f1 (ppm)

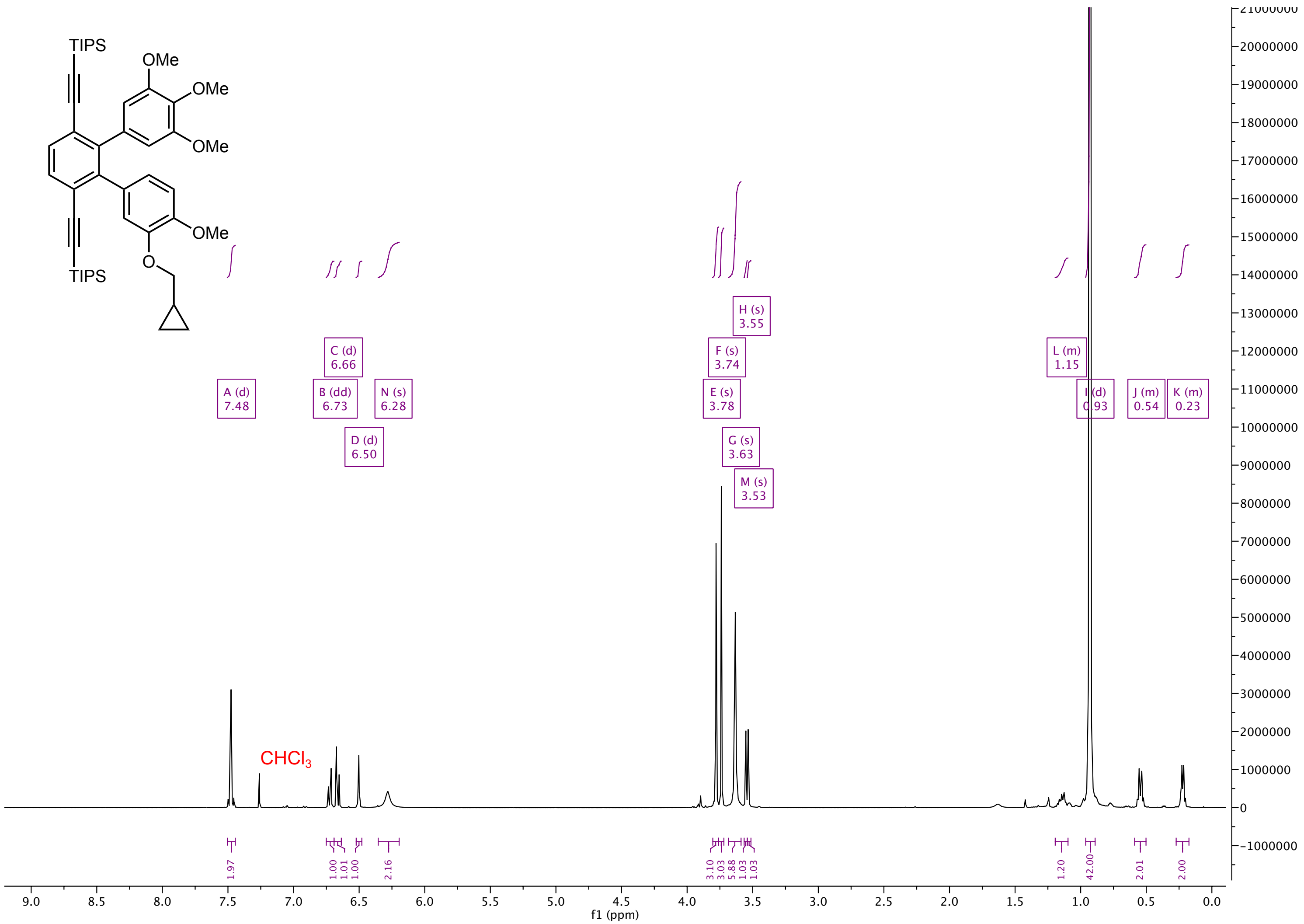
TIPS
OMe
OMe
OMe
OMe
O
TIPS
CHCl3
A (d) 7.48
B (dd) 6.73
C (d) 6.66
D (d) 6.50
N (s) 6.28
E (s) 3.78
F (s) 3.74
G (s) 3.63
H (s) 3.55
M (s) 3.53
L (m) 1.15
I (d) 0.93
J (m) 0.54
K (m) 0.23
1.97
1.00
1.01
1.00
2.16
3.10
3.03
5.88
1.03
1.03
1.20
42.00
2.01
2.00
f1 (ppm)

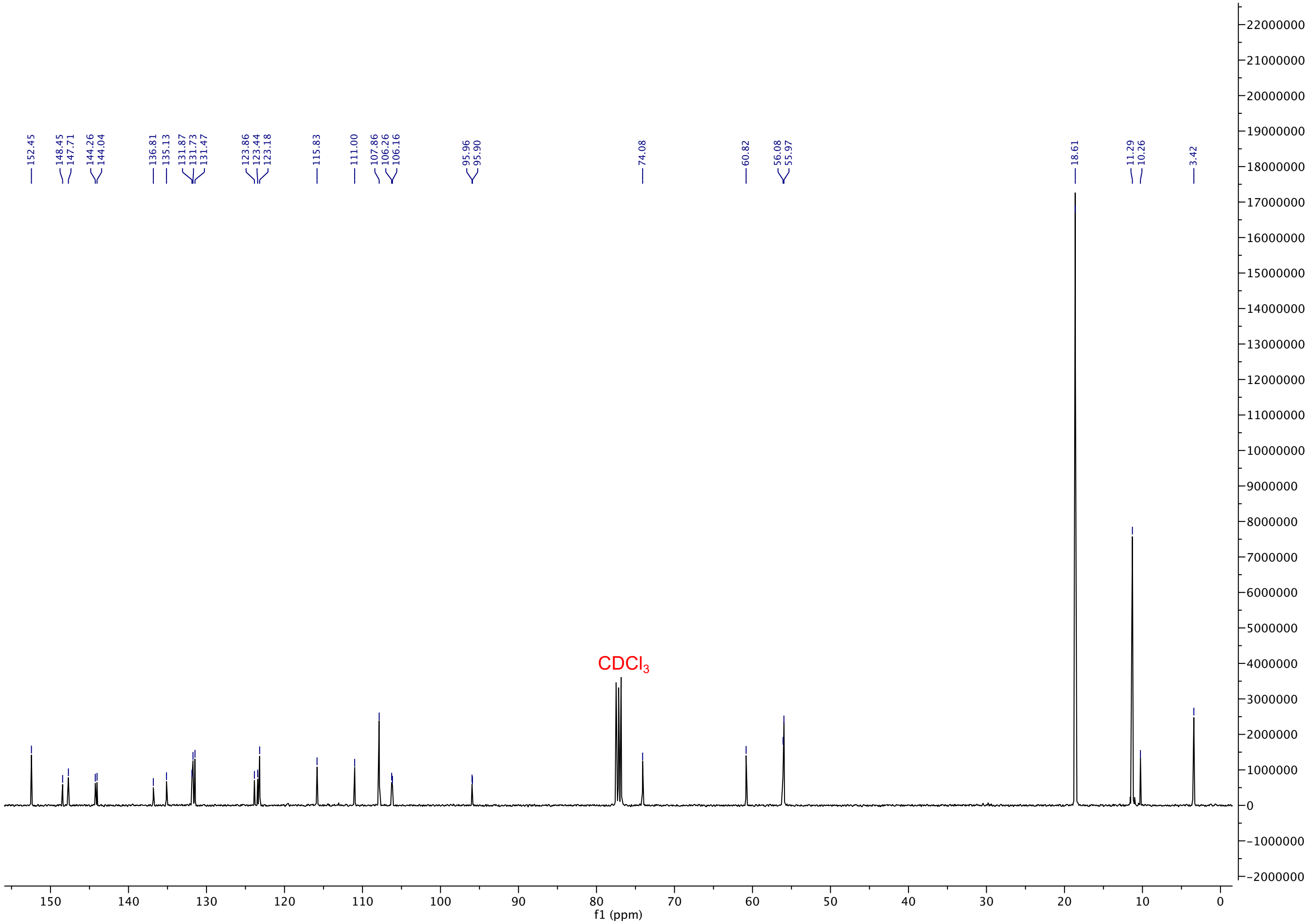
152.45
148.45
147.71
144.26
144.04
136.81
135.13
131.87
131.73
131.47
123.86
123.44
123.18
115.83
111.00
107.86
106.26
106.16
95.96
95.90
74.08
60.82
56.08
55.97
18.61
11.29
10.26
3.42
CDCl3
f1 (ppm)

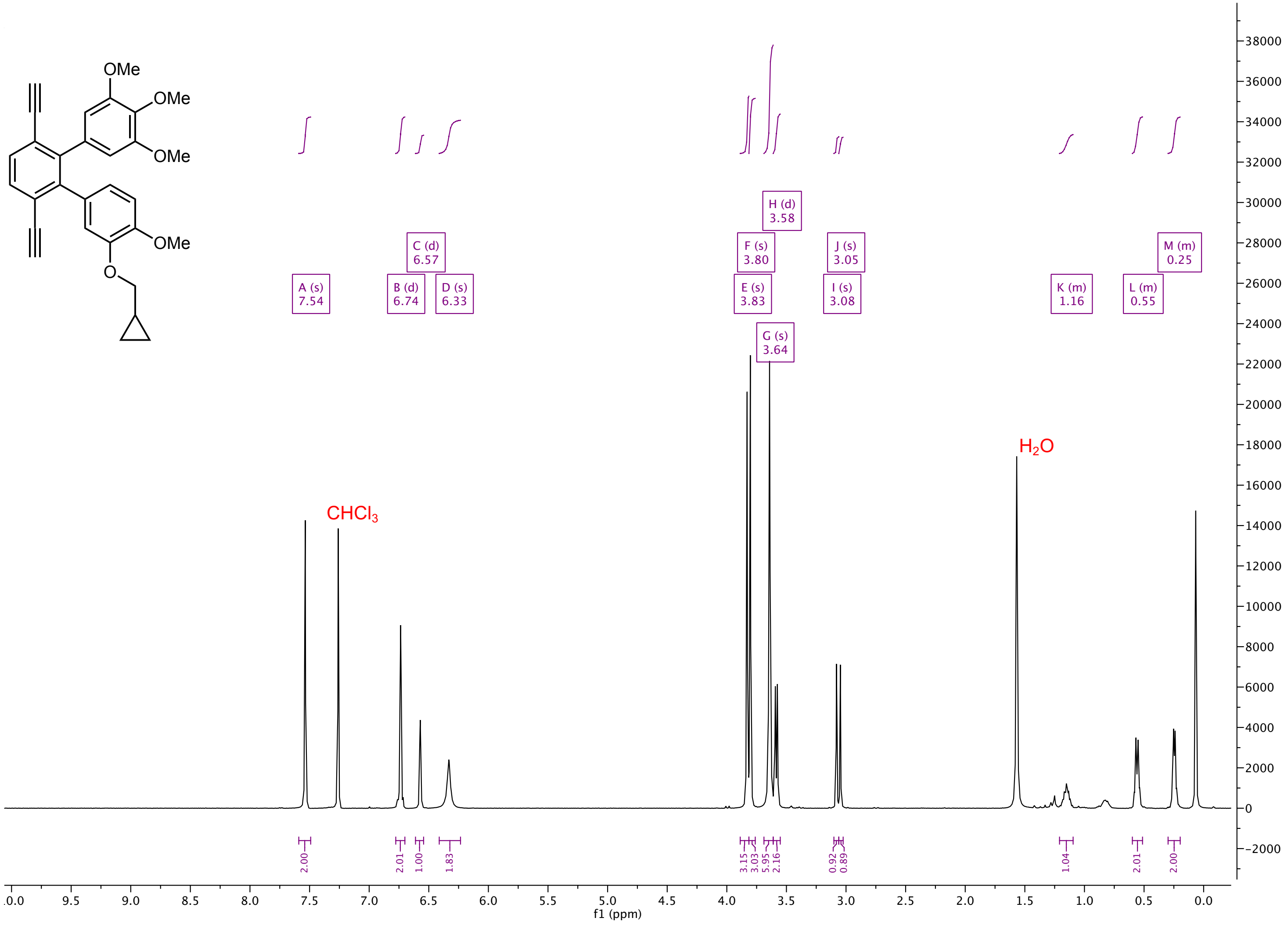
OMe
OMe
OMe
OMe
O
A (s) 7.54
B (d) 6.74
C (d) 6.57
D (s) 6.33
E (s) 3.83
F (s) 3.80
G (s) 3.64
H (d) 3.58
I (s) 3.08
J (s) 3.05
K (m) 1.16
L (m) 0.55
M (m) 0.25
CHCl3
H2O
2.00
2.01
1.00
1.83
3.15
3.03
5.95
2.16
0.92
0.89
1.04
2.01
2.00
f1 (ppm)

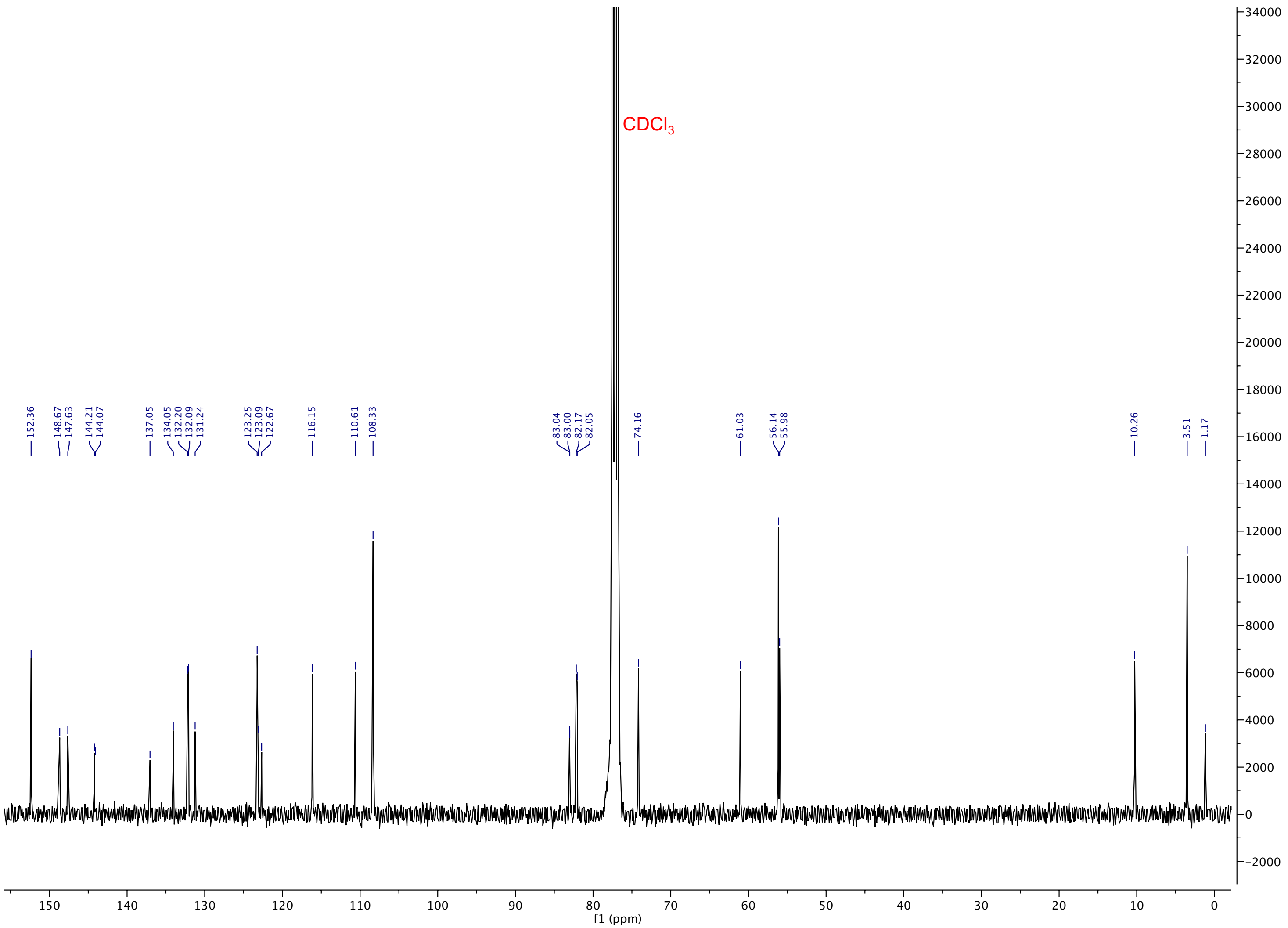
152.36
148.67
147.63
144.21
144.07
137.05
134.05
132.20
132.09
131.24
123.25
123.09
122.67
116.15
110.61
108.33
83.04
83.00
82.17
82.05
74.16
61.03
56.14
55.98
10.26
3.51
1.17
CDCl3
f1 (ppm)

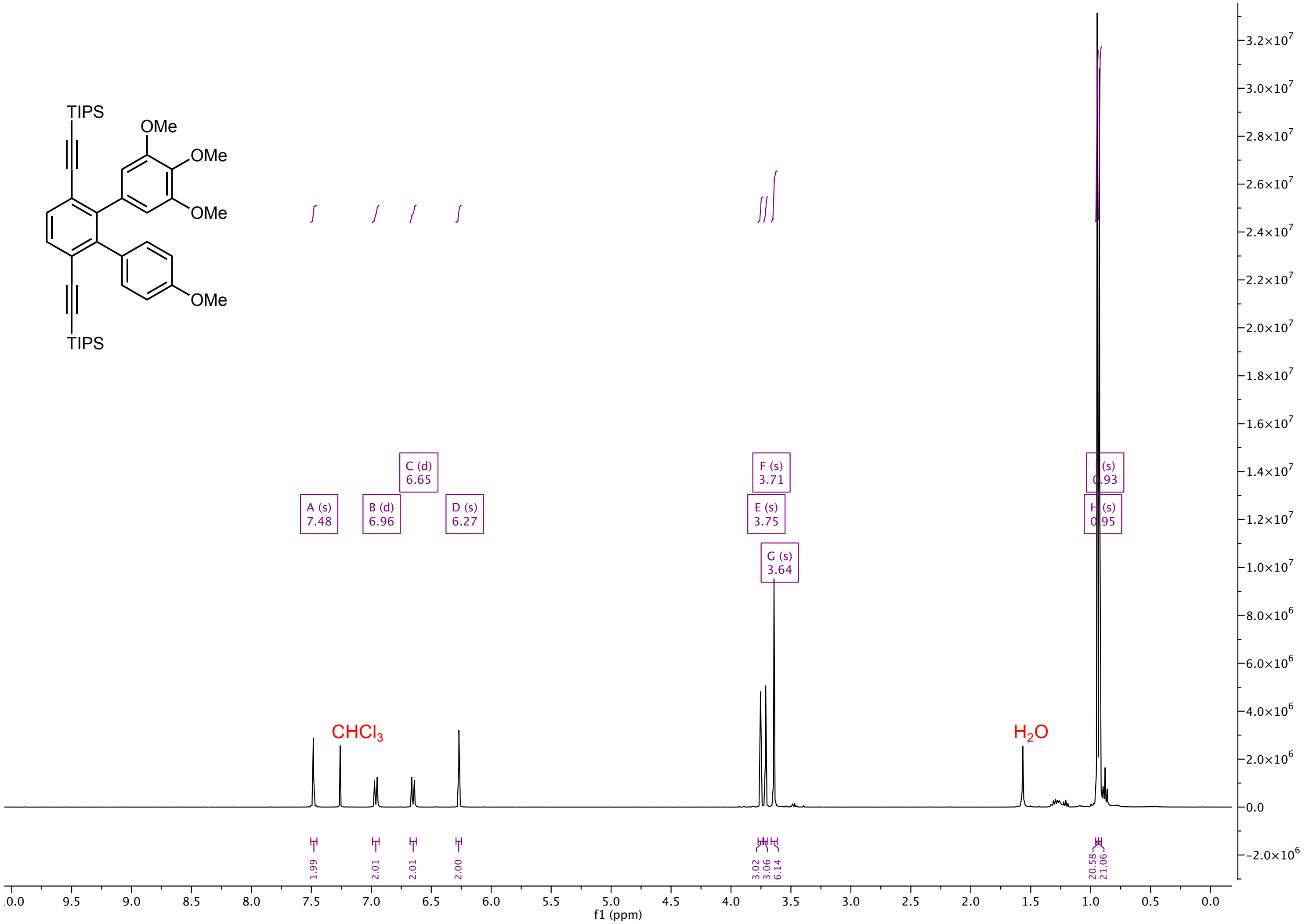
TIPS
OMe
OMe
OMe
OMe
TIPS
A (s) 7.48
B (d) 6.96
C (d) 6.65
D (s) 6.27
E (s) 3.75
F (s) 3.71
G (s) 3.64
H (s) 0.95
(s) 0.93
CHCl3
H2O
1.99
2.01
2.01
2.00
3.02
3.06
6.14
20.58
21.06
f1 (ppm)

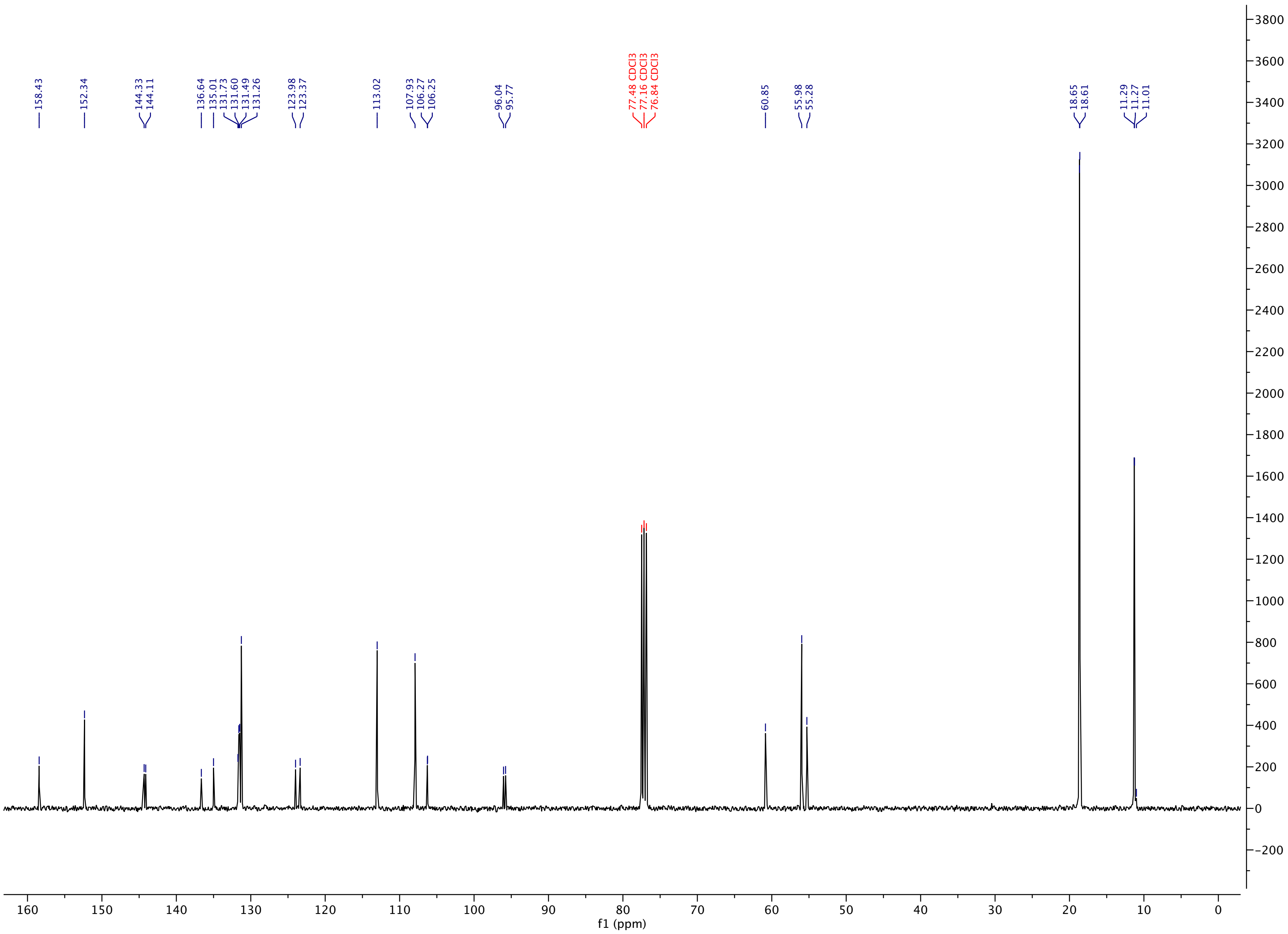
158.43
152.34
144.33
144.11
136.64
135.01
131.73
131.60
131.49
131.26
123.98
123.37
113.02
107.93
106.27
106.25
96.04
95.77
77.48 CDCl3
77.16 CDCl3
76.84 CDCl3
60.85
55.98
55.28
18.65
18.61
11.29
11.27
11.01
f1 (ppm)

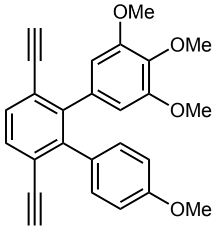
OMe
OMe
OMe
OMe

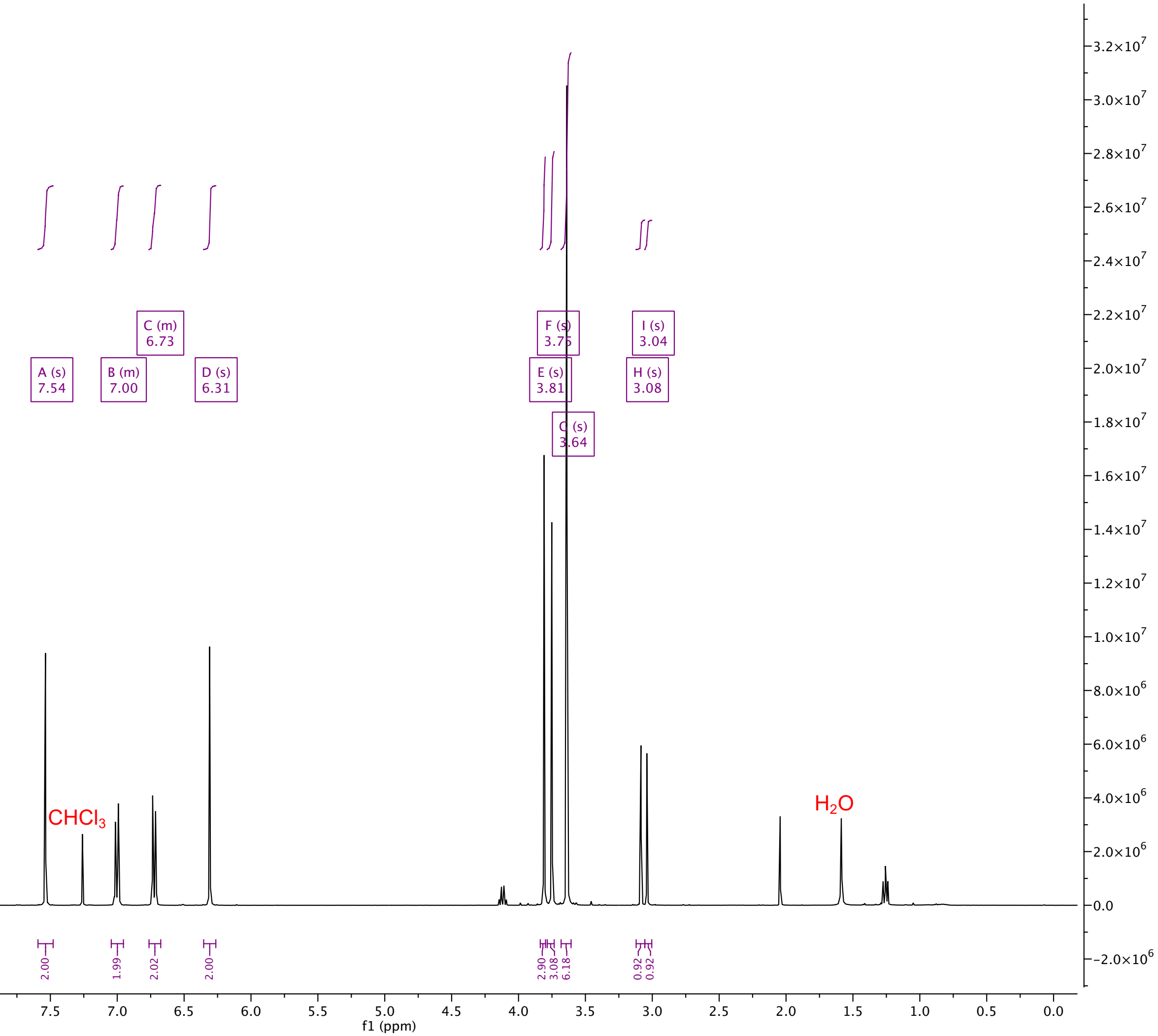
A (s)
7.54
B (m)
7.00
C (m)
6.73
D (s)
6.31
E (s)
3.81
F (s)
3.75
G (s)
3.64
H (s)
3.08
I (s)
3.04
CHCl$_3$
H$_2$O
2.00
1.99
2.02
2.00
2.90
3.08
6.18
0.92
0.92
f1 (ppm)

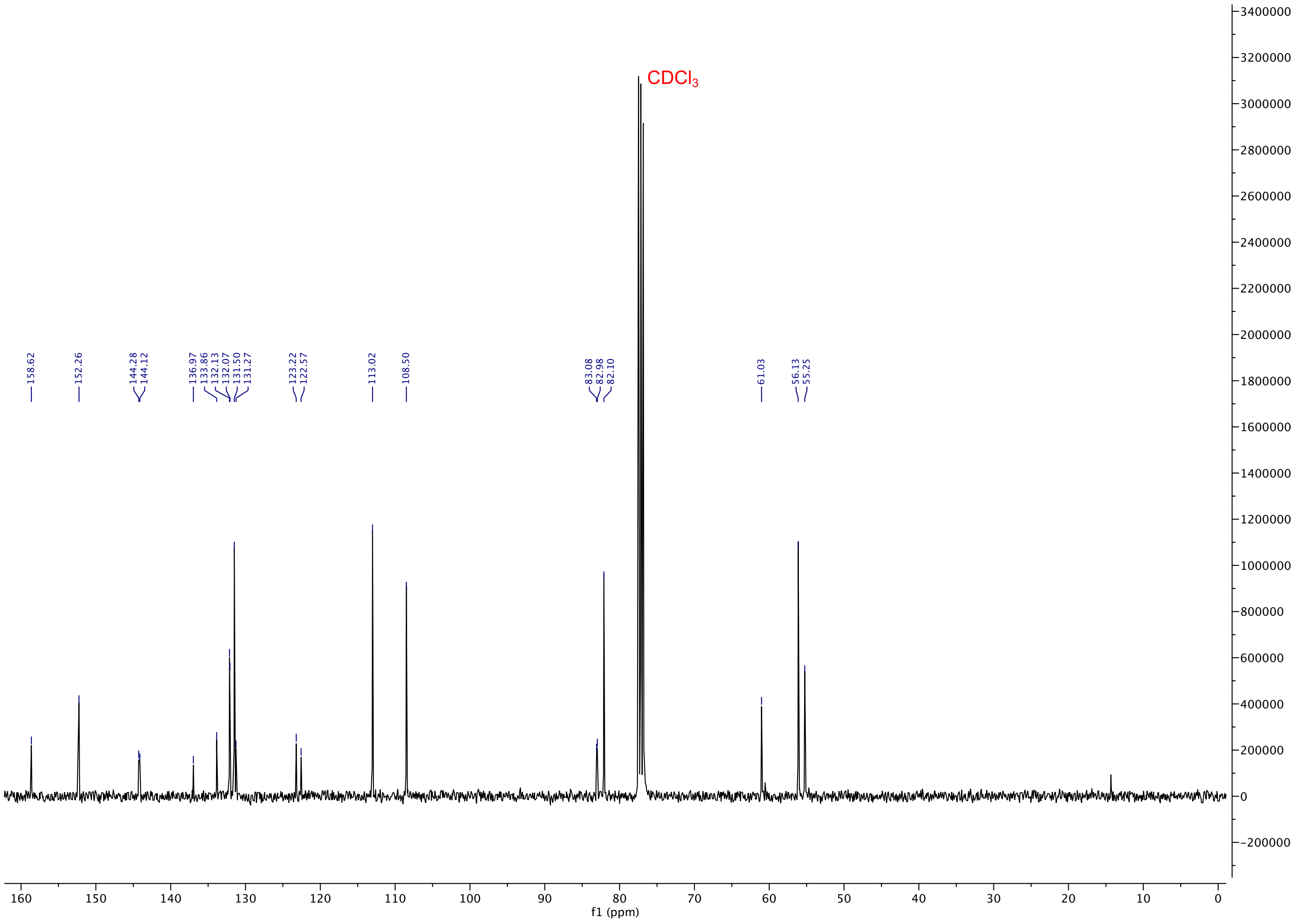
158.62
152.26
144.28
144.12
136.97
133.86
132.13
132.07
131.50
131.27
123.22
122.57
113.02
108.50
83.08
82.98
82.10
61.03
56.13
55.25
CDCl3
f1 (ppm)

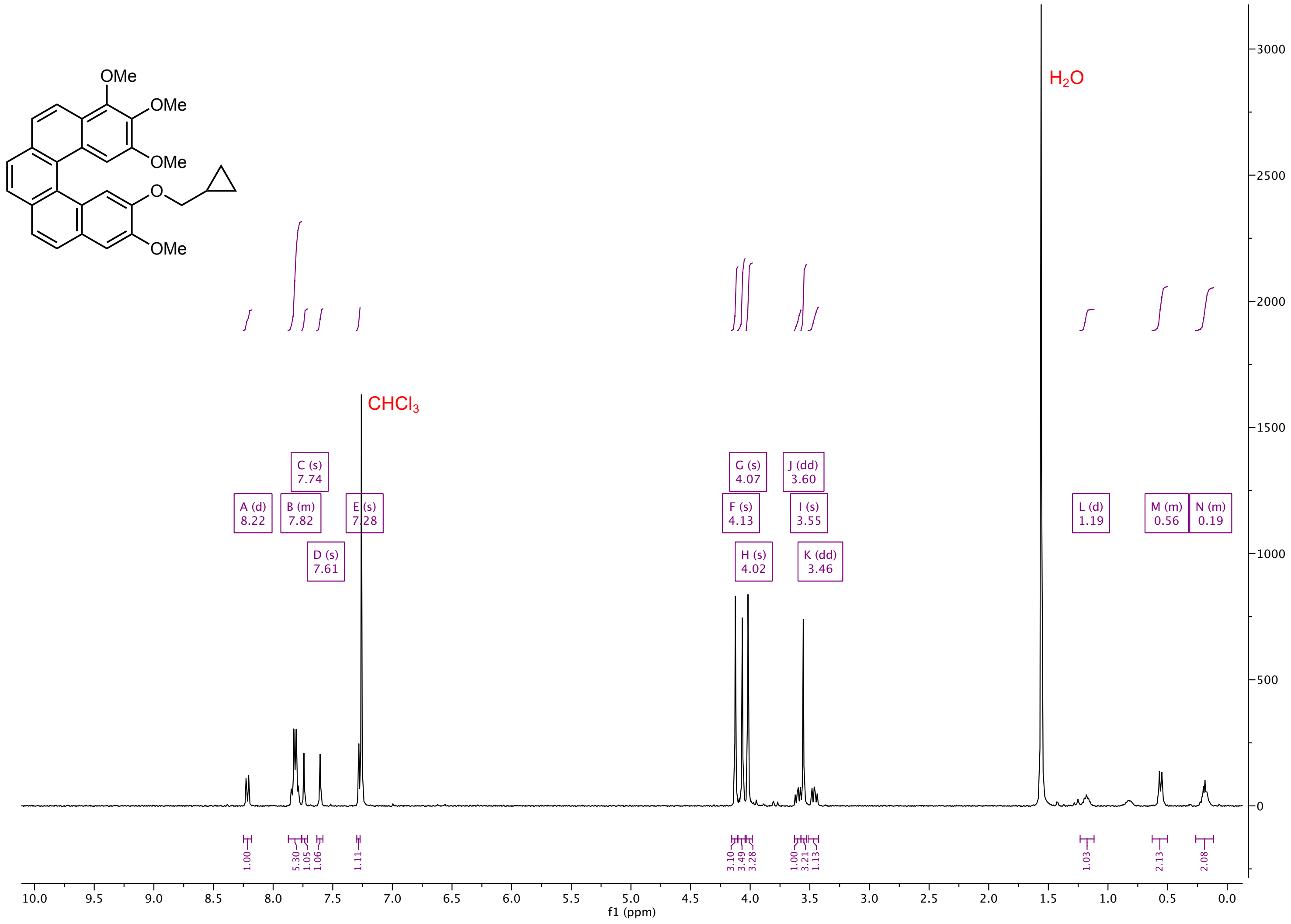
OMe
OMe
OMe
O
OMe
H$_2$O
CHCl$_3$
A (d) 8.22
B (m) 7.82
C (s) 7.74
D (s) 7.61
E (s) 7.28
F (s) 4.13
G (s) 4.07
H (s) 4.02
I (s) 3.55
J (dd) 3.60
K (dd) 3.46
L (d) 1.19
M (m) 0.56
N (m) 0.19
f1 (ppm)

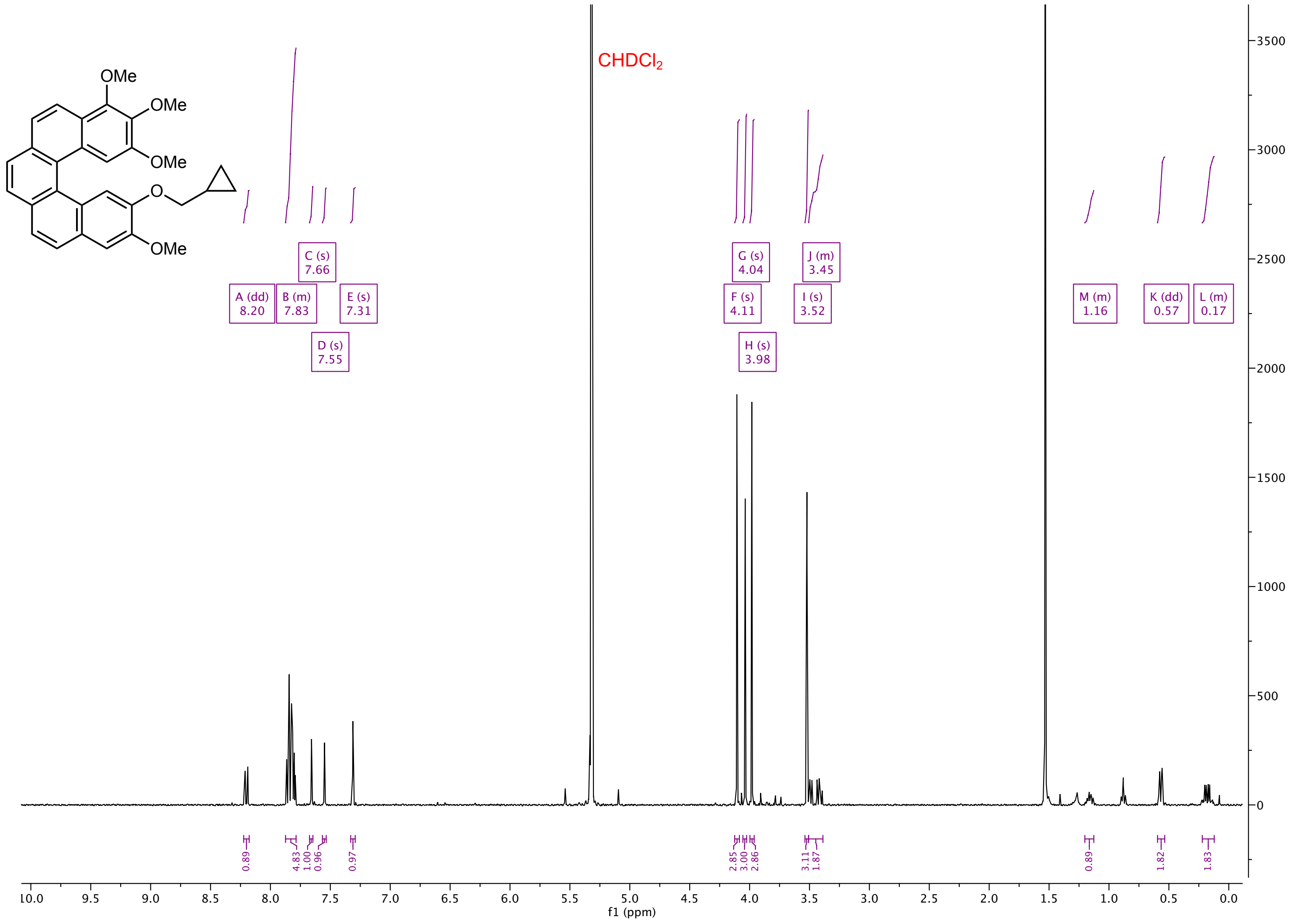

OMe
OMe
OMe
O
OMe
CHDCl2
A (dd) 8.20
B (m) 7.83
C (s) 7.66
D (s) 7.55
E (s) 7.31
F (s) 4.11
G (s) 4.04
H (s) 3.98
I (s) 3.52
J (m) 3.45
M (m) 1.16
K (dd) 0.57
L (m) 0.17
0.89
4.83
1.00
0.96
0.97
2.85
3.00
2.86
3.11
1.87
0.89
1.82
1.83
f1 (ppm)

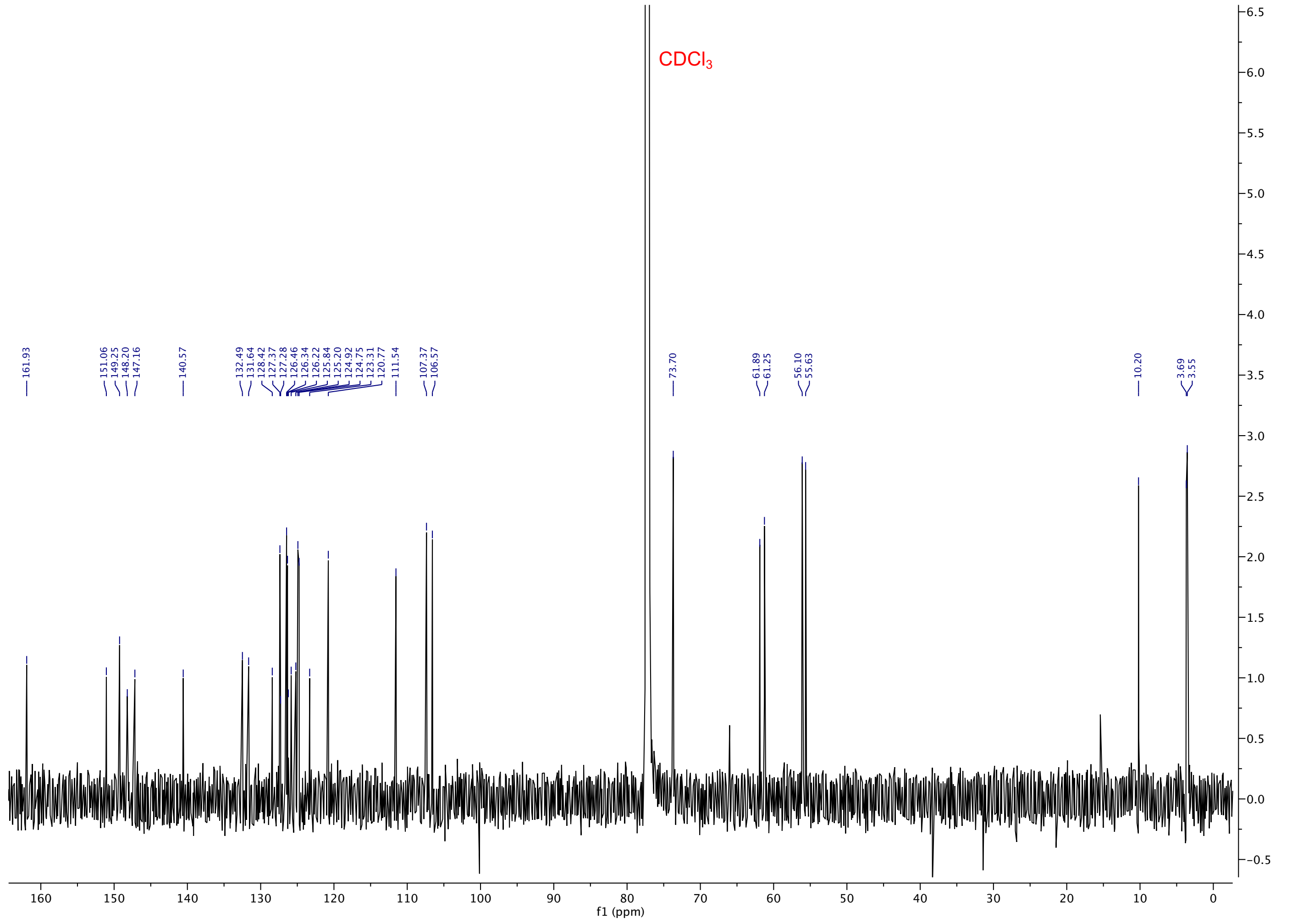
161.93
151.06
149.25
148.20
147.16
140.57
132.49
131.64
128.42
127.37
127.28
126.46
126.34
126.22
125.84
125.20
124.92
124.75
123.31
120.77
111.54
107.37
106.57
73.70
61.89
61.25
56.10
55.63
10.20
3.69
3.55
CDCl3
f1 (ppm)

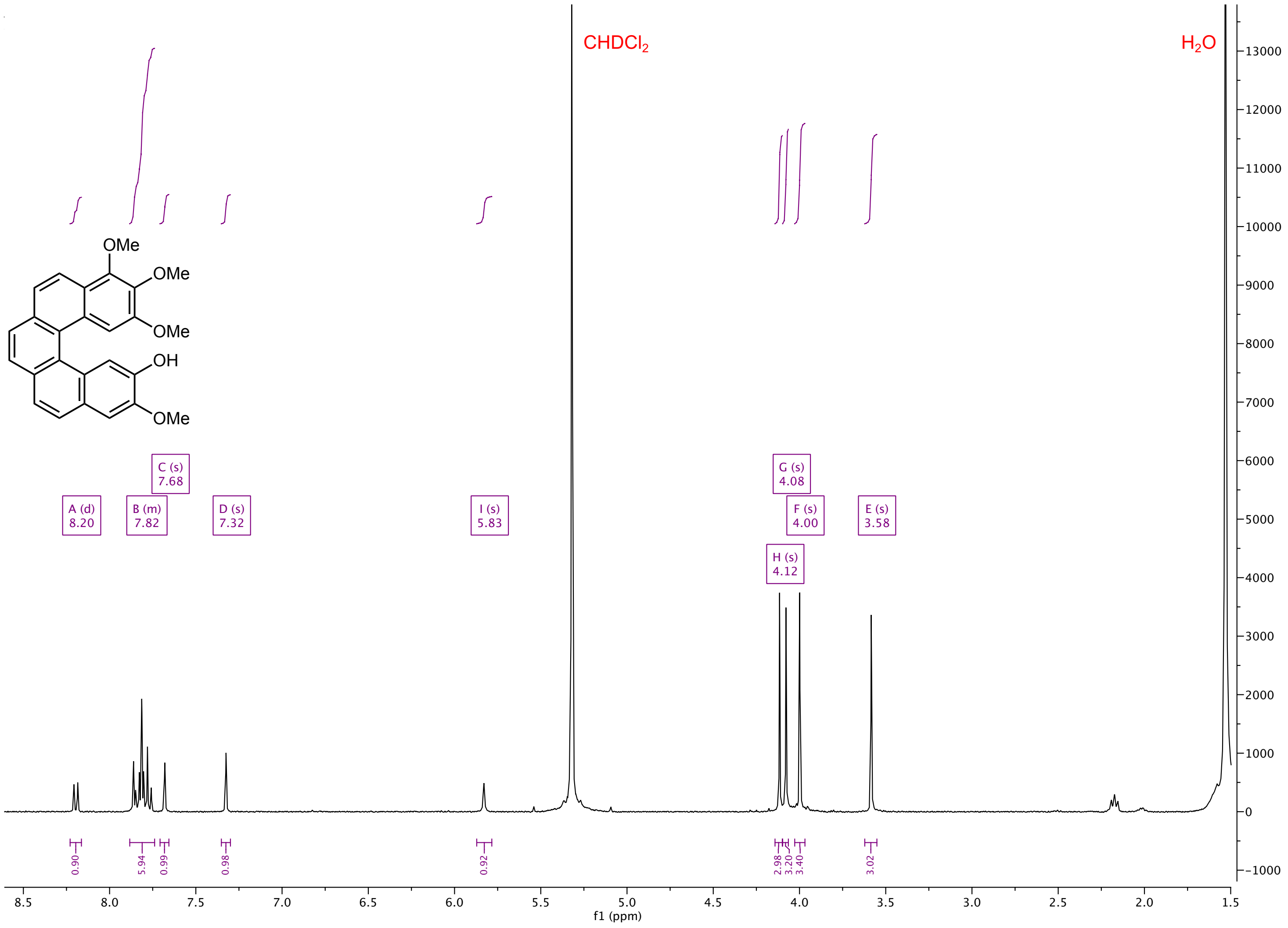
CHDCl2
H2O
OMe
OMe
OMe
OH
OMe
A (d) 8.20
B (m) 7.82
C (s) 7.68
D (s) 7.32
I (s) 5.83
G (s) 4.08
H (s) 4.12
F (s) 4.00
E (s) 3.58
0.90
5.94
0.99
0.98
0.92
2.98
3.20
3.40
3.02
f1 (ppm)

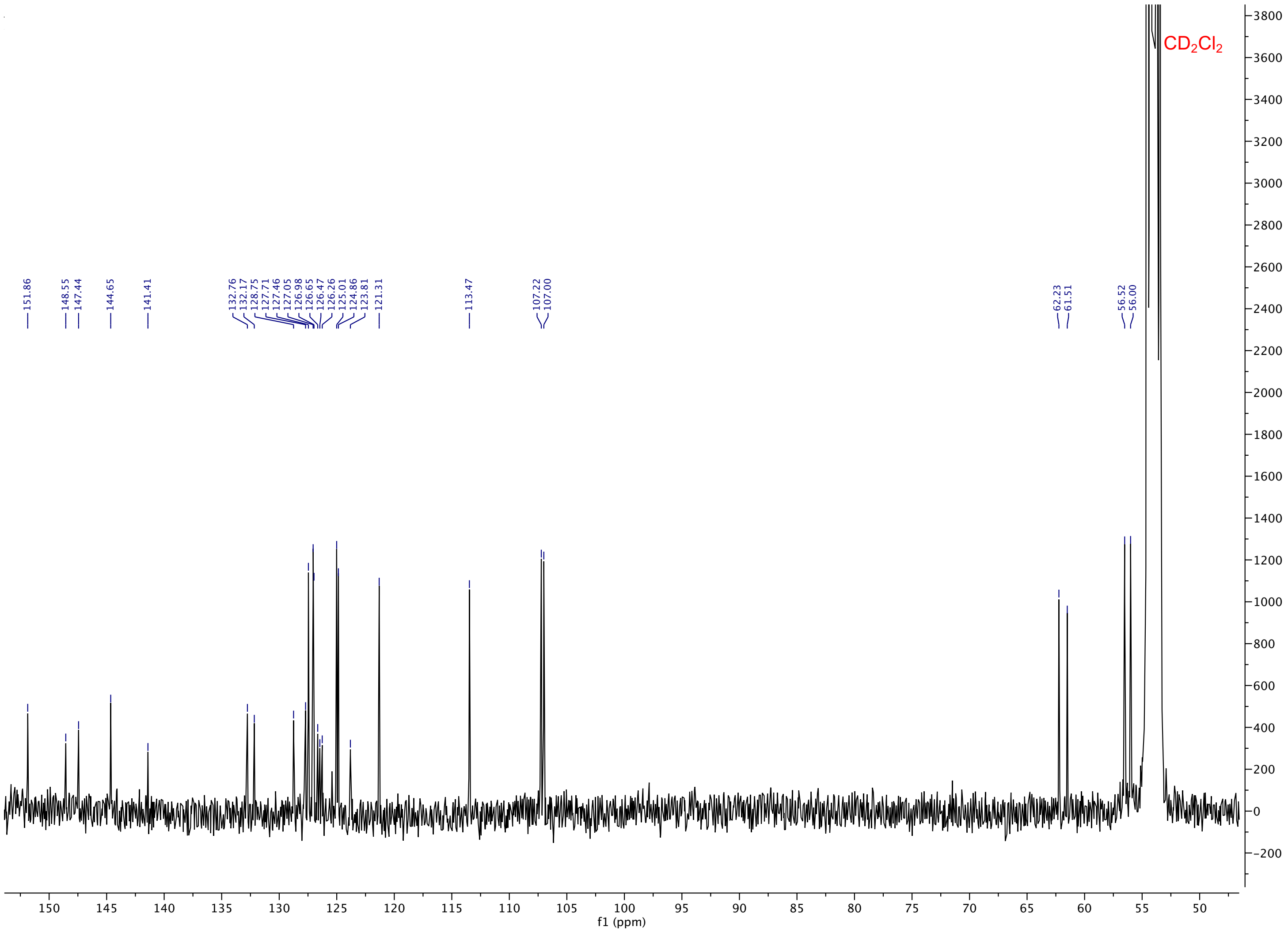
151.86
148.55
147.44
144.65
141.41
132.76
132.17
128.75
127.71
127.46
127.05
126.98
126.65
126.47
126.26
125.01
124.86
123.81
121.31
113.47
107.22
107.00
62.23
61.51
56.52
56.00
CD2Cl2
f1 (ppm)

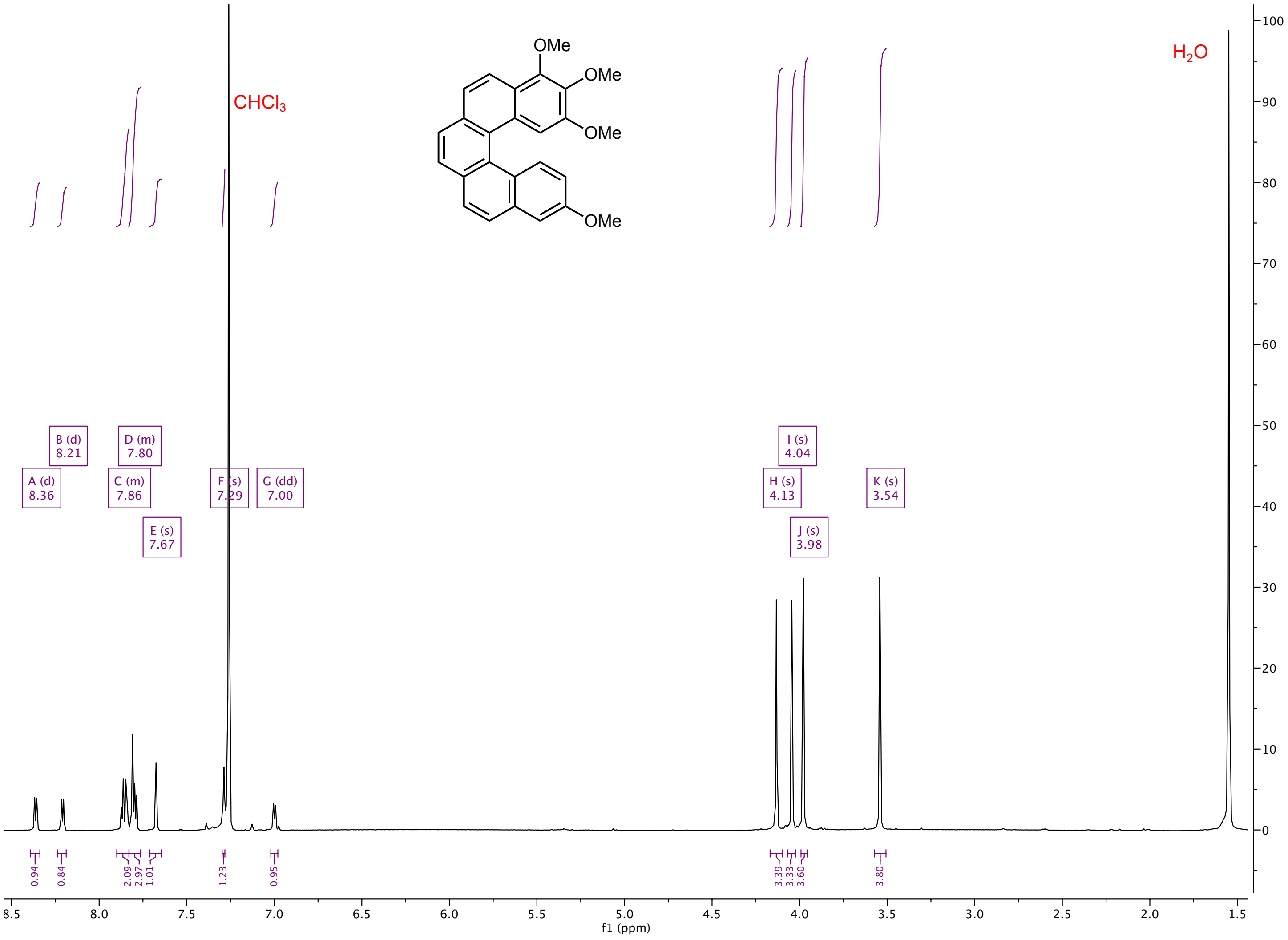
OMe
OMe
OMe
OMe
CHCl3
H2O
A (d)
8.36
B (d)
8.21
C (m)
7.86
D (m)
7.80
E (s)
7.67
F (s)
7.29
G (dd)
7.00
H (s)
4.13
I (s)
4.04
J (s)
3.98
K (s)
3.54
0.94
0.84
2.09
2.97
1.01
1.23
0.95
3.39
3.33
3.60
3.80
f1 (ppm)

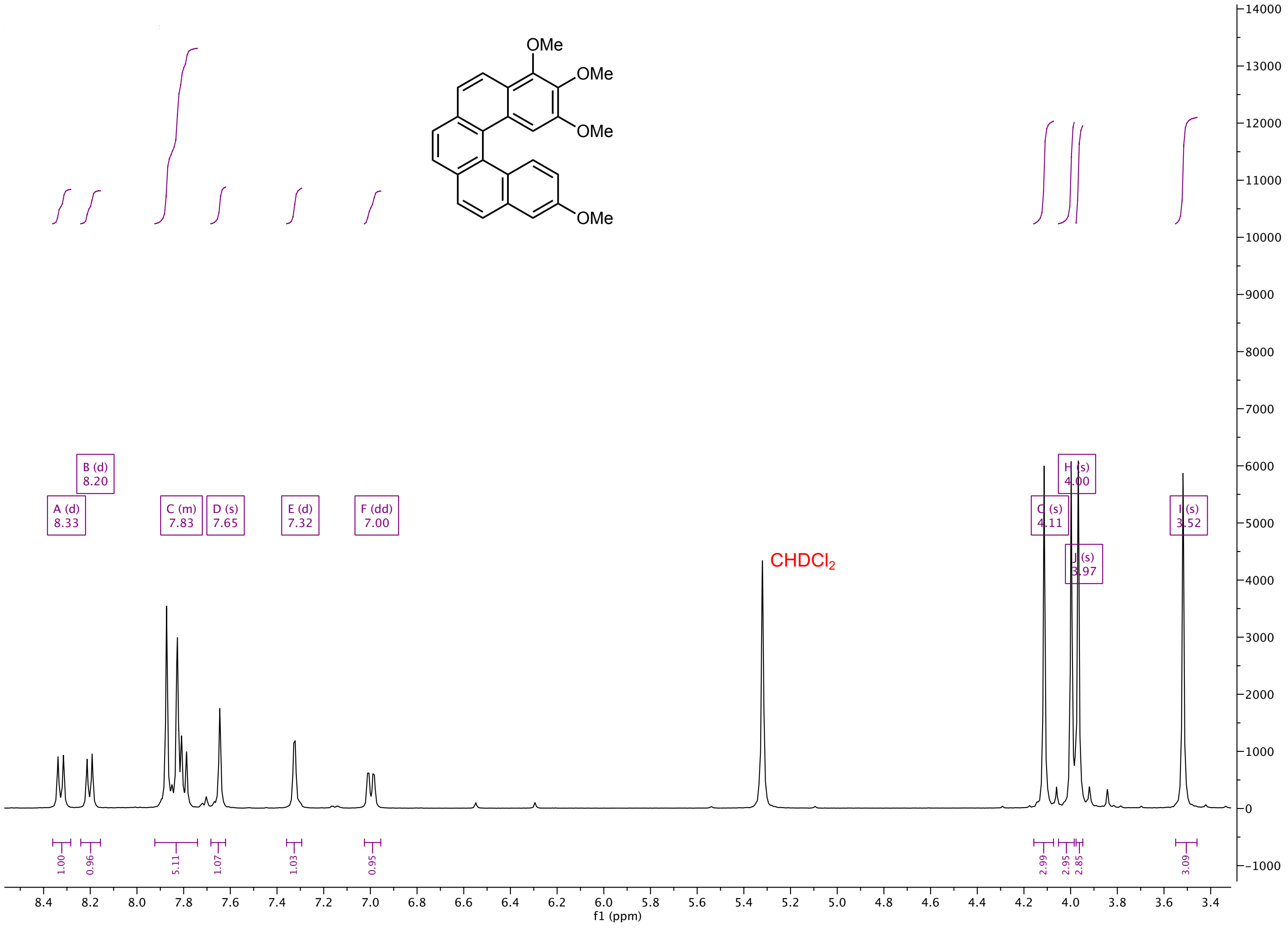
OMe
OMe
OMe
OMe
A (d)
8.33
B (d)
8.20
C (m)
7.83
D (s)
7.65
E (d)
7.32
F (dd)
7.00
CHDCl2
G (s)
4.11
H (s)
4.00
J (s)
3.97
I (s)
3.52
1.00
0.96
5.11
1.07
1.03
0.95
2.99
2.95
2.85
3.09
f1 (ppm)

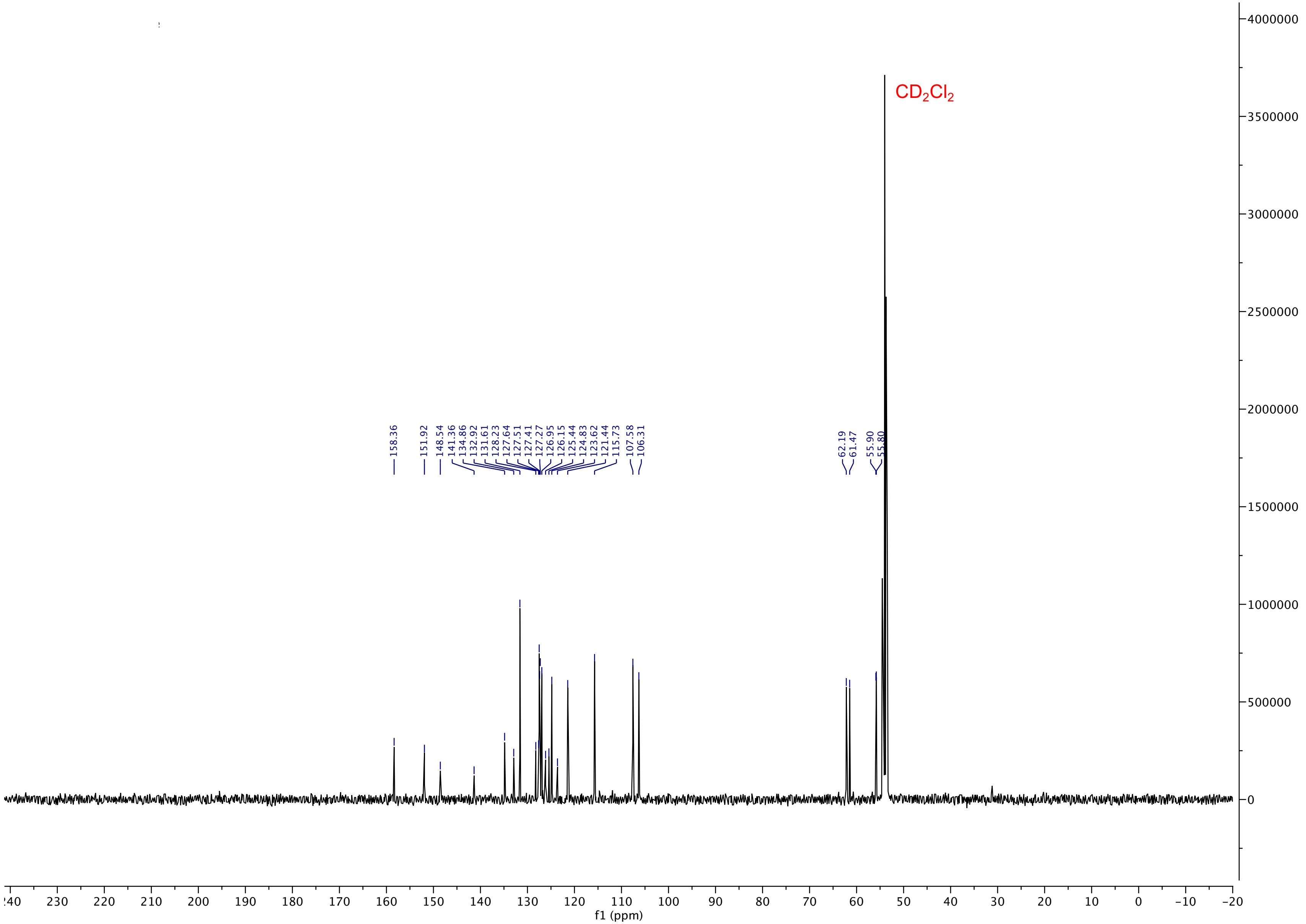
CD2Cl2
158.36
151.92
148.54
141.36
134.86
132.92
131.61
128.23
127.64
127.51
127.41
127.27
126.95
126.15
125.44
124.83
123.62
121.44
115.73
107.58
106.31
62.19
61.47
55.90
55.80
f1 (ppm)

6. References

(1) Kuhn, M.; Firth-Clark, S.; Tosco, P.; Mey, A. S. J. S.; Mackey, M.; Michel, J. Assessment of Binding Affinity via Alchemical Free-Energy Calculations. *J. Chem. Inf. Model.* **2020**, *60* (6), 3120–3130. https://doi.org/10.1021/acs.jcim.0c00165.

(2) Bauer, M. R.; Mackey, M. D. Electrostatic Complementarity as a Fast and Effective Tool to Optimize Binding and Selectivity of Protein–Ligand Complexes. *J. Med. Chem.* **2019**, *62* (6), 3036–3050. https://doi.org/10.1021/acs.jmedchem.8b01925.

(3) Cheeseright, T.; Mackey, M.; Rose, S.; Vinter, A. Molecular Field Extrema as Descriptors of Biological Activity: Definition and Validation. *J. Chem. Inf. Model.* **2006**, *46* (2), 665–676. https://doi.org/10.1021/ci050357s.

(4) Curmi, P. A.; Knossow, M.; Jourdain, I.; Lachkar, S. Insight into Tubulin Regulation from a Complex with Colchicine and a Stathmin-like Domain. *Nature* **2004**, *428* (MARCH), 198–202. https://doi.org/10.1038/nature02381.1.

(5) Prota, A. E.; Danel, F.; Bachmann, F.; Bargsten, K.; Buey, R. M.; Pohlmann, J.; Reinelt, S.; Lane, H.; Steinmetz, M. O. The Novel Microtubule-Destabilizing Drug BAL27862 Binds to the Colchicine Site of Tubulin with Distinct Effects on Microtubule Organization. *J. Mol. Biol.* **2014**, *426* (8), 1848–1860. https://doi.org/10.1016/j.jmb.2014.02.005.

(6) Liu, L.; Yang, B.; Katz, T. J.; Poindexter, M. K. Improved Methodology for Photocyclization Reactions. *J. Org. Chem.* **1991**, *56* (12), 3769–3775. https://doi.org/10.1021/jo00012a005.

(7) Kita, Y.; Gyoten, M.; Ohtsubo, M.; Tohma, H.; Takada, T. Non-Phenolic Oxidative Coupling of Phenol Ether Derivatives Using Phenyliodine(III) Bis(Trifluoroacetate). *Chem. Commun.* **1996**, No. 12, 1481. https://doi.org/10.1039/cc9960001481.

(8) Murase, M.; Kotani, E.; Okazaki, K.; Tobinga, S. Application of Iron(III) Complexes, Tris(2,2'-Bipyridyl)Iron(III) Perchlorate and Some Iron(III) Solvates, for Oxidative Aryl-Aryl Coupling Reactions. *Chem. Pharm. Bull. (Tokyo).* **1986**, *34* (8), 3159–3165. https://doi.org/10.1248/cpb.34.3159.

(9) Jin, Z.; Wang, Q.; Huang, R. Intramolecular Biaryl Oxidative Coupling of Stilbenes by Vanadium Oxytrichloride (VOCl3): Facile Synthesis of Substituted Phenanthrene Derivatives. *Synth. Commun.* **2004**, *34* (1), 119–128. https://doi.org/10.1081/SCC-120027245.

(10) Harrowven, D. C.; Guy, I. L.; Nanson, L. Efficient Phenanthrene, Helicene, and Azahelicene Syntheses. *Angew. Chemie - Int. Ed.* **2006**, *45* (14), 2242–2245. https://doi.org/10.1002/anie.200504287.

(11) Songis, O.; Míšek, J.; Schmid, M. B.; Kollárovič, A.; Stará, I. G.; Šaman, D.; Císařová, I.; Starý,

I. A Versatile Synthesis of Functionalized Pentahelicenes. *J. Org. Chem.* **2010**, *75* (20), 6889–6899. https://doi.org/10.1021/jo1013977.

(12) Luliński, S.; Serwatowski, J. Regiospecific Metalation of Oligobromobenzenes. *J. Org. Chem.* **2003**, *68* (13), 5384–5387. https://doi.org/10.1021/jo0340511.

(13) Diemer, V.; Leroux, F. R.; Colobert, F. Efficient and Complementary Methods Offering Access to Synthetically Valuable 1,2-Dibromobenzenes. *European J. Org. Chem.* **2011**, *2011* (2), 327–340. https://doi.org/10.1002/ejoc.201001217.

(14) Kim, Y. K.; Park, S. Y.; Joo, H. W.; Choi, E. S.; Paek, S. Y.; Kang, S. W.; Kim, B. G.; Lee, C. S.; Kim, S. W.; Lee, S. D. BIARYL DERIVATIVE AS GPR120 AGONIST. WO/2016/105118, 2016.

(15) Lennox, A. J. J.; Lloyd-Jones, G. C. Preparation of Organotrifluoroborate Salts: Precipitation-Driven Equilibrium under Non-Etching Conditions. *Angew. Chemie Int. Ed.* **2012**, *51* (37), 9385–9388. https://doi.org/10.1002/anie.201203930.

(16) SHELXTL v5.1, Bruker AXS, 1998.

(17) Sheldrick, G. M. Crystal Structure Refinement with SHELXL. *Acta Crystallogr. Sect. C Struct. Chem.* **2015**, *71* (1), 3–8. https://doi.org/10.1107/S2053229614024218.

(18) Kel, O.; Fürstenberg, A.; Mehanna, N.; Nicolas, C.; Laleu, B.; Hammarson, M.; Albinsson, B.; Lacour, J.; Vauthey, E. Chiral Selectivity in the Binding of [4]Helicene Derivatives to Double-Stranded DNA. *Chem. - A Eur. J.* **2013**, *19* (22), 7173–7180. https://doi.org/10.1002/chem.201203915.

(19) Goedicke, C.; Stegemeyer, H. Resolution and Racemization of Pentahelicene. *Tetrahedron Lett.* **1970**, *11* (12), 937–940. https://doi.org/10.1016/S0040-4039(01)97871-2.

(20) Ravat, P. Carbo[n]Helicenes Restricted to Enantiomerize: An Insight into the Design Process of Configurationally Stable Functional Chiral PAHs. *Chem. – A Eur. J.* **2021**, *27* (12), 3957–3967. https://doi.org/10.1002/chem.202004488.

(21) Gao, L.; Meiring, J. C. M.; Varady, A.; Ruider, I. E.; Heise, C.; Wranik, M.; Velasco, C. D.; Taylor, J. A.; Terni, B.; Standfuss, J.; Cabernard, C. C.; Llobet, A.; Steinmetz, M. O.; Bausch, A. R.; Distel, M.; Thorn-seshold, J.; Akhmanova, A.; Thorn-Seshold, O.; Andreas, R.; Distel, M.; Thorn-seshold, J.; Akhmanova, A.; Thorn-, O. In Vivo Photocontrol of Microtubule Dynamics and Integrity, Migration and Mitosis, by the Potent GFP-Imaging-Compatible Photoswitchable Reagents SBTubA4P and SBTub2M. *bioRxiv* **2021**, 2021.03.26.437160 (accessed 2022-09-18).
